# RBFOX splicing factors contribute to a broad but selective recapitulation of peripheral tissue splicing patterns in the thymus

**DOI:** 10.1101/gr.275245.121

**Published:** 2021-11

**Authors:** Kathrin Jansen, Noriko Shikama-Dorn, Moustafa Attar, Stefano Maio, Maria Lopopolo, David Buck, Georg A. Holländer, Stephen N. Sansom

**Affiliations:** 1 The Kennedy Institute of Rheumatology, University of Oxford, Oxford OX3 7FY, United Kingdom;; 2 Department of Paediatrics and the Weatherall Institute of Molecular Medicine, University of Oxford, Oxford OX3 9DS, United Kingdom;; 3 The University Children's Hospital of Basel and the Department of Biomedicine, University of Basel, 4056 Basel, Switzerland;; 4 Wellcome Centre for Human Genetics, University of Oxford, Oxford OX3 7BN, United Kingdom;; 5 Department of Biosystems Science and Engineering, ETH Zurich, 4058 Basel, Switzerland

## Abstract

Thymic epithelial cells (TEC) control the selection of a T cell repertoire reactive to pathogens but tolerant of self. This process is known to involve the promiscuous expression of virtually the entire protein-coding gene repertoire, but the extent to which TEC recapitulate peripheral isoforms, and the mechanisms by which they do so, remain largely unknown. We performed the first assembly-based transcriptomic census of transcript structures and splicing factor (SF) expression in mouse medullary TEC (mTEC) and 21 peripheral tissues. Mature mTEC expressed 60.1% of all protein-coding transcripts, more than was detected in any of the peripheral tissues. However, for genes with tissue-restricted expression, mTEC produced fewer isoforms than did the relevant peripheral tissues. Analysis of exon inclusion revealed an absence of brain-specific microexons in mTEC. We did not find unusual numbers of novel transcripts in TEC, and we show that *Aire*, the facilitator of promiscuous gene expression, promotes the generation of long “classical” transcripts (with 5′ and 3′ UTRs) but has only a limited impact on alternative splicing in mTEC. Comprehensive assessment of SF expression in mTEC identified a small set of nonpromiscuously expressed SF genes, among which we confirmed RBFOX to be present with AIRE in mTEC nuclei. Using a conditional loss-of-function approach, we show that *Rbfox2* promotes mTEC development and regulates the alternative splicing of promiscuously expressed genes. These data indicate that TEC recommission a small number of peripheral SFs, including members of the RBFOX family, to generate a broad but selective representation of the peripheral splice isoform repertoire.

T cells are essential for the generation and resolution of an adaptive immune response because they are uniquely able to distinguish between benign self and harmful non-self-antigens. Because T cell antigen receptor (TCR) specificity is generated pseudorandomly, the functional utility and self-reactivity of TCRs must be screened during T cell development in the thymus (Klein et al. 2014). This process is critically dependent on different thymic stromal cells such as thymic epithelial cells (TECs) (Abramson and Anderson 2017). In an initial round of positive selection, cortical TEC (cTEC) positively select developing T cells (thymocytes) that express TCRs of sufficient affinity for self-MHC (Klein et al. 2014). Subsequently, both cTEC and medullary TEC (mTEC) deplete thymocytes that display TCRs with high affinity for self-antigens, a process designated thymocyte negative selection. In addition, mTEC divert a subset of self-reactive T cells to a natural T regulatory cell (nTreg) fate (Stritesky et al. 2012).

To achieve thymocyte negative selection, TEC express and present a molecular mirror of an individual's self-antigens to maturing thymocytes. This process of promiscuous gene expression (PGE) is essential for the avoidance of autoimmunity and involves transcription of ∼89% of protein-coding genes by the TEC population (Sansom et al. 2014; Abramson and Anderson 2017). The mechanisms by which TEC defy developmental and tissue-specific transcriptional controls to achieve PGE are only incompletely deciphered (Abramson and Goldfarb 2016), although the autoimmune regulator (AIRE) (Sansom et al. 2014) and the transcription factor FEZF2 (Takaba et al. 2015) together with the chromatin remodeler CHD4 (Tomofuji et al. 2020) are known to enable PGE. Comprehensiveness of self-representation by TEC cannot, however, be measured by gene number alone because self-peptidome diversity is elaborated by alternative mRNA splicing (Anderson et al. 2000; Klein et al. 2000; Wang and Burge 2008), expression of “untranslated” regions (Starck and Shastri 2016), RNA-editing (Danan-Gotthold et al. 2016), proteasome-mediated splicing (Granados et al. 2015), and posttranslational modifications (Raposo et al. 2018). In particular, alternative mRNA splicing greatly increases the complexity of the mammalian proteome: there are approximately three times more annotated protein-coding transcripts than genes in mice and humans (Zerbino et al. 2018).

The generation of alternative splice isoforms is tightly regulated during development and controlled by temporal and context-specific expression of SFs. The extent to which TEC reproduce peripheral splice isoforms is unclear, but the transcriptome of TEC has been found to be unusually complex both in terms of isoform number (Keane et al. 2015) and splice junction representation (Danan-Gotthold et al. 2016). These surveys, which compared TEC with limited numbers of peripheral tissues, reported that TEC reproduce a fifth of tissue-restricted isoforms (Keane et al. 2015) or only 20%–60% of tissue-restricted splice junctions (Danan-Gotthold et al. 2016). Even less is known about the mechanisms in TECs that control alternative splicing. Because of its direct interactions with several splicing-related factors (Abramson et al. 2010), it was thought that AIRE might be involved in this process (Keane et al. 2015), but limited evidence supporting this idea suggests only a minor role (Danan-Gotthold et al. 2016). Instead, it is appealing to postulate that mTEC may reuse peripheral SFs to create tissue-specific splice variants. In one plausible model, mTEC might constitutively express a specific subset of peripheral SFs to achieve robust coverage of a limited repertoire of peripheral splice variants. In support of this possibility, an initial analysis identified seven RNA-binding factors with constitutive expression in murine mTEC (St-Pierre et al. 2015). In a second alternative model, mTEC might use promiscuously expressed SFs to achieve a broader stochastic coverage of peripheral isoforms. A possible side effect of such a promiscuous splicing strategy would be the generation of spurious novel isoforms if nascent transcripts interact with SFs in TEC to which they are not exposed in the periphery.

Knowledge of splice isoform representation in the thymus is important for understanding central tolerance because it is known that the absence of tissue-specific splice isoforms can result in the development of pathogenic T cells able to incite autoimmunity (Anderson et al. 2000; Klein et al. 2000). We therefore set out to perform the first comprehensive and unbiased census of isoform representation and SF usage in TEC and peripheral tissues.

## Results

### TEC express fewer splice isoforms per gene than peripheral tissues

**Figure 1.**
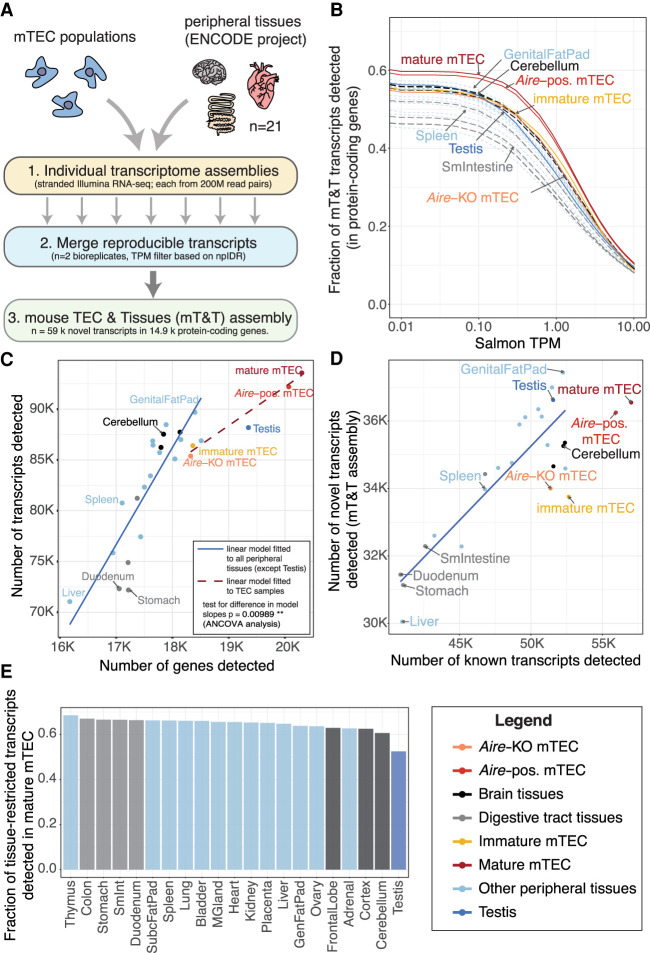
Comparative analysis of transcript expression in mTEC and peripheral tissues. (*A*) Generation of a common mouse mTEC and peripheral Tissues (mT&T) transcriptome assembly. (*B*) Fractions of transcripts from protein-coding genes detected in peripheral tissues and mTEC populations across a range of TPM thresholds. The scatter plots (*C*,*D*) show the relationships between the number of genes and transcripts (*C*) and the number of known versus novel transcripts (*D*) detected in peripheral tissues and mTEC populations. The linear models shown in *C* and *D* were fitted to all samples except TEC and testis (solid trend lines) or to the TEC samples (*C*, dashed trend line). There was a significant difference in the trend line slopes in *C* (*P* = 0.00989, ANCOVA analysis). (*E*) Fractions of sets of tissue-restricted transcripts (*tau* ≥ 0.9) from peripheral tissues detected in mature mTEC (see also Supplemental Fig. 2I). Analyses shown were restricted to protein-coding genes and performed using a single high-depth sample per tissue. Similar results were obtained using lower-depth biologically replicate sample pools (n = 2) (Supplemental Fig. 2C–E,H).

To compare the splicing landscape of TEC with peripheral (i.e., non-thymic) tissues, we performed deep, stranded RNA sequencing of immature and mature mTEC and integrated the data with that from similar sequencing of 21 separate tissues from the mouse ENCODE Project (Pervouchine et al. 2015). To ensure that our investigations were not biased by differential representation of tissue or TEC transcripts in the available reference annotation, we began our analysis by constructing a mouse TEC and Tissue (mT&T) transcriptome assembly. To do so we built a set of reference-guided individual assemblies from each TEC population and peripheral tissue using matched high-depth sequence read pools (200 million reads/pool). To avoid inclusion of assembly artifacts, only transcripts that could be reproducibly detected in biologically replicate sample pools using a refined npIDR approach (Supplemental Fig. 1) were merged into the final mT&T assembly (Fig. 1A). The mT&T assembly included 58,990 novel transcripts in 14,865 protein-coding genes. In agreement with previous reports (Keane et al. 2015; St-Pierre et al. 2015; Danan-Gotthold et al. 2016), we found that mature mTEC expressed a greater number of transcripts from protein-coding genes than any of the surveyed peripheral tissues (60.1%) (Fig. 1B). However, modeling of the relationship between the number of detected genes and transcripts revealed that mature mTEC produced significantly fewer transcripts per gene than was predicted by the relationship between gene and transcript number in the peripheral tissues (Fig. 1C). We confirmed this finding by repeating the analyses using reference Ensembl annotations, biologically replicate sample pools, and independent data sets (Supplemental Fig. 2A–G).

**Figure 2.**
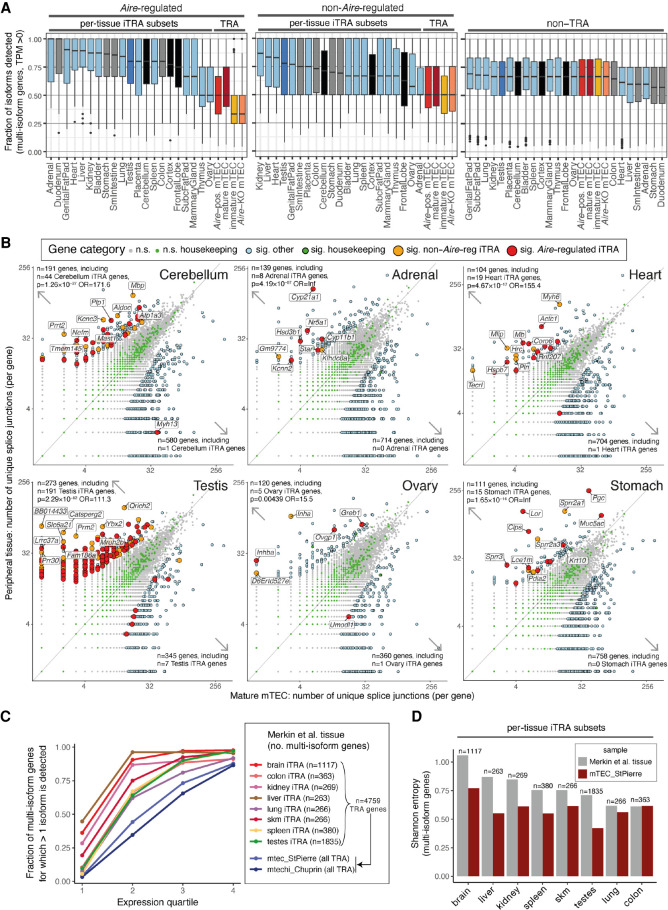
mTEC express fewer isoforms of TRA genes than peripheral tissues. (*A*) The fraction of isoforms detected (TPM > 0) per multi-isoform protein-coding gene in mTEC and ENCODE peripheral tissues. The box plots show the fractions for *Aire*-regulated TRA genes (*left*), non-*Aire* TRA genes (*middle*), and non-TRA genes (*right*, colors as in Fig. 1) (Supplemental Fig. 3D). (*B*) The numbers of splice junctions found in protein-coding genes (points) in mature mTEC (this study; *x*-axes) versus peripheral tissues (ENCODE Project; *y*-axes). Six selected peripheral tissues are shown, with the remaining 14 displayed in Supplemental Figure 5A. Significant (sig.) differences in junction number were identified using edgeR (BH adjusted *P* < 0.05, |fc| > 2). *P*-values and odds ratios (OR) from Fisher's exact tests for enrichment of iTRA genes among the genes with significantly higher junction counts in the peripheral tissues are reported (*top left*). The top 10 of each tissue's iTRA genes with significant differences in junction counts are labeled (as ranked by edgeR *P*-value). (*C*,*D*) Validation with independent peripheral tissue (Merkin et al. 2012) and TEC data sets (St-Pierre et al. 2013; Chuprin et al. 2015). (*C*) The fraction (*y*-axis) of alternatively spliced multi-isoform genes (>1 splice isoform detected) is shown for individual sets of tissue iTRA genes by expression quartile (*x*-axis). (*D*) The bar plots show the mean Shannon entropy (*y*-axis) of splice isoform expression for sets of multi-isoform tissue iTRA genes (*x*-axis). Alternate versions of *C* and *D* made using all genes are shown in Supplemental Figure 5B and 5C. For *A* and *C*, the full sets of TRA genes were quantitated in the TEC populations.

We next sought to determine the extent to which the splicing of promiscuously expressed genes in TEC mirrors the patterns found in the normal tissue context. To do so we first defined three categories of protein-coding genes: (1) n = 3889 *Aire*-regulated “tissue-restricted antigen” (TRA) genes, (2) n = 5266 non-*Aire*-regulated TRA genes, and (3) n = 12,885 non-TRA genes. *Aire-*regulated genes were defined as those found to be significantly down-regulated in *Aire*-knockout mTEC (fc > 2, BH adjusted *P* < 0.05), whereas TRA genes were identified using the *tau* tissue-specificity metric (Supplemental Methods; Supplemental Fig. 3; Supplemental Table 2; Kryuchkova-Mostacci and Robinson-Rechavi 2017). We reasoned that peripheral tissues are more likely to engage in alternative splicing of the TRA genes that contribute to their specialized functions. We therefore also defined a collection of individual-tissue TRA gene subsets, which we term “iTRA,” by assigning each TRA gene to the tissue in which it was most highly expressed. Comparison of the splicing of iTRA subsets in their cognate peripheral tissues with thymic TRA gene splicing revealed that TEC expressed a significantly smaller fraction of TRA gene isoforms than can be found in the relevant peripheral tissues (Fig. 2A; Supplemental Table 13). These observations did not appear to be a consequence of a lower sequencing coverage of promiscuously expressed genes in TEC (Supplemental Fig. 4A–C), because significantly fewer isoforms were detectable in mTEC regardless of gene expression level (Supplemental Fig. 4D–J). Examination of unique splice junction numbers confirmed that genes more extensively spliced in individual peripheral tissues than in mature mTEC were strongly and significantly overrepresented for the cognate iTRA subsets (19 of 20 tissues, Fisher's exact test) (Fig. 2B; Supplemental Fig. 5A). For example, in the cerebellum we found n = 44 cerebellum iTRA genes with higher splice junction counts and only n = 1 such gene with lower numbers of splice junctions (*P* = 1.26 × 10^−27^, odds ratio = 171.6). In this tissue we noted that *Plp1*, for which incomplete thymic splicing can contribute to experimental autoimmune encephalomyelitis (EAE), had significantly higher numbers of splice junctions in the periphery (Anderson et al. 2000; Klein et al. 2000). Additionally, we noted that several homologs of genes linked to human autoimmune disease had significantly higher numbers of splice junctions in the peripheral tissues than was found in mature mTEC. These included myelin basic protein, *Mbp* (n = 112 junctions in cerebellum, n = 17 junctions in mature mTEC, FDR = 4.066 × 10^−15^), which is a target of autoantibodies in multiple sclerosis (Berger et al. 2003), and *Cyp21a1* (n = 140 junctions in adrenal tissue, n = 5 junctions in mature mTEC, FDR = 1.76 × 10^−21^) the homolog of the human *CYP21A2* gene that encodes cytochrome P450 family 21 subfamily A member 2 (also known as P450c21B), a major target of autoantibodies in Addison's disease (Winqvist et al. 1992). Because previous genomic investigations suggested more promiscuous and extensive programs of splicing in mTEC (Keane et al. 2015; Danan-Gotthold et al. 2016), we sought to verify our observations using the data from those studies. We began by reproducing the original observations of unusually high overall transcriptome complexity in mTEC (Supplemental Fig. 5B,C). However, analysis of these independent data sets with the iTRA approach confirmed that mTEC engage in less alternative splicing of iTRA than can be found in the cognate peripheral tissues (Fig. 2C,D).

As previously reported (Keane et al. 2015; Danan-Gotthold et al. 2016), we found that the mature mTEC population coexpressed transcript isoforms that normally arise in distinct anatomical locations (Supplemental Fig. 6A–C). In addition, we found evidence that such transcripts can be produced by the same cell: single-cell RNA sequencing data revealed that the thyroid and nervous-system specific transcript isoforms of *Calca* were often produced together in individual mTEC (Supplemental Fig. 6D). We also assessed the representation of sets of tissue-restricted isoforms from peripheral tissues in mTEC. Transcripts with testis-restricted expression were most markedly underrepresented in mTEC (52.5% detected), followed by those from the brain (60.7–62.9%), adrenal (62.7%), and ovary (63.6%) (Fig. 1E, with results from biological replicate sample pools shown in Supplemental Fig. 2H).

Finally, although mature mTEC expressed a higher number of known transcripts (n = 57,019) than was observed in peripheral tissues, we found that they produced a relatively low number of novel transcripts (n = 36,547) (Fig. 1D). Together our results show that although TEC produce an atypically high absolute number of transcripts, they produce significantly fewer isoforms of TRA genes than can be found in the relevant peripheral tissues.

### Genes harboring novel transcripts in mTEC are associated with T cell selection

**Figure 3.**
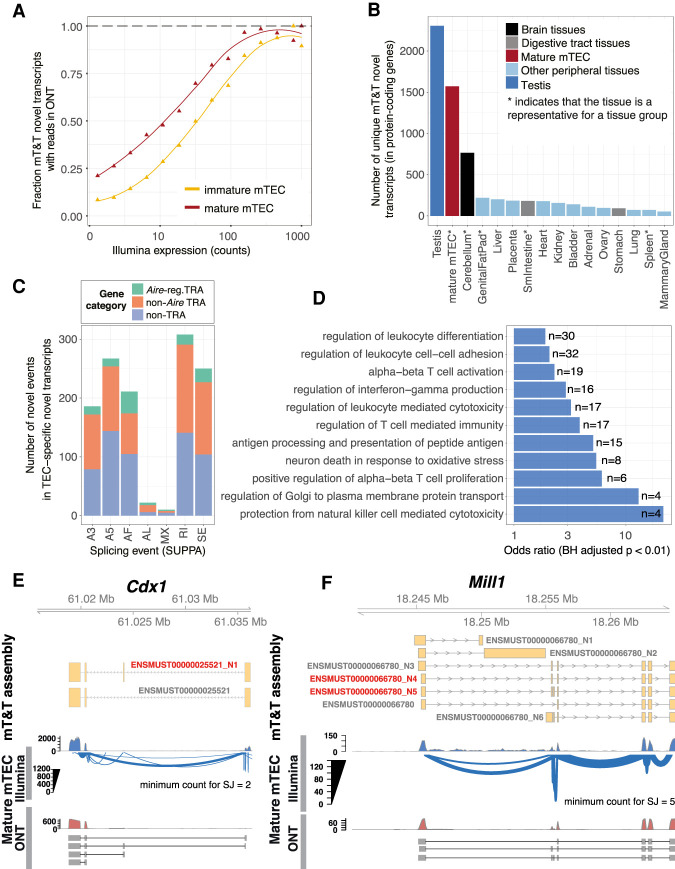
Identification and characterization of TEC-specific novel transcripts. (*A*) Validation of novel mT&T transcripts using ONT RNA sequencing. The fraction of novel transcripts supported by ONT reads are shown for mature mTEC (red) and immature mTEC (yellow). (*B*) Number of novel transcripts “uniquely” detected in mature mTEC and representative peripheral tissue samples (Supplemental Methods; Supplemental Figs. 3A, 8). (*C*) TEC-specific novel splicing events by event type and promiscuous expression status. Individual events may be counted in multiple categories. (SE) skipped exon; (RI) retained intron; (MX) mutually exclusive exon; (A3/A5) alternative 3′/5′ splice site; (AF/AL) alternative first/last exon. (*D*) Selected GO biological processes significantly overrepresented in the set of genes (n = 1167) from which the mTEC-specific novel transcripts were derived (one-sided Fisher's exact tests; BH adjusted *P* < 0.01). (*E*,*F*) *Cdx1* and *Mill1* are displayed as examples to show novel TEC-specific transcripts (red). Existence of the novel transcripts in mature mTEC was supported by both Illumina (Sashimi plots) and long-read ONT (selected reads) data. Novel transcripts are indicated by the “_N” suffix.

Alternative splicing events are often associated with the evolution and modification of protein function (Kelemen et al. 2013). Because the transcriptome of TEC is relatively understudied, we reasoned that novel splicing events specific to TEC might be associated with their specialized functions for T cell selection. Using long-read Oxford Nanopore Technologies (ONT) sequencing (Supplemental Fig. 7), we validated the existence 64.3% of the mT&T novel transcript structures that were expressed at moderate or high levels in mature mTEC (>10 counts) (Fig. 3A). Next, we identified novel transcripts that showed TEC or tissue-specific expression. For this analysis we chose a single tissue to represent each group of related tissues (Supplemental Fig. 3A). We found that mature mTEC expressed a higher number of unique novel transcripts than did brain tissue (see also Supplemental Fig. 8), but substantially fewer than were found in the testis (Fig. 3B; Supplemental Table 3). The majority (85%) of the 1572 novel transcripts uniquely expressed in mature mTEC were found for loci that do not require *Aire* for their expression (Fig. 3C).

The 1167 protein-coding genes that harbored TEC-specific novel transcripts displayed significant enrichments for Gene Ontology (GO) biological processes such as “regulation of leukocyte differentiation,” “regulation of T cell–mediated immunity,” and “antigen processing and presentation of peptide antigen” (Benjamini–Hochberg [BH] adjusted *P* < 0.01, one-sided Fisher's exact tests) (Fig. 3D; Supplemental Table 4), suggesting that they are likely to encode for nonpromiscuously expressed factors that have a functional role in T cell selection in TEC. Novel transcript structures were detected in genes of known importance for TEC function, including *Foxn1* (Žuklys et al. 2016) and *Aire* (Supplemental Table 3; Abramson and Goldfarb 2016). Genes that produced transcripts harboring novel exons or exon skipping events in TEC included *Cdx1*, a transcription factor linked with mTEC maturation (Fig. 3E; Handel et al. 2018); *Cd80*, a receptor important for interaction with T cells via CD28 and CTLA4; the MHC class II gene *H2-Aa*; *Mill1*, an MHC class I-like molecule that is known to be expressed on a subpopulation of TEC (Fig. 3F; Kajikawa et al. 2006); as well as *Skint* gene family members, including *Skint2*, which has been reported to be a novel negative T cell regulator (Yang et al. 2007).

Together these observations suggest that the novel transcript structures detected in thymic epithelial cells may have relevance for TEC function.

### *Aire* contributes to alternative splicing and promotes expression of long transcripts

**Figure 4.**
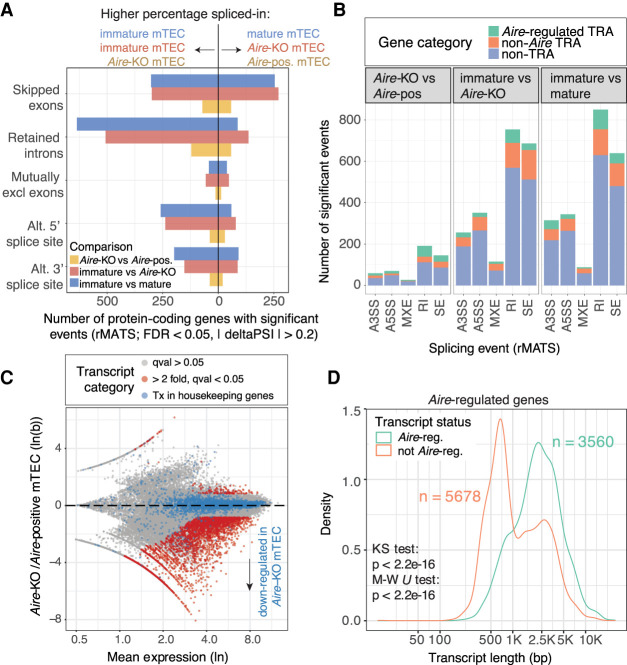
*Aire* promotes the generation of long transcripts in TEC. (*A*) The numbers of protein-coding genes (*x*-axis) in which significant differential splicing events (*y*-axis) were detected; comparisons of immature versus mature mTEC (blue), immature mTEC versus *Aire*-knockout mTEC (red) and *Aire*-knockout versus *Aire*-positive mature mTEC (yellow) (n = 2 replicates per sample). (*B*) Breakdown of identified splicing events by event type and promiscuous expression status. (SE) skipped exon; (RI) retained intron; (MXE) mutually exclusive exon; (A3SS/A5SS) alternative 3′/5′ splice site. (*C*) MA plot of differential transcript expression in *Aire*-knockout compared to *Aire*-positive mTEC. Transcripts regulated by *Aire* are shown in red (sleuth, Wald test, *Q*val < 0.05, fc ≳ 2, n = 2 replicates per sample). Transcripts from housekeeping genes are shown in blue. (*D*) The length distributions of *Aire*-regulated and non-*Aire*-regulated transcripts in *Aire*-regulated genes (analysis limited to genes that contained at least one significantly *Aire*-regulated transcript as defined in *C*).

To clarify the role of Aire in alternative splicing in TEC, we also generated deep and stranded Illumina sequencing data from mature *Aire*-positive and mature *Aire*-knockout mTEC. We discovered 492 significant (5% FDR, rMATS) *Aire*-regulated alternative splicing events in transcripts from 459 protein-coding genes (Fig. 4A; Supplemental Tables 5, 14). There was, however, a much larger difference in transcript splicing between immature and mature wild-type mTEC (n = 2236 events in n = 1967 protein-coding genes, 5% FDR, rMATS analysis) (Fig. 4A; Supplemental Table 5). To confirm that these differences were not linked to the much higher expression of *Aire* in mature mTEC (Supplemental Fig. 9B), we performed an analysis of the differences in splicing events between immature wild-type and mature *Aire*-knockout mTEC. As expected, we found a similar number of differences to that identified for the wild-type comparison, demonstrating that the extensive splicing differences between immature and mature mTEC are controlled by *Aire*-independent factors (Fig. 4A). The majority of the *Aire*-controlled and TEC maturity-related alternative splicing events were found in transcripts encoding non-TRA genes (Fig. 4B). In addition, a large number of retained introns were found in the transcripts of immature mTEC (n = 760) (Fig. 4A). Coordinated changes in intron retention during cellular differentiation are not unusual, and transcripts harboring retained introns can arise from genes that have specialized cellular functions (Jacob and Smith 2017). Overall, however, we found that immature and mature TEC had fewer retained introns than did peripheral tissues (Supplemental Fig. 9A).

AIRE is known to promote expression of distal exons (Meredith et al. 2015) and the release of stalled polymerases (Giraud et al. 2012). We therefore investigated whether *Aire* might favor the production of long transcripts. Differential transcript usage analysis (Fig. 4C) revealed that *Aire* positively regulated the generation of a large number of transcripts (n = 4027, BH adjusted *P* < 0.05, fold change ≳ 2) (Supplemental Table 6). We found that *Aire*-regulated transcripts arising from *Aire*-regulated genes had a significantly different length distribution (Kolmogorov–Smirnov test, *P* = 2.2 × 10^−16^) and location (Wilcoxon test, *P* = 2.2 × 10^−16^) being on average >1 kb longer than their non-*Aire*-regulated counterparts (Fig. 4D). Analysis of the structures of these transcripts showed that although most (85.3%) of the *Aire*-regulated transcripts comprised “classical” transcripts with both 5′ and 3′ UTRs, the majority (68.1%) of non-*Aire-*regulated transcripts had nonclassical or unannotated structures (Supplemental Fig. 9C–F). Overall, we found that *Aire*-regulated transcripts had significantly shorter 5′ UTRs (−15.5 bp; −7.6%), longer CDSs (+460.8 bp; +38.6%), and longer 3′ UTRs (+235.4 bp; 28.4%) (Supplemental Fig. 9G). These findings show that *Aire* plays a major role in promoting the production of long “classical” transcripts (with 5′ and 3′ UTRs) from its target loci while only having a limited impact on the generation of alternative splicing events. Hence, splicing factors other than *Aire* must be primarily responsible for the alternative splicing of transcripts in mature mTEC.

### Medullary TEC express a distinct set of SFs that includes *Rbfox1*

**Figure 5.**
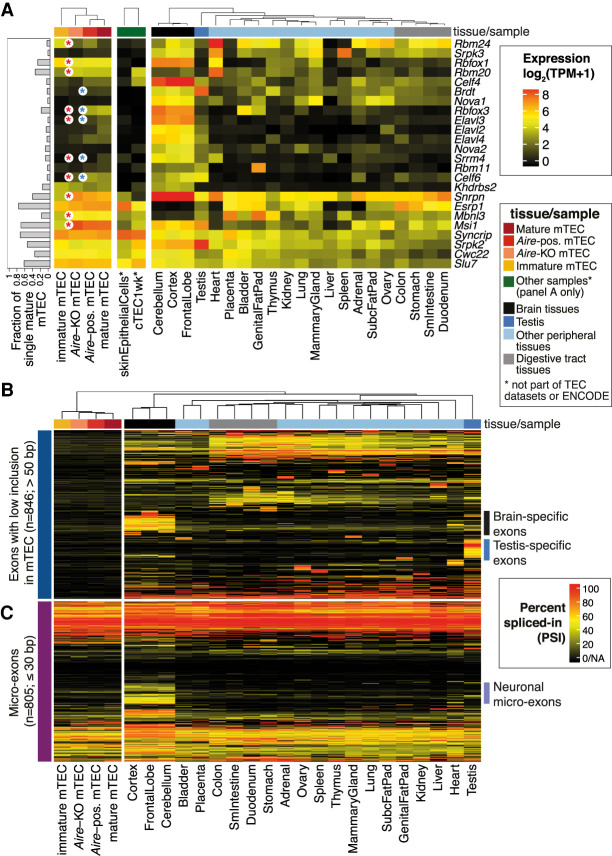
Selective expression of peripheral SFs and exons in mTEC. (*A*) Expression of a set of tissue-restricted (*tau* > 0.5) genes that encode for bona fide SFs (Supplemental Table 8) in mTEC populations, skin epithelia, cTEC, and peripheral tissues (mouse ENCODE Project). The bars (*left*) depict the fraction of a set of single mature mTEC that express each factor. Significant differences in expression between immature versus mature (*Aire*-KO) mTEC or *Aire*-KO versus *Aire*-positive mTEC are indicated by red and blue asterisks, respectively (BH adjusted *P*-value < 0.05, |fc| > 2, DESeq2 analysis of the population RNA sequencing data, n = 2 biological replicates/condition). (*B*) Patterns of protein-coding exon (>50 bp in length) inclusion for exons with a low inclusion rate in mTEC (mean PSI < 0.1; max PSI < 0.2) that were included in at least one of the peripheral tissues (PSI > 0.5). (*C*) Microexon (≤30 bp) inclusion in transcripts from protein-coding genes in the mTEC population and peripheral tissue samples (Supplemental Fig. 11B).

The ability of mTEC to express a large number of peripheral isoforms suggested that they might reuse tissue-specific SFs. After first noting that expression of core spliceosome genes was intact in mTEC (Supplemental Fig. 10A), we systematically identified a set of 146 splicing-related genes that showed tissue-restricted expression (Supplemental Methods; Supplemental Figs. 10B, 11A; Supplemental Table 7). Based on literature searches we further narrowed our focus to n = 24 tissue-restricted splicing factors (TRSFs) with known roles in controlling alternative splicing (Fig. 5A; Supplemental Table 8). Of these we noted the frequent (detected in >20% of single mTEC) and nonpromiscuous expression of *Rbfox1*, *Rbm20*, and *Msi1* in mature mTEC. These factors also showed little, if any, expression in skin epithelia (Fig. 5A), suggesting that they may be relevant to the specialized function of mTEC. RBFOX1 and RBM20 control alternative splicing in the brain and myocardium (Guo et al. 2012; Conboy 2017), whereas MSI1 is an RNA-binding protein implicated in regulating splicing in photoreceptors (Murphy et al. 2016). Meanwhile, we noted that the majority of the 24 TSRFs—including those restricted in their expression to the brain (such as *Nova1*, *Nova2, Elav2, Elav3, Elav4*, and *Rbfox3*), testis (*Brdt*), and heart (*Rbm24*)—were expressed at low frequency or showed only promiscuous expression in mature mTEC (Fig. 5A).

In line with the absence of neural and testis-specific SFs, sets of coding exons frequently included in transcripts detected in the brain and testis were found to be excluded from mTEC mRNA (PSI < 0.1) (Fig. 5B). In particular, we noted that brain-specific microexons (≤30 bp) were rarely included in mTEC transcripts (Fig. 5C), with the PSI distributions of these microexons in TEC being very similar to that found in other non-neuronal tissues (Supplemental Fig. 11B). This observation may be explained by the low expression of *Srrm4* in mTEC (Fig. 5A), because this factor is known to be responsible for promoting the inclusion of neuronal microexons (Quesnel-Vallières et al. 2015, 2016). In summary we found that mTEC constitutively express only a small number of peripheral SFs. Together with the clear absence of a set of neuronal microexons, these results suggest that mTEC may have evolved to selectively represent specific facets of the peripheral tissue isoform repertoire.

### RBFOX is present with AIRE in mTEC nuclei and influences TEC development

**Figure 6.**
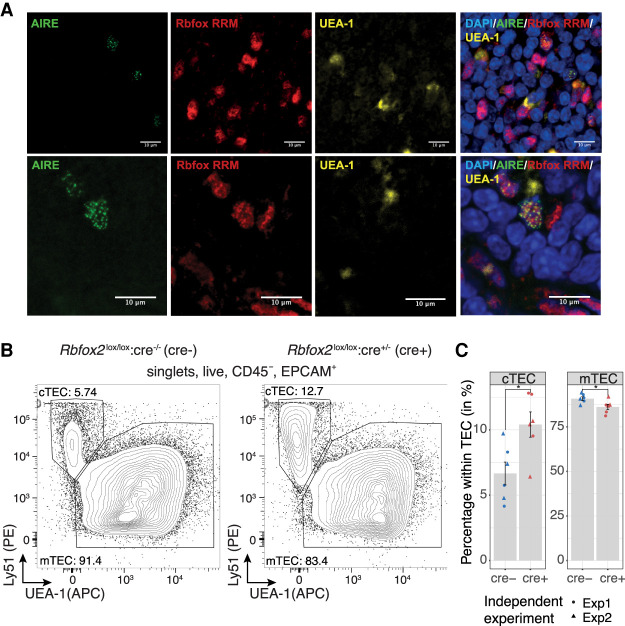
RBFOX is present with AIRE in mTEC nuclei and influences TEC development. (*A*) Confocal immunofluorescence analysis of the localization of AIRE (green) and RBFOX (red) in the thymic medulla. RBFOX was detected using an anti-RRM domain-specific antibody, mTEC were identified by reactivity with the lectin UEA-1 (yellow), and nuclei were labeled using DAPI (blue). Two representative sections are shown in the *upper* and *lower* panels. (*B*) Representative flow cytometric analysis of cTEC and mTEC frequencies among thymic epithelial cells extracted from *Rbfox2* tKO animals. (*C*) Quantification of cTEC and mTEC frequency in *Rbfox2* tKO animals. The mean ± SE from n = 2 independent experiments is shown in bar graphs; (*) *P*-value < 0.05 for two-sided Welch two sample *t*-test.

Among the TRSFs expressed by mTEC, *Rbfox1* was of particular interest owing to the established roles of RBFOX in controlling alternative splicing during muscle and brain development (Conboy 2017). We noted that *Rbfox1* and *Rbfox2* transcripts in mTEC predominantly included the neuronal B40 exon and excluded the muscle-specific M43 exon (Supplemental Fig. 12A,B; Nakahata and Kawamoto 2005; Damianov and Black 2010). Confocal image analysis of AIRE-expressing mTEC with a pan RBFOX anti-RRM domain antibody revealed that RBFOX factors are present in the nucleus in proximity to AIRE speckles (Fig. 6A). Although this observation does not imply colocalization, it does provide evidence that RBFOX factors are coexpressed with AIRE and hence may be important for the splicing of *Aire-*regulated transcripts.

Both *Rbfox1* and *Rbfox2* showed a robust nonpromiscuous and tissue-restricted expression in mTEC (Fig. 5A; Supplemental Fig. 12C,D). To investigate their functions in mTEC, we generated conditional knockouts (Gehman et al. 2011, 2012) using the expression of Cre recombinase under the transcriptional control of the *Foxn1* locus (Žuklys et al. 2009). Total thymus cellularity of *Rbfox1* thymus knockout (tKO) (*Rbfox1*^*lox*^^/lox^:*Foxn1*^*cre*^^/+^) mice was 15% increased, whereas that of age-matched *Rbfox2* tKO (*Rbfox2^lox/lox^:Foxn1^cre/+^*) mice remained unchanged when compared to controls (Supplemental Figs. 13C, 14C). However, the phenotype of cTEC, mTEC, immature mTEC, and mature mTEC was unaffected in *Rbfox1* tKO mice (marker phenotypes in Supplemental Table 9; Supplemental Fig. 13). In contrast, *Rbfox2* tKO mice showed an overall reduction in TEC frequency (CD45^−^ EPCAM^+^, *Rbfox2* tKO 0.12% vs. WT 0.17% of all thymic cells recovered, *P* < 0.05, two-sided Welch two sample *t*-test) (Supplemental Fig. 14D,E). The *Rbfox2* tKO mice also had proportionally more cTEC (10.4% vs. 6.7% of all TEC, *P* = 0.02, Welch two sample *t*-test) and proportionally fewer mTEC (86.2% vs. 90.8% of all TEC, *P* = 0.03, Welch two sample *t*-test) present in their epithelial scaffolds (Fig. 6B,C). *Rbfox1/2* tKO (*Rbfox1^lox/lox^*: *Rbfox2^lox/lox^*:*Foxn1^cre/+^*) animals showed changes in cTEC and mTEC frequency that were quantitatively similar to that of *Rbfox2* tKO mice (Supplemental Fig. 15) suggesting that, as in other tissues (Gehman et al. 2011), *Rbfox2* can compensate for the loss of *Rbfox1* in TEC or that *Rbfox1* might not play a role in this aspect of TEC development. Thymocyte development and positive selection were quantitatively normal in the *Rbfox1/2* tKO animals (Supplemental Fig. 16). In summary these data show that *Rbfox1* contributes to the regulation of thymic cellularity, and *Rbfox2* increases the relative frequency of mTEC and decreases the relative frequency of cTEC within the TEC compartment.

### RBFOX contributes to the alternative splicing of self-antigen transcripts in mTEC

To investigate the impact of RBFOX on the mTEC transcriptome we performed RNA sequencing of immature and mature mTEC isolated from *Rbfox1* tKO, *Rbfox2* tKO, and control mice. In mature mTEC, loss of *Rbfox1* or *Rbfox2* had no obvious effect on the expression of known maturation or subpopulation markers (Supplemental Fig. 17) and induced only minor changes in gene expression (Supplemental Fig. 18A) but caused hundreds of alterations in alternative splicing (*Rbfox1* tKO: n = 559 events in 535 genes; *Rbfox2* tKO: n = 668 events in 624 genes; FDR < 0.05, |delta PSI| > 0.2) (Supplemental Fig. 18; Supplemental Tables 10, 11). In mature mTEC, loss of *Rbfox1* and *Rbfox2* resulted in n = 123 significant alternative splicing events in 104 TRA genes and n = 122 events in 110 TRA genes, respectively. In immature mTEC, loss of *Rbfox1* and *Rbfox2* resulted in the alternative splicing of a smaller number of TRA genes (72 events in 51 TRA genes and 43 events in 36 TRA genes, respectively) in keeping with the lower level of promiscuous gene expression in these cells (Supplemental Fig. 19A–C). Analysis of the overlap of the sets of genes alternatively spliced in *Rbfox1* and *Rbfox2* tKO mice revealed that the splicing of 10 TRA genes was affected by both factors in mature mTEC (odds ratio [OR] = 4.64, *P* = 1.71 × 10^−4^, two-sided Fisher's exact test), whereas the splicing of six TRA genes was affected by both factors in immature mTEC (OR = 18.5, *P* = 3.23 × 10^−6^, two-sided Fisher's exact test) (Supplemental Fig. 19D). We did not find an obvious overlap between the splicing events identified in the *Rbfox1* and *Rbfox2* tKO mTEC and those found in *Aire*-knockout mTEC or that were associated with mTEC maturation (Supplemental Fig. 20).

**Figure 7.**
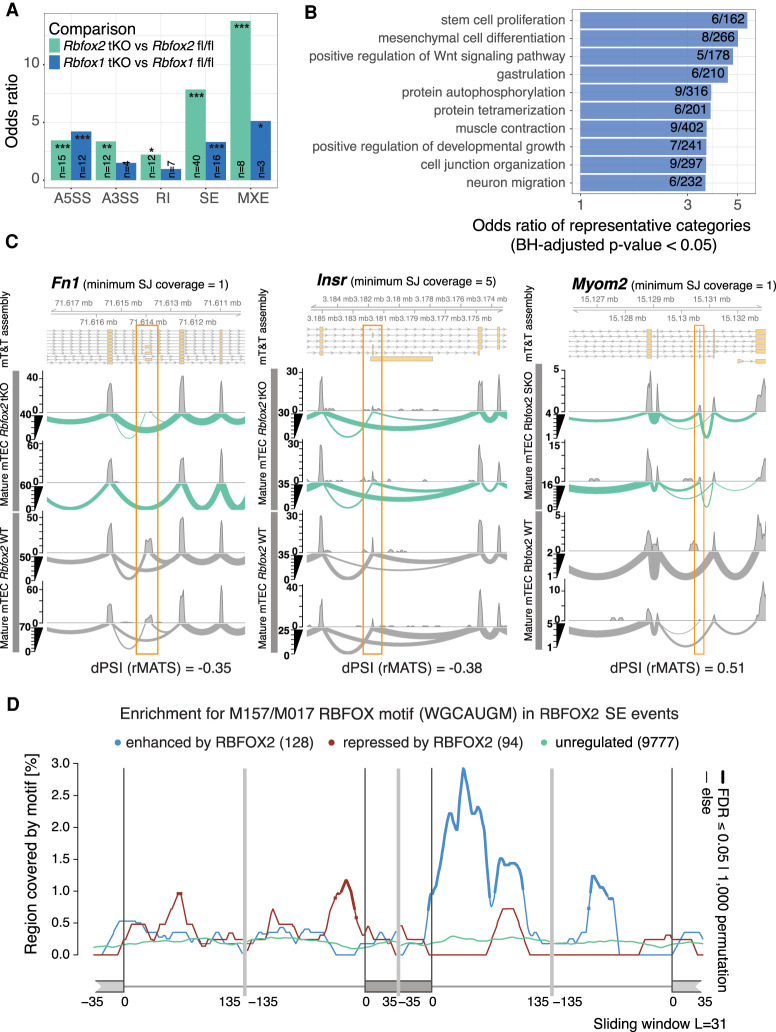
RBFOX regulates alternative splicing in TEC. (*A*) The bar plots show the enrichments (ORs) of previously predicted Rbfox target genes (Weyn-Vanhentenryck et al. 2014) in the sets of genes differentially spliced in the *Rbfox1* tKO or *Rbfox2* tKO mature mTEC (two-sided Fisher's exact tests, BH adjusted *P*-values): (*) *P* < 0.05, (**) *P* < 0.01, (***) *P* < 0.001; abbreviations as for Figure 4B. (*B*) Selected GO biological processes significantly overrepresented (one-sided Fisher's exact tests, BH adjusted *P*-values < 0.05) in genes that contained significantly RBFOX2-regulated SE events in mature mTEC. (*C*) Three examples of significantly (FDR < 0.05) RBFOX2-regulated splicing events in mature mTEC. *Fn1* and *Insr* are known RBFOX target genes (Chen and Manley 2009; Weyn-Vanhentenryck et al. 2014). *Myom2* is an example of a non-*Aire* TRA gene. (*D*) Enrichment of the RBFOX recognition motif (M159/M017) (Ray et al. 2013) in the sequences surrounding exons that were found to be significantly regulated by RBFOX2 in mature mTEC (Supplemental Fig. 18B). The lines show enrichments for sets of exons found to be enhanced (blue), repressed (red), or not significantly regulated (green) by RBFOX2. Thicker lines indicate regions of statistically significant enrichment (FDR ≤ 0.05, n = 1000 permutations).

We next sought to establish whether the genes that showed modified splicing patterns in the *Rbfox1* and *Rbfox2* tKO mature mTEC included those previously predicted to be RBFOX targets in mouse brain (Weyn-Vanhentenryck et al. 2014) and muscle tissues (Pedrotti et al. 2015; Singh et al. 2018). In mature mTEC, we identified large and significant overlaps between predicted RBFOX brain target genes and those for which loss of *Rbfox2* induced (1) exon skipping in mTEC (OR = 7.8, BH adjusted *P* = 5.4 × 10^−19^) or (2) the use of mutually exclusive exons (OR = 13.8, BH adjusted *P* = 1.4 × 10^−5^) in mTEC (Fig. 7A). Similarly large and significant overlaps were identified for the RBFOX muscle target genes (Supplemental Fig. 18D). In keeping with this observation, many of the TRA genes alternatively spliced upon loss of *Rbfox2* showed specific expression in neuronal tissues (Supplemental Fig. 18E). GO biological processes overrepresented among the genes alternatively spliced upon loss of *Rbfox2* in mature mTEC included “muscle contraction” and “neuron migration” in line with the known roles of *Rbfox* gene family members in muscle and neuronal tissues (Fig. 7B; Conboy 2017). Examples of genes harboring alternative splicing events in the *Rbfox2* tKO mature mTEC included *Fn1* and *Insr*, two known RBFOX2 target genes (Chen and Manley 2009; Weyn-Vanhentenryck et al. 2014) as well as the TRA *Myom2* (Fig. 7C).

Previous studies have identified an enriched RBFOX recognition motif in proximity to RBFOX-regulated exons (Weyn-Vanhentenryck et al. 2014). We observed a significant enrichment of the conserved RBFOX RRM WGCAUGM motif (Ray et al. 2013) upstream of exons repressed and downstream from exons enhanced by the presence of *Rbfox2* in both mature and immature mTEC (Fig. 7D; Supplemental Fig. 19E), following the patterns previously described for this factor (Weyn-Vanhentenryck et al. 2014). In summary these data provide evidence that RBFOX SFs directly regulate the splicing of both promiscuously and nonpromiscuously expressed genes in mature mTEC.

## Discussion

Our work shows that although mature mTEC produce an exceptionally high proportion (∼60%) of peripheral splice variants, they are unable to recreate the full diversity of TRA gene isoforms that are present in the periphery. The concept of selective representation of peripheral isoforms in TEC is supported by a recent qPCR-based study that estimated that a quarter of the genes studied contained epitopes not expressed in the thymus (Shilov et al. 2019). Using our refined iTRA approach, we noticed that genes that have far fewer splice junctions in murine mTEC included those encoding homologs of known autoantibody targets in human autoimmune disease (such as PLP1, MBP, and CYP21A2). Although confirmation is needed by analysis of human mTEC, these findings suggest, as is understood to be the case in a mouse model of EAE, that absence of TRA isoforms from the thymus may lead to an incomplete central screening of peripheral tissue epitopes and thus contribute to the development human autoimmune disease (Anderson et al. 2000; Klein et al. 2000). We found for TRA genes that very substantive numbers of splice junctions were absent in TEC, but although we consider it to be unlikely, we cannot formally exclude the possibility that peptides from non-thymically expressed splice isoforms may be redundant or excluded from the peripheral MHC peptidome. Overall, our rigorous transcriptome assembly–based census of transcript structures showed an even representation of tissue-restricted transcripts from the 21 surveyed peripheral tissues in mTEC. Most notably we found a lower representation of transcripts from the testis and brain together with a corresponding absence (or only very weak) expression of brain and testis-specific SFs in mTEC. It is possible that it may be functionally less important to educate T cells against splice isoform epitopes specific to these tissues, which constitute so-called immune privileged sites. Further, the unusually high complexity of splicing in the testes and brain suggests the possibility that immune surveillance may act as a constraint on the evolution of splicing complexity in non-immune privileged peripheral tissues. Our finding that neuronal microexons are not frequently spliced into transcripts in mTEC is likely explained by the absence of the microexon SF *Srrm4* in these cells (Quesnel-Vallières et al. 2015). The limited inclusion of neuronal microexons in mTEC provides a possible link between the observations that such exons are misregulated in the brains of patients with autism (Irimia et al. 2014) and growing evidence that autoimmunity may be involved in autism (Hughes et al. 2018). Aside from the testis and cerebellum, adrenal and liver tissues showed a weaker isoform representation in mature TEC. In humans, these tissues are known sites of autoimmunity: autoimmune adrenalitis is the most common cause of Addison's disease, and liver diseases such as autoimmune hepatitis, primary biliary cirrhosis, and sclerosing cholangitis are all thought to be the consequence of autoimmunity (Decock et al. 2009). Alternative splicing has been implicated in such diseases (Webster 2017), and our data suggest that lack of thymic representation of isoforms specific to these tissues might contribute to a higher susceptibility for autoimmunity.

Our investigations revealed a large number of splicing differences between immature and mature mTEC. One possible explanation for this observation is the expression of AIRE in mature mTEC, but our analysis of *Aire*-knockout mTEC confirms that this molecule plays only a small role in alternative splicing in mTEC. Rather, we found that *Aire* promotes the use of longer “classical” transcripts containing both 5′ and 3′ UTRs in agreement with and extending the existing understanding that AIRE releases stalled RNA polymerases that otherwise produce “short” transcripts (Giraud et al. 2012). A comprehensive survey of splicing factor expression revealed that mTEC complement typical epithelial splicing factors such as *Esrp1/2* with a small number of peripheral restricted splicing factors. Unlike skin epithelia, mature mTEC showed nonpromiscuous expression of *Rbm20*, *Msi1*, and *Rbfox1*. This observation, along with the apparently incomplete representation of peripheral structures and absence of excessive numbers of novel splice isoforms in mTEC, suggests that TEC undertake a selective program of alternative splicing to ensure the accurate representation of a subset of the peripheral splice isoform repertoire. We hence conclude that transcript structures in mTEC are primarily shaped by a small number of splicing and mRNA processing factors (Guyon et al. 2020) and that mTEC do not rely on “promiscuous” mechanisms to promote and increase splice isoform diversity, as previously suggested (Keane et al. 2015). Rather (and assuming that our observations hold in humans), a limited thymic representation of peripheral splice isoforms is consistent with the concept that tissue-specific isoforms are relevant sources of autoantigens in immune-mediated diseases (Evsyukova et al. 2010; Juan-Mateu et al. 2016; Newman et al. 2017). Furthermore, characterization of transcript structures in human thymic epithelial cells would be expected to aid the identification of autoantigen transcripts encoding epitopes against which central tolerance has not been achieved (Ng et al. 2004). Knowledge of such epitopes is of great value for development of antigen-specific therapies for autoimmune disease such as those based on the use of tolerizing peptides or tolerogenic dendritic cells (Pozsgay et al. 2017; Mosanya and Isaacs 2019).

Our discovery of nonpromiscuous *Rbfox1* expression in TEC was of particular interest because this factor is otherwise restricted to muscle and neural tissues in which it plays important developmental roles (Conboy 2017). We detected *Rbfox1* in medullary but not cortical TEC, suggesting that it may be important for the development or function of this subpopulation. We therefore performed functional analysis of the role of *Rbfox1* and its homolog *Rbfox2* in mTEC, excluding *Rbfox3* from our investigations because it showed only weak and promiscuous expression in these cells. Phenotypic analysis of TEC in animals with a selective loss of one or both of these factors showed that *Rbfox2* increases mTEC frequency and decreases cTEC frequency within the TEC population. Recently, it has been shown that although in the embryonic and newborn thymus, cortical and medullary TEC arise from a common bipotent progenitors, in the adult mouse, mTEC are mostly replenished by lineage-restricted cells (Ohigashi et al. 2015). Our data suggest that members of the *Rbfox* gene family might influence the cell fate choices of the bipotent progenitors or act later to promote the differentiation within the mTEC lineage, but other possibilities cannot be excluded.

In mature mTEC we found that RBFOX factors shape the splicing of both promiscuously and nonpromiscuously expressed genes. It is possible that RBFOX-mediated splicing of promiscuously expressed genes may involve a molecular interaction with AIRE because we found them to be coexpressed in mTEC nuclei, and both proteins are known to partner with DDX5 (Abramson et al. 2010; Damianov et al. 2016). The larger role identified for *Rbfox2* in splicing in mTEC was not unexpected, because in the cerebellum it is known that *Rbfox2* can largely compensate for loss of *Rbfox1* but *Rbfox1* is less well able to ameliorate an absence of *Rbfox2* (Gehman et al. 2012). In addition, the changes in alternative splicing identified following loss of *Rbfox2* may involve *Rbfox1* because *Rbfox1* was itself differentially spliced in the absence of *Rbfox2*. We found examples of both *Aire*-dependent and *Aire*-independent TRA that were alternatively spliced by *Rbfox2* in mTEC. Given the weak population-level expression of TRA in mTEC and the relatively small amount of biological material sequenced for this analysis, we expect the actual number of TRA regulated by RBFOX factors in mTEC to be substantially higher than is reported here. Ultimately, phenotypic investigation of TEC-specific *Rbfox* knockout animals will be important to determine the extent to which their contribution to the splicing of TRA in mTEC is functionally important for the establishment of central tolerance. In summary, our data show that mTEC reuse a small set of peripheral SFs that includes RBFOX to selectively reproduce a broad but ultimately limited subset of the peripheral splice isoform repertoire.

## Methods

### Mice

Wild-type C57BL/6 mice were obtained from Harlan Laboratories or Janvier and maintained as a laboratory in-house colony. *Aire^GFP/+^* mice were previously described (Sansom et al. 2014). *Rbfox1* and *Rbfox2* mutant mice (Gehman et al. 2011, 2012) were maintained on a mixed 129S2/Sv x C57BL/6J background. All animals were kept under specific pathogen-free conditions, and work performed was covered by a UK Home Office Project Licence (G.A.H.).

### Isolation, sorting, and immunostaining of thymic epithelial cells

TEC were isolated from multiple thymi and sorted by surface phenotype (Supplemental Tables 9, 12; Supplemental Methods; Supplemental Fig. 21; Dhalla et al. 2020). Sections were prepared and stained for confocal microscopy as described in the Supplemental Methods.

### RNA sequencing data

For analysis of immature, mature, and *Aire*-knockout mTEC poly(A)^+^ RNA-seq libraries were prepared from cells pooled from multiple female mice (1 μg of total RNA; 4 wk old; n = 2 biological replicates) (Supplemental Table 1) and subjected to 101 bp paired-end stranded Illumina RNA-seq. For analysis of *Rbfox1* tKO and *Rbfox2* tKO animals, immature and mature mTEC were isolated from individual mice and sex-matched littermate controls (10,000–15,000 cells per animal, n = 2 biological replicates, 4 wk old). Stranded RNA-seq libraries were prepared (NEB) and subjected to 150 bp paired-end Illumina sequencing.

For long-read sequencing, CD80^+^86^+^ or CD80^−^86^−^ mTEC were obtained from 4- to 6-wk-old female wild-type mice and processed as above. Details of the ONT sequencing can be found in the Supplemental Methods.

### Computational methods

To construct the mT&T assembly, reads were mapped with HISAT2 (Kim et al. 2019), and reference-guided assembly was performed with StringTie (Genome assembly GRCm38.p5, Ensembl release 91 annotations) (Pertea et al. 2015). To identify reproducibly detected transcripts, we used a modified npIDR approach (Supplemental Fig. 1; Dobin et al. 2013; Pervouchine et al. 2015). ONT reads were mapped with minimap2 (Li 2018). We quantified expression level with Salmon (TPMs) or featureCounts (counts) for the Illumina data sets and with featureCounts for the ONT data sets (Liao et al. 2014; Patro et al. 2017). Splicing analyses were performed using SJCounts (Pervouchine et al. 2013), rMATS (for identification of differential events) (Shen et al. 2014), and SUPPA (for computation of PSI values and transcript annotation) (Alamancos et al. 2015). Differential expression analyses were performed using DESeq2 (genes) (Love et al. 2014), edgeR (splice junctions) (Robinson et al. 2010), or kallisto/sleuth (transcript usage) (Bray et al. 2016; Pimentel et al. 2017). For each analysis, reads were preprocessed, trimmed, de-duplicated, and down-sampled as appropriate to avoid bias. For details of other analyses, method references, and algorithm parameters and versions, please see the “Computational methods” section of the Supplemental Methods.

## Data access

All raw and processed sequencing data generated in this study have been submitted to the NCBI Gene Expression Omnibus (GEO; https://www.ncbi.nlm.nih.gov/geo/) under accession number GSE145931.

## Supplementary Material

Supplemental Material
