# Supplementary material for: RBFOX splicing factors contribute to a broad but selective recapitulation of peripheral tissue splicing patterns in the thymus
Source: Genome Res. 2021 Nov;31(11):2022–34. doi: 10.1101/gr.275245.121 (PMC8559713; doi:10.1101/gr.275245.121)
Supplement: Supplemental Material [file supp_31_11_2022__DC1.html]

RBFOX splicing factors contribute to a broad but selective recapitulation of peripheral tissue splicing patterns in the thymus — Supplemental Material 

# RBFOX splicing factors contribute to a broad but selective recapitulation of peripheral tissue splicing patterns in the thymus

## Supplemental Material

- Supplemental\_Material.pdf
- Supplemental\_Tables.xlsx
