# Supplementary material for: RBFOX splicing factors contribute to a broad but selective recapitulation of peripheral tissue splicing patterns in the thymus
Source: Genome Res. 2021 Nov;31(11):2022–34. doi: 10.1101/gr.275245.121 (PMC8559713; doi:10.1101/gr.275245.121)
Supplement: Supplemental Material [file supp_gr.275245.121_Supplemental_Material_.pdf]

Supplemental Material

A transcriptomic census reveals that Rbfox contributes to a broad but selective recapitulation of peripheral tissue splicing patterns in the thymus

Kathrin Jansen, Noriko Shikama-Dorn, Moustafa Attar, Stefano Maio, Maria Lopopolo, David Buck, Georg A. Holländer, Stephen N. Sansom

Contents

I. Supplemental Methods
II. Supplemental Figure Legends
III. Supplemental Tables
IV. Supplemental References

Supplemental Methods

Extraction of thymic epithelial cells and assessment by flow cytometry

Thymic epithelial cells were extracted as previously described (Dhalla et al. 2020). In short, thymic lobes were incubated with Liberase (Roche) and DNase (Roche) in PBS for 30 min at 37 °C. The cells were incubated with magnetic beads for 15 min at room temperature followed by enrichment of CD45-negative cells using the AutoMACS Pro Seperator (Miltenyl Biotech).
Enriched cells were stained with antibodies against CD45-AF700 (1:1000, 30F11; BioLegend), EpCAM-PerCPCy5.5 (1:1000, G8.8; BioLegend), Ly51-PE (1:200, 6C3; BioLegend), UEA-1-Cy5 (1:500, Vector Laboratories, in-house labelled), MHCII-BV421 (1:1000, M5/114.15.2; BioLegend), CD80-PE-Cy5 (1:1000, 16-10A1, Biolegend), CD86-PE-Cy7 (1:1000, GL-1, Biolegend). Staining was performed at 4ºC in the dark. DAPI or the LIVE/DEAD Fixable Aqua Dead Cell Stain Kit (Thermo Scientific) was used as a live/dead staining (Supplemental Fig. 21). Cells were sorted using FACSAria III (BD Bioscience) and data was analyzed using the FlowJo software (version 10.5.0).

Two independent experiments were performed using a total of six 4-6 weeks old mice for each experimental group. Three independent experiments were performed with the double knockout (Rbfox1$^{lox/lox}$: Rbfox2$^{lox/lox}$:cre+/-). For statistical analysis the resulting cell frequencies were combined and two-sided Welch Two Sample *t*-tests were performed between genotypes (Fig. 6B-C, Supplemental Figures 13-15).

Isolation of thymocytes and analysis by flow cytometry

Thymi and spleens were dissected from knockout and wildtype mice and isolated by gently dissociating the tissues between two frosted glass slides. Cells were filtered and resuspended in PBS containing 2% FCS (Merck) and stained with a combination of the markers given in Supplemental Table 12. The staining for surface markers was performed for 20 min at 4 ºC in the dark. The cell viability was assessed using the LIVE/DEAD Fixable Aqua Dead Cell Stain Kit (ThermoFisher Scientific) according to the manufacturer's instructions. Cells were acquired using FACSAria III (BD Bioscience) and data was analyzed using FlowJo software (version 10.5.0). As described above, n=2 independent experiments were performed and statistical analysis were performed on combined data (Supplemental Fig. 16).

Preparation and staining of samples for confocal microscopy

Freshly dissected thymus lobes were frozen in OCT (Tissue Trek) and 8 µm tissue sections were cut using a Cryostat (Thermo Scientific CryoStar NX70 with MB DynaSharp Microtome Blade). The tissue sections were fixed for 20 min in 1.4 % PFA (Sigma, in 1xPBS) and for 10 min in Methanol (VWR). Sections were permeabilized for 10 min with 0.3 % Triton-X (Sigma, in 1xPBS). These steps were followed by one 5 min washing step in 1xPBS, marking of the individual sections by a hydrophobic PAP pen (Sigma) and two further 5 min washing steps of each individual section. The primary antibodies were diluted in 1xPBS containing 10% goat serum, 0.3% Triton-X and incubated for 45 min at 37 °C. Primary antibodies were directed against AIRE (5H12, eBioscience, 1:500) and the RRM domain (1:500). In addition, UEA-1 was used for identifying mTEC (1:150, Vector Laboratories, in-house labelled). After three washing steps, the secondary antibody was added (diluted 1:500 in 1xPBS, goat α rabbit- AF488 (Invitrogen), goat α rat- AF555 (Invitrogen))

and incubated for 30 min at 37 °C. After three washing steps, sections were exposed for 90 min to the lectin UEA-1 at 37°C followed by two additional washing steps and finally DAPI staining of nuclei (10 min, 1:10,000 dilution in methanol). After a final washing step slides were mounted using ProLong Gold antifade reagent (Invitrogen).

Long-read RNA-sequencing

After RNA extraction, 8µl of RNA was prepared for sequencing using the cDNA-PCR Sequencing Kit (SQK-PCS108, ONT) following manufacturer's protocol (version: PCS_9035_v108_revF_26Jun2017; update: 31/05/2018). cDNA libraries were amplified for 18 cycles and normalized to 400 fmol before sequencing on MinION flow cells (FLO-MIN 106 R9.4) for 48 hrs.

Computational methods

Publicly-available RNA-sequencing datasets

RNA-sequencing data for the 21 peripheral mouse tissues were obtained from the ENCODE Project (GSE36025; keeping only the colon samples to represent the large intestine). RNA-sequencing from skin epithelial cells, mTEC and cTEC (GSE44945) (St-Pierre et al. 2013), cTEC (GSE53111) (Sansom et al. 2014), and single mature mTEC (GSE114713) (Handel et al. 2018) was obtained from the Gene Expression Omnibus (GEO). Independent RNA-sequencing datasets for peripheral mouse tissues (GSE41637, (Merkin et al. 2012)) as well as MHCII high and low adult mTEC (GSE68190, (Chuprin et al. 2015)) were downloaded from GEO.

Generation of the mT&T transcriptome assembly

Sequence reads were trimmed to 76 bp (fastx trimmer v0.0.14) and mapped with HISAT2 (Kim et al. 2019) (v2.1.0; Ensembl mm10-v91; settings:"–dta –score -min L,0.0,-0.2 –rna-strandness RF"). To generate a single high-depth sample for each tissue and TEC population replicate samples were combined and downsampled to 200M reads (samtools (Li et al. 2009) v1.3.1; Supplemental Table 1).

Separate reference-guided assemblies were constructed for each ENCODE tissue or TEC population using the high-depth samples (StringTie (Pertea et al. 2015) v1.3.3b; Ensembl mm10-v91). To identify transcripts that were reproducibly detected we first prepared biological replicate sample pools for each tissue and TEC population (n=2; 60M reads/samples, Supplemental Table 1). Expression of the transcripts present in each of the assemblies was then quantified in the relevant tissue or TEC population using the two 60M replicates (Salmon (Patro et al. 2017), v0.11.3, with parameters: "--incompatPrior=0 --validateMappings --rangeFactorizationBins=4 --seqBias --gcBias -x 0.66"). We then implemented and applied a robust procedure based on computation of the non-parametric Irreproducibility Discovery Rate (npIDR)(Dobin et al. 2013; Pervouchine et al. 2015) (details below and Supplemental Fig. 1). Transcripts from each tissue or TEC population that were reproducibly detected according to this procedure (those expressed above a level determined to correspond to npIDR ≤ 0.1) were merged into a single unified assembly (StringTie, Ensembl reference annotation guided merge). Transcripts contained in reference introns, possible polymerase run-on fragments, repeats, transcripts overlapping opposite-strand exons or introns and possible pre-mRNA fragments were removed (gffcompare v.0.10.6, class codes 'irpxse'). To be included in the final mT&T assembly, the merged transcript models were additionally required to be reproducibly detected (i.e. expressed above a level determined to correspond to npIDR ≤ 0.1; npIDR was determined as described above) in at least one tissue or TEC population.

The final mT&T assembly was used as the annotation for all subsequent steps if not otherwise indicated.

Quantification of gene and transcript expression levels

For comparisons of gene and transcript expression between the ENCODE tissues and the TEC populations samples (generated for this study) the trimmed 76bp sequences were deduplicated (Picard v2.10.9), filtered to exclude unmapped reads, and down-sampled to common read depths. For comparisons of gene and transcript expression between the Merkin et al. mouse tissues (Merkin et al. 2012) and the St-Pierre and Chuprin TEC samples (St-Pierre et al. 2013; Chuprin et al. 2015) sequence reads were trimmed to 50 bp, deduplicated, filtered to exclude unmapped

reads, and down-sampled to common read depths. Transcripts-per-million (TPM) values were obtained using Salmon with an index created from the new mT&T assembly (k=31 for index, settings: "ISR --gcBias") and upper-quartile normalized. Reads were counted by featureCounts (Liao et al. 2014) (v1.6.0).

RNA-sequencing reads from the *Rbfox1* and *Rbfox2* tKO mice were not trimmed but were otherwise mapped, deduplicated, filtered to remove unmapped reads, downsampled and quantitated as described above (retaining 14.5M and 10M paired-end reads/replicate for mature and immature mTEC, respectively).

Procedure for identification of reproducibly detected transcripts

To identify reproducibly detected transcripts, we implemented a robust procedure based on the npIDR metric (Dobin et al. 2013; Pervouchine et al. 2015) because we noted very lowly expressed transcripts to frequently pass a naïve npIDR filter (data not shown). TPM values were first log10 transformed (after addition of a small pseudocount = 0.001) and extreme values ($x < 0.5$ or $x > 0.95$ expression quantile) were removed to avoid issues arising from data sparsity. Data were then binned (n=50 bins) and npIDR values for each bin computed as previously described (Pervouchine et al. 2015). To estimate the expression level above which transcripts could be reliably detected we modelled the TPM vs npIDR relationship by LOESS regression. The fitted curve was used to estimate the TPM value that corresponded to npIDR ≤ 0.1. The analysis was performed separately for each TEC and peripheral tissue (with n = 2 biological replicate sample pools). Determination of the TPM threshold above which transcripts were reproducibly detected in the adrenal samples is shown in Supplemental Fig 1A-B. The TPM thresholds determined for each of the TEC and tissue samples are shown in Supplemental Fig 1C. This procedure was more consistent and conservative for our datasets than the use of per-transcript npIDR values (data not shown).

Identification of novel tissue-restricted transcripts

Novel transcripts were defined as those without a match in the Ensembl annotation (as assessed with gffcompare). Tissue-restricted novel transcripts were identified as those with *tau*>0.99

(Kryuchkova-Mostacci and Robinson-Rechavi 2017) in the representative tissues (Supplemental Fig. 3A) and wildtype mature mTEC. In addition, they were required to have an expression level that was >2 fold higher in a single representative tissue vs all of the other representative tissues (Fig. 3B and Supplemental Fig. 8). *Tau* values were computed using upper-quartile normalised, log2(n+1) transformed TPM values from the representative tissues. Novel transcripts were annotated using the *generateEvents* function in SUPPA (Alamancos et al. 2015) (v2.3) (Fig. 3C).

Identification of tissue-restricted antigen and *Aire*-regulated genes

Tissue-restricted antigen (TRA) genes were defined as the set of protein-coding genes that showed evidence of tissue restricted expression amongst the ENCODE tissues and wildtype mTEC samples. To avoid representation bias when computing expression specificity, we first identified groups of similar peripheral tissues by hierarchically clustering the tissues according to their transcript expression profiles. One tissue was then selected to represent each of the groups identified (Supplemental Fig. 3A). The set of TRA genes was defined as the set of genes with *tau* values > 0.7 in the representative tissues and wildtype immature and mature mTEC samples (Supplemental Fig. 3B) (Kryuchkova-Mostacci and Robinson-Rechavi 2017). To associate TRA genes with individual tissues we constructed a family (*F*) of non-overlapping TRA gene subsets which we termed “iTRA” by assigning TRA genes to the tissue in which they were most highly expressed. In summary, $F_{\mathrm{iTRA}} = \{\mathrm{iTRA}_{\mathrm{tissue}\text{-}i}, \dots, \mathrm{iTRA}_{\mathrm{tissue}\text{-}n}\}$ where $\mathrm{iTRA}_{\mathrm{tissue}\text{-}i}$ comprises the subset of TRA genes with highest expression in tissue *i*. As a concrete example, the adrenal iTRA subset (denoted $\mathrm{iTRA}_{\mathrm{adrenal}}$ or simply “adrenal iTRA”) contains the subset of TRA genes that were more highly expressed in the adrenal tissue than in any other peripheral tissue. *Aire*-regulated genes were defined as those significantly downregulated more than 2-fold in the homozygous *Aire*-knockout mTEC relative to heterozygous *Aire*-knockout mTEC (BH adjusted $p < 0.05$, DESeq2 (Love et al. 2014) analysis, n=2 biological replicates, Supplemental Fig. 3C). For the identification of *Aire*-regulated genes in mTEC untrimmed, full-depth sequence data was mapped (as above) and quantified using featureCounts (Ensembl v91). In total, we identified n=3,889 *Aire*-regulated tissue-restricted antigen genes (*Aire*-TRA), n=5,266 other tissue-restricted antigen genes (non-*Aire* TRA) and n=12,885 non-TRA genes (Supplemental Fig. 3D and Supplemental Table 2).

For comparisons of gene and transcript expression between the Merkin et al. data of mouse peripheral tissues (Merkin et al. 2012) and the corresponding data from St-Pierre et al. and Chuprin et al. TEC samples (St-Pierre et al. 2013; Chuprin et al. 2015) TRAs were identified as genes with a *tau* value > 0.7 in the Merkin et al. peripheral tissues (as applied to the upper-quartile normalised, log2(n+1) transformed TPM values). Subsets of iTRAs were then identified for these tissues following the approach described above for the ENCODE tissues.

Assessment of differential splicing and splice junctions

Differential splicing events were identified using rMATS (Shen et al. 2014) (v3.2.5; default settings, filtered for FDR<0.05; mapping with mT&T assembly as annotation). Exon percentage spliced-in (PSI) values were computed using SUPPA (3' and 5' UTR regions excluded; event-centric mode). For Fig. 4A, two biological replicate TEC samples (each from multiple thymi) with 130 Mio reads each were used. For Supplemental Fig. 9A, rMATS was run using two biological replicate samples (each from individual animals) with 19.5 Mio reads each for the peripheral tissue or TEC. Details of the samples and RNA-sequencing strategy for the rMATS alternative splicing analyses are summarised in Supplemental Table 14.

Splice junctions (SJs) were counted using SJcounts (v3.1; settings: "-maxnh 1 -read1 0 -read2 1") (Pervouchine et al. 2013) using 50M trimmed (76bp), deduplicated, mapped reads/sample. The results were post-processed to split the multi-junction counts into individual junction counts for final quantitation. SJs were assigned to genes by separately intersecting their start coordinates (±3bp) and end coordinates (±3bp) with exon coordinates extracted from protein-coding transcripts (Ensembl v91, bedtools window (v2.25.0)). Only SJs for which both the start and end coordinates intersected with exons from protein-coding transcripts from the same protein-coding gene were included in downstream analyses. For Fig. 2, and Supplemental Fig. 5 the numbers of unique SJs per gene were summarised. Significant differences in the number of unique junction counts were identified using edgeR (v3.32.0, with housekeeping genes used to estimate dispersion from the single replicates). For this analysis the set of mouse housekeeping genes was defined as n=473

genes which were 1:1 orthologs of a published set of human housekeeping genes (de Jonge et al. 2007). Differences in junction number were considered significant if they showed a two-fold change in number and an FDR < 0.05.

Definition of *Aire*-regulated transcripts

Differential transcript expression between *Aire*-knockout and *Aire*-positive mTEC was assessed using kallisto (Bray et al. 2016) (v0.43.1, n=1,000 bootstraps, Ensembl v91) and the sleuth R package (Pimentel et al. 2017) (v0.29). A value of the sleuth 'b' parameter of log(1.77) was determined to correspond to a 2-fold change (by modelling of actual expression values from kallisto). Transcript lengths were obtained using the function *transcriptLengths* from the R package Genomic Features (v1.30.3).

Analysis of ONT data

Basecalling of ONT raw tracks was performed using Albacore (v2.1.10) and 'pass' reads (mean quality score of < 7) were trimmed using Porechop (v0.2.3). Merged reads were mapped with minimap2 (Li 2018) (v2.9-r720, settings: "-L -ax splice", mm10 genome). Alignments were filtered for mapping quality > 20. The number of splices per read and gap-compressed identity ('de' tag) were extracted from the resulting bam file. Gene expression was quantified using featureCounts (settings: "-s 0 -L –fracOverlap 0.8"). Validation of novel mT&T transcripts was performed after first excluding mT&T transcripts with retained introns (defined by SUPPA) and mT&T transcripts arising from genomic loci with overlapping gene models (on opposite strands).

Definition of tissue-restricted splicing-related factors

To assess the expression of splicing-related genes in ENCODE tissues and TEC populations, we compiled a list of known splicing related genes from (i) literature sources (Barbosa-Morais et al. 2006; Grosso et al. 2008; Chen and Manley 2009; Merkin et al. 2012; Han et al. 2013; Jangi and Sharp 2014; St-Pierre et al. 2015), (ii) the RNA-binding protein database (RBPDB) (Cook et al. 2011) and (iii) relevant Gene Ontology (GO) categories. From the RBPDB database (http://rbpdb.ccbr.utoronto.ca/, downloaded 1st June 2018) we included the mouse RNA-binding

protein genes. Genes were included from GO categories that matched the string ‘splic’ (but not ‘tRNA’ or ‘protein splicing’). GO data was retrieved from AmiGO or the GO Online SQL Environment (GOOSE) database (Carbon et al. 2009) (downloaded 29th May 2018). Tissue-restricted splicing-related genes were then identified as those with *tau* > 0.5 in the TEC and peripheral tissue samples.

Analysis of single-cell RNA-sequencing data

The data (GSE114713) was mapped (HISAT2, parameters: “--dta --score-min L,0.0,-0.2”), quality controlled and quantitated (Cufflinks v2.21, featureCounts, Ensembl v91) using pipeline_scrnaseq.py (https://github.com/sansomlab/scseq). 201 cells with < 50% ERCC spike-in sequences, > 50,000 read pairs, > 2500 genes (Cufflinks), > 5% spliced reads, < 50% duplication rate, <1.3 fold 3’ bias and > 70% high-quality reads aligned were retained for further analysis. Fractions of single cells expressing genes were determined based on counts from featureCounts.

Geneset over-representation and motif enrichment analysis

Geneset over-representation analyses were performed using one-sided Fisher’s exact tests (FETs) (https://github.com/sansomlab/gsfisher). The tool MATT (Gohr and Irimia 2019) was used to determine motif enrichment in proximity to significant skipped exon events (*matt rna_maps*, v1.3.0).

For geneset over-representation analysis of genes harbouring novel transcripts in mature mTEC (Fig. 3D) genes with upper-quartile normalized, log2(n+1)-transformed TPMs > 0.1 were used as the background geneset. For Fig. 3D, we filtered genesets (GO-BP) for a minimum of four genes overlapping with the geneset of interest, for an odds ratio > 1.5 and p-adj < 0.05. From 70 genesets left after filtering, the most relevant, non-redundant (based on overlap of genes between the genesets) genesets were selected manually. All geneset are supplied in Supplemental Table 4. For the analysis of differentially spliced events in the *Rbfox2* tKO dataset (Fig. 7B), the foreground geneset was comprised of genes with differentially spliced events (|delta (d)PSI| > 0.2 and FDR < 0.05) and the background geneset comprised of the set of genes that were tested for differential

splicing events (rMATS analysis). The comparison of genes containing differential splicing events between *Rbfox1* and *Rbfox2* tKO was performed using a two-sided FET.
As input for the motif enrichment tool MATT, exons with enhanced inclusion or exclusion (|dPSI| > 0.2, FDR < 0.05) in the *Rbfox2* tKO samples relative to their Cre- littermate controls were used. Unregulated exons (|dPSI| < 0.05) were used to compute a control enrichment profile.

Downstream data analysis and data visualisation

Data analysis was performed in Python (jupyter notebooks; pandas v0.17.1) or R (RStudio; R v3.4). Heatmaps were leaf-optimised using the R cba library (v0.2-17). Genomic tracks were visualised using the R package Gviz (v1.22.3) or the *sashimi_plot* function from MISO (Katz et al. 2010) (v0.5.3).

Testing of linear model fits to different sample groups was performed by ANCOVA analysis (Fig. 1C, Supplemental Fig. 4). First, we tested for a difference in slope by testing for a significant interaction between the group and independent variables. If there was not a significant difference (p > 0.05), we concluded that there was no difference in slope and proceeded to test for a difference in intercept by fitting a second linear model without an interaction term. Model tests were performed using the R Anova function. Where a significant difference in intercept was observed (p <0.05) we used the adjusted mean values to summarise the locations of the groups.

Supplemental Figures

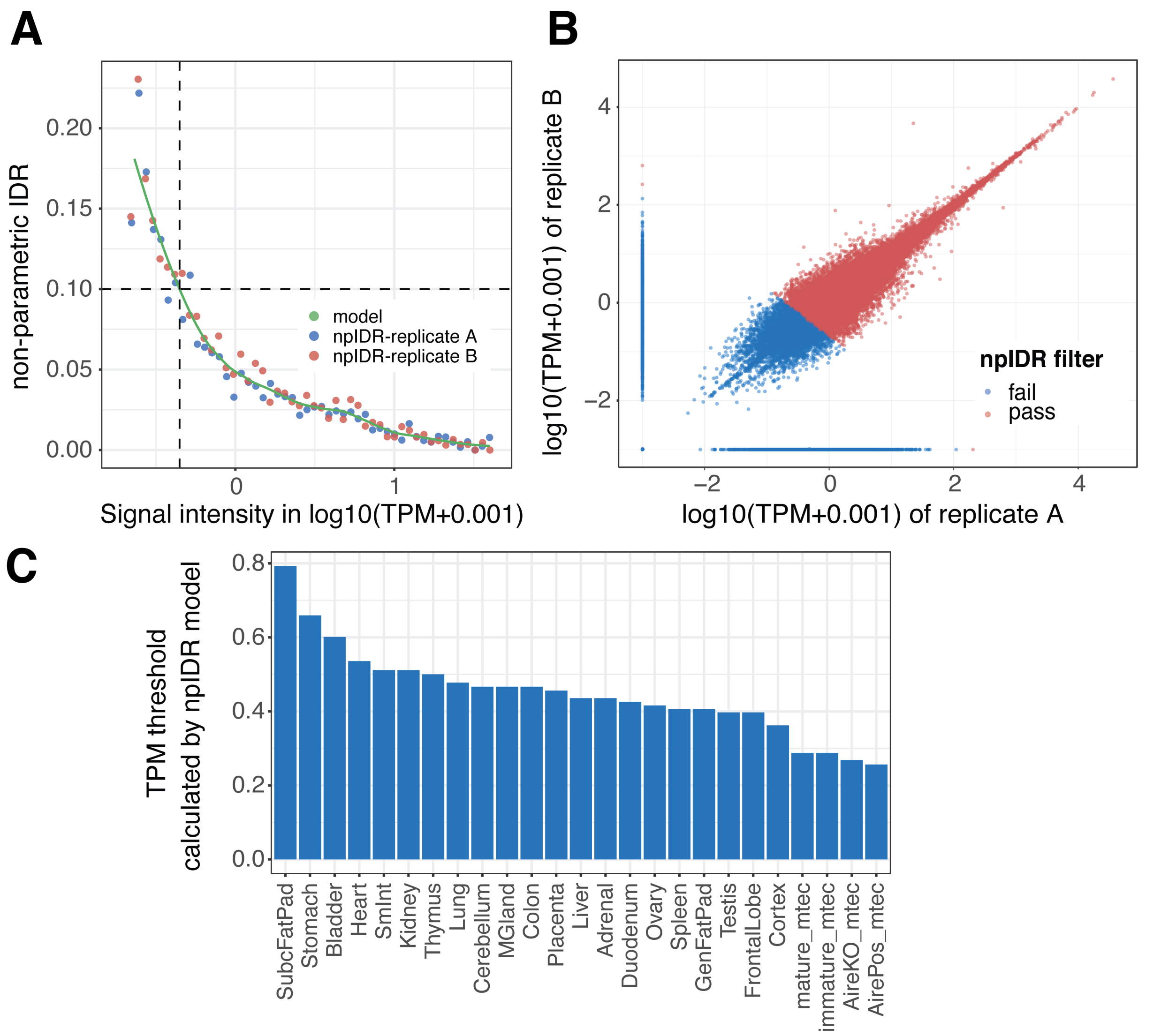

Supplemental Figure 1: Development of a robust procedure for identification of reproducibly detected transcripts. We noted that transcripts with very low expression values frequently passed a naïve npIDR filter. We therefore developed a more robust procedure in which the computed npIDR values are used to estimate a TPM threshold at which transcripts can be reliably detected (see Supplemental Methods). (A) Estimation of TPM thresholds for the selection of reproducibly detected transcripts. The scatter plot illustrates the relationship between the log10 expression level (TPM) and npIDR using the data from the adrenal samples. For each peripheral tissue and TEC subpopulation this relationship was modelled by LOESS regression (green curve) and the fitted curves used to estimate TPM thresholds that corresponding to npIDR values of 0.1

(dashed lines). (B) Identification of reproducibly detected transcripts based on the defined TPM threshold. The scatter plot shows transcript expression levels for the two adrenal samples. The color indicates whether a transcript is passes (red) or fails (blue) the npIDR filter as determined using the TPM threshold from panel B. (C) The TPM thresholds determined to correspond to npIDR ≤ 0.1 for each of the TEC and peripheral tissue samples.

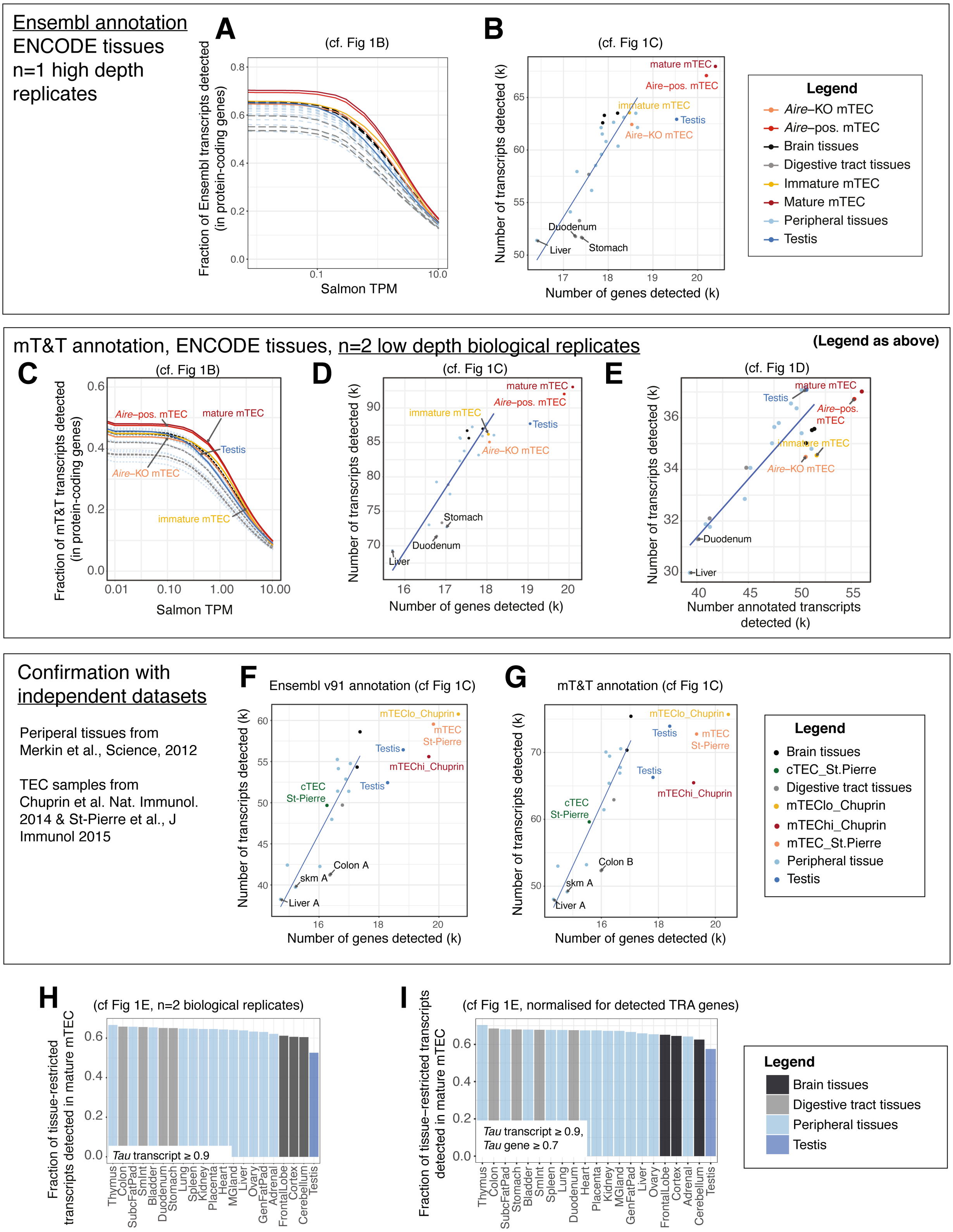

Supplemental Figure 2: Confirmation of differences between TEC and peripheral tissue transcriptomes. We confirmed the observed differences between TEC and peripheral transcriptomes (see Fig. 1) using (i) reference Ensembl (v91) annotations (A, B), (ii) mean statistics from lower depth biologically replicate samples pools (C-E) (n=2, 19.5M de-duplicated

mapped reads/sample, Supplemental Table 1) and (iii) with independent sets of peripheral tissue samples and TEC samples (F-G). For confirmation with independent datasets, we used mouse peripheral tissue RNA-seq data from Merkin et al. (Merkin et al. 2012) together with the mTEC population RNA-seq samples from Chuprin et. al. (Chuprin et al. 2015) and St. Pierre et al (7 day old, (St-Pierre et al. 2013)). Trend lines in B, D-G were fitted to all samples except TEC and testis. We also confirmed the patterns of tissue-restricted transcript (*tau* ≥ 0.9) representation in TEC (Fig. 1E) using (i) the lower depth biologically replicate sample pools (H) and (ii) after first normalising the fractions for the number of detected TRA genes (*tau gene* ≥ 0.7) (I).

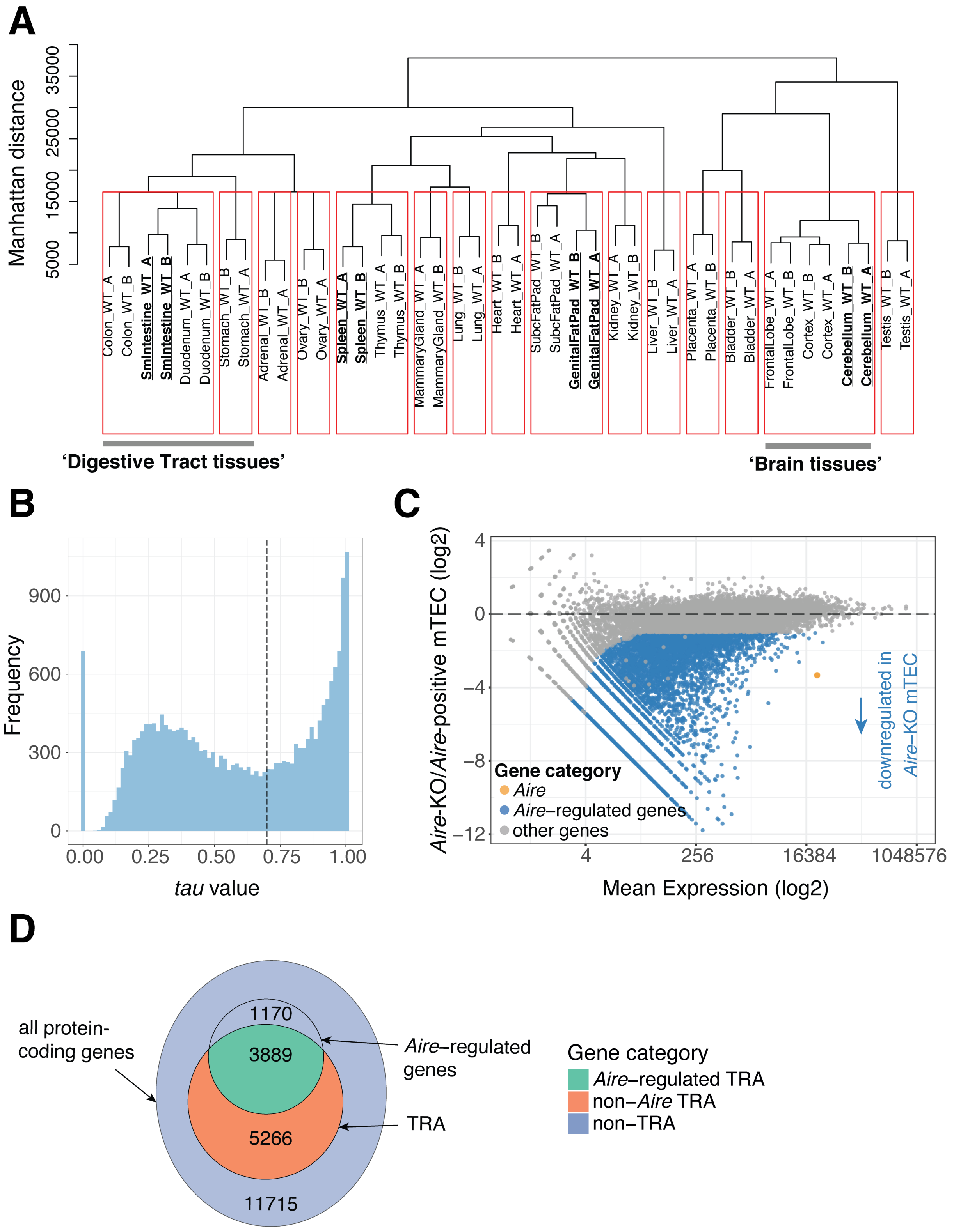

Supplemental Figure 3: Definition of tissue groups and identification of promiscuously expressed (tissue-restricted) genes. (A) Definition of groups of similar peripheral tissues. The mouse ENCODE tissue samples were hierarchically clustered by expression of known transcripts from protein-coding genes that showed variable expression (top n=24,664 most highly variable

known transcripts, Manhattan distance, complete linkage, n=2 replicate pools per tissue). Tissue groups (n=15; red boxes) were defined by cutting the dendrogram at a fixed height. The tissues taken as the representatives of the groups with multiple tissues are shown in bold font and underlined. (B) The histogram shows the distribution of the *tau* (Kryuchkova-Mostacci and Robinson-Rechavi 2017) values for all protein-coding genes. The *tau* values were computed using the selected tissues (A) and the wildtype immature and mature mTEC samples. Genes with a *tau* value of > 0.7 (as indicated by the dashed vertical line) were identified as tissue-restricted antigens (TRA). (C) Identification of *Aire*-regulated genes by differential expression analysis of protein-coding genes between *Aire*-knockout and *Aire*-positive mature mTEC (n=2 biological replicates). Genes with a significant, >2-fold downregulation in the *Aire*-knockout were defined as *Aire*-regulated genes (BH-adjusted p < 0.05). Reads were quantitated with featureCounts (Liao et al. 2014) (Ensembl v91 annotations) and differential expression analysis performed using DESeq2 (Love et al. 2014). (D) Comparison of tissue-restricted genes (*tau* > 0.7) and *Aire*-regulated genes. The diagram shows the three categories of *Aire*-regulated TRA (*Aire*-TRA), non-*Aire*-regulated TRA (non-*Aire* TRA) and non-TRA which are used throughout the manuscript.

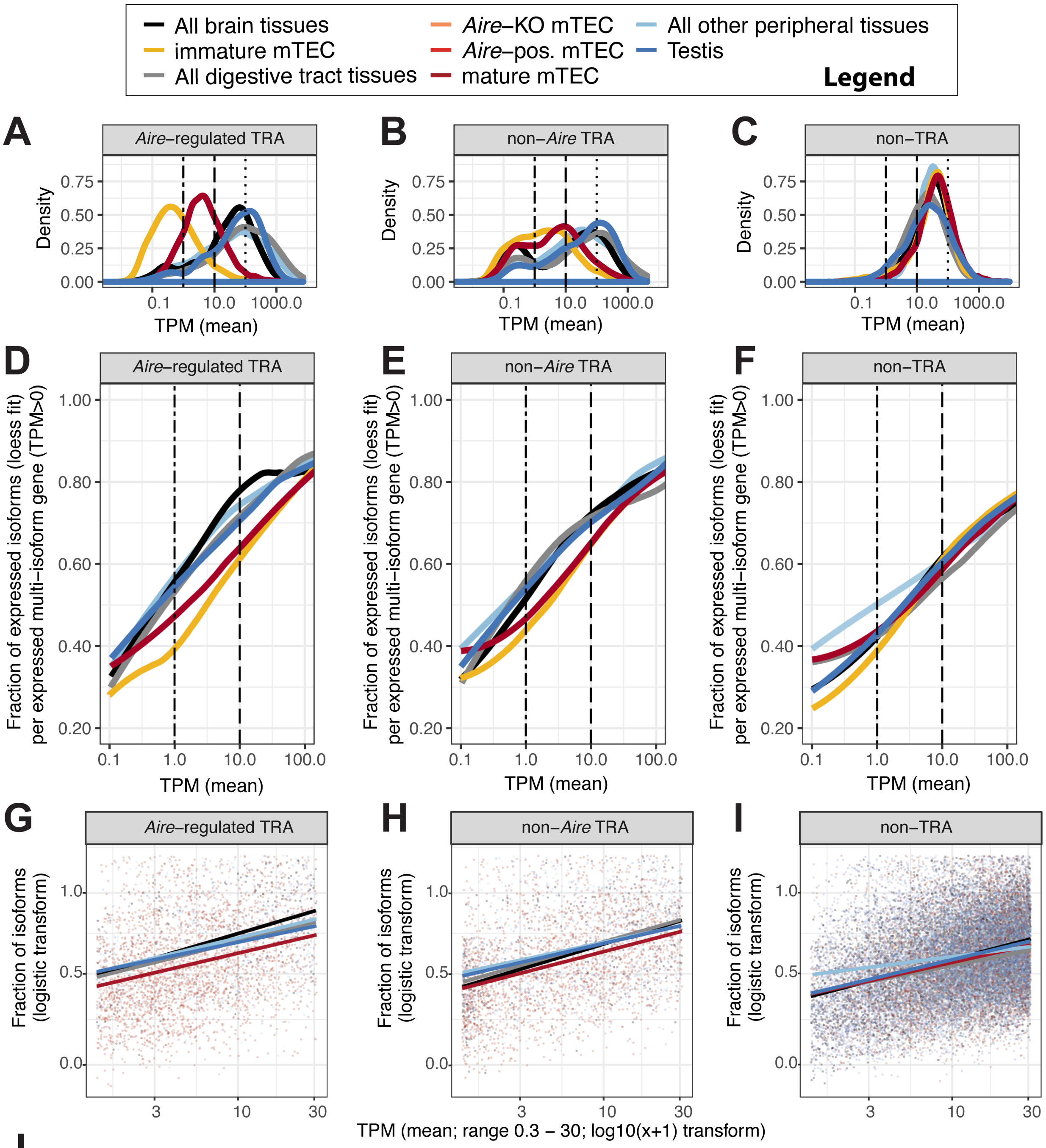

J

gene type	tissue A	tissue B	slope p value	intercept p value	fraction isoforms tissue A	fraction isoforms tissue B	difference in fractions	difference in fractions (%)
non-TRA	Brain regions	mature mTEC	0.00207 **	NA	NA	NA	NA	NA
non-TRA	Digestive tract tissues	mature mTEC	0.0128 *	NA	NA	NA	NA	NA
non-TRA	periphery	mature mTEC	2.44×10^{-12} ***	NA	NA	NA	NA	NA
non-TRA	Testis	mature mTEC	0.218	2.57×10^{-05} ***	0.61	0.592	0.018	2.95%
Aire-regulated TRA	Brain regions	mature mTEC	0.109	7.7×10^{-13} ***	0.7	0.591	0.109	15.6%
Aire-regulated TRA	Digestive tract tissues	mature mTEC	0.77	0.00334 **	0.654	0.588	0.0664	10.1%
Aire-regulated TRA	periphery	mature mTEC	0.812	2.7×10^{-16} ***	0.682	0.592	0.0903	13.2%
Aire-regulated TRA	Testis	mature mTEC	0.523	6.75×10^{-06} ***	0.658	0.59	0.068	10.3%
non-Aire TRA	Brain regions	mature mTEC	0.133	0.00479 **	0.65	0.609	0.0417	6.41%
non-Aire TRA	Digestive tract tissues	mature mTEC	0.632	0.0203 *	0.663	0.607	0.0562	8.48%
non-Aire TRA	periphery	mature mTEC	0.0602 .	7.46×10^{-11} ***	0.671	0.612	0.0584	8.71%
non-Aire TRA	Testis	mature mTEC	0.28	7.28×10^{-07} ***	0.66	0.609	0.0509	7.71%

Supplemental Figure 4: TEC express fewer isoforms of TRA genes. (A-C) TEC express *Aire*-regulated TRA and non-*Aire* TRA genes at lower levels than is found in periphery. The density plots show the TPM distributions of these genes for the testis, sets of peripheral tissues (for legibility mean TPM values are shown for all brain tissues, all digestive tract tissues and all other peripheral tissues) and the immature and mature mTEC cell populations. The three dashed vertical lines correspond to TPM values of 1, 10 and 100. (D-F) Detection of a lower-fraction of isoforms from (multi-isoform) *Aire*-regulated and non-*Aire* TRA genes in TEC is independent of gene expression level. The relationship between gene expression level (x axis) and isoform detection (y axis) for the testis, sets of peripheral tissues (sets of tissues defined as for A-C; mean isoform fractions) and immature and mature mTEC (LOESS regression curves). (G-J) To formally test for expression-level independent differences in the fraction of TRA isoforms expressed between mature TEC and the peripheral tissue groups we fitted linear models to the commonly linear portions of the relationships (0.3 < TPM < 30) after first performing a logistic transform of the fractions and a log10(x+1) transform of the expression levels (G-I). We tested for difference in the slope and intercept using an ANCOVA-based approach (Supplemental Methods). If a significant difference in slope (i.e. a significant interaction) was found, a difference in intercept was not tested for. The results are summarized in (J) with adjusted mean fractions and adjusted mean fraction differences shown where significant differences in the intercepts were observed. Analyses for all panels were performed using the high-depth samples (n=1). For the analyses of TRA in peripheral tissues gene statistics were only counted for the relevant iTRA subsets while the full set of TRA genes was quantitated in each of the TEC populations.

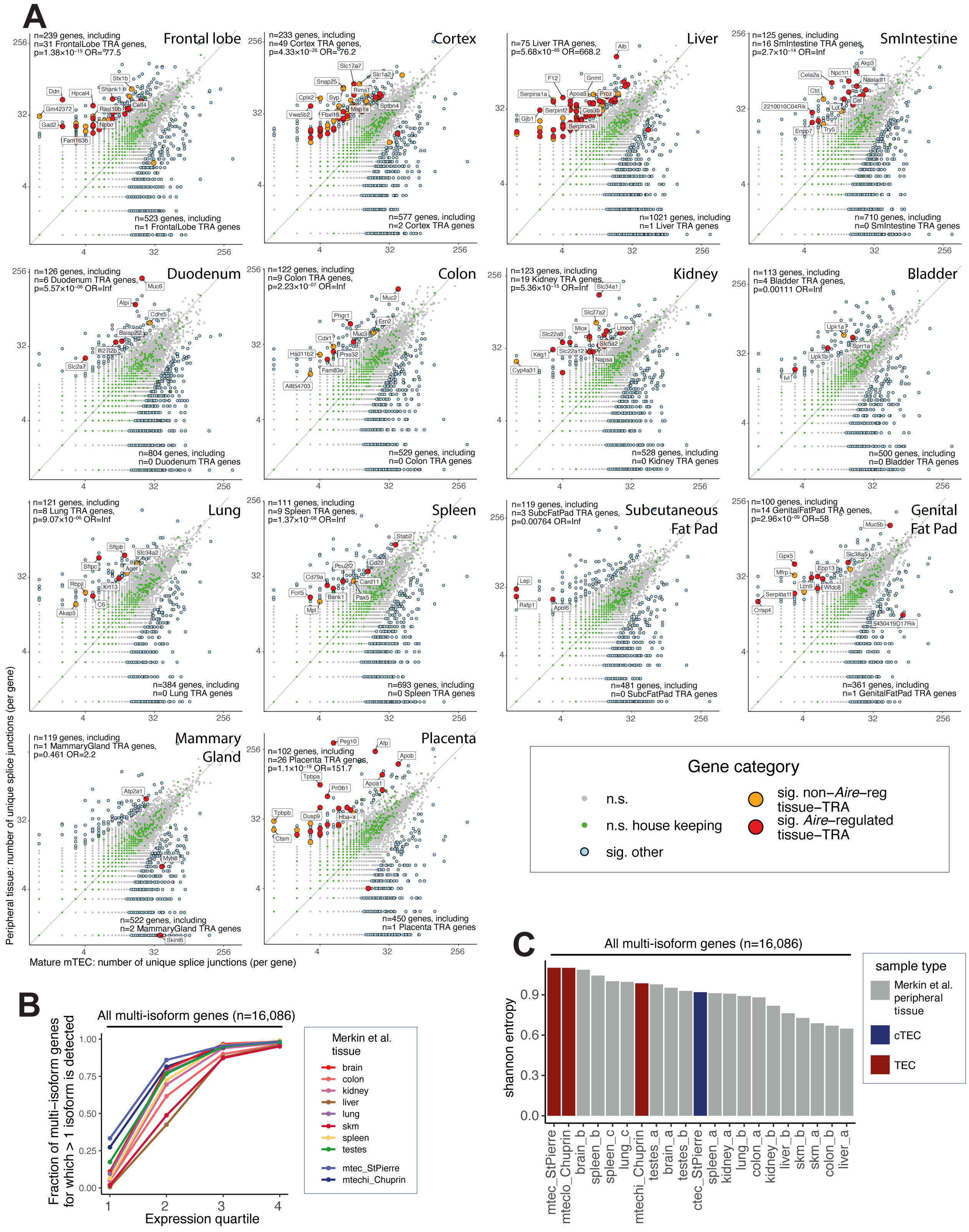

Supplemental Figure 5: Analysis of TRA splicing in individual tissues. (A) *Continued from Fig. 2B: the remaining n=14 ENCODE tissues not shown in the main figure are shown here*. The numbers of splice junctions found in protein-coding genes (points) in mature mTEC (this study; x axes) vs peripheral tissues (ENCODE; y axes). Significant (sig.) differences in junction number

were identified using edgeR (BH adjusted $p < 0.05$, $|fc| > 2$). P-values and odds ratios (OR) from Fisher's exact tests for enrichment of per-tissue iTRA gene subsets among the genes with significantly higher junction counts in the peripheral tissues are reported (at top left). The top 10 tissue iTRA genes with significant differences in junction counts are labelled (as ranked by edgeR p-value). (B, C) Comparison of splicing complexity in TEC and peripheral tissues using data from Merkin et al. (Merkin et al. 2012) and TEC data from St-Pierre et al. (St-Pierre et al. 2013) and Chuprin et al. (Chuprin et al. 2015) *These panels correspond to those shown for sets of per-tissue iTRA genes in Fig. 2C and D.* Here, as was reported previously (Keane et al. 2015; Danan-Gotthold et al. 2016), we confirm that when all multi-isoform genes are considered without regard for tissue specificity, (B) more alternatively spliced genes can be found in mTEC than are present in peripheral tissues (Danan-Gotthold et al. 2016), and (C) a higher mean Shannon entropy of per-gene isoform expression is observed in mTEC than in peripheral samples (Keane et al. 2015).

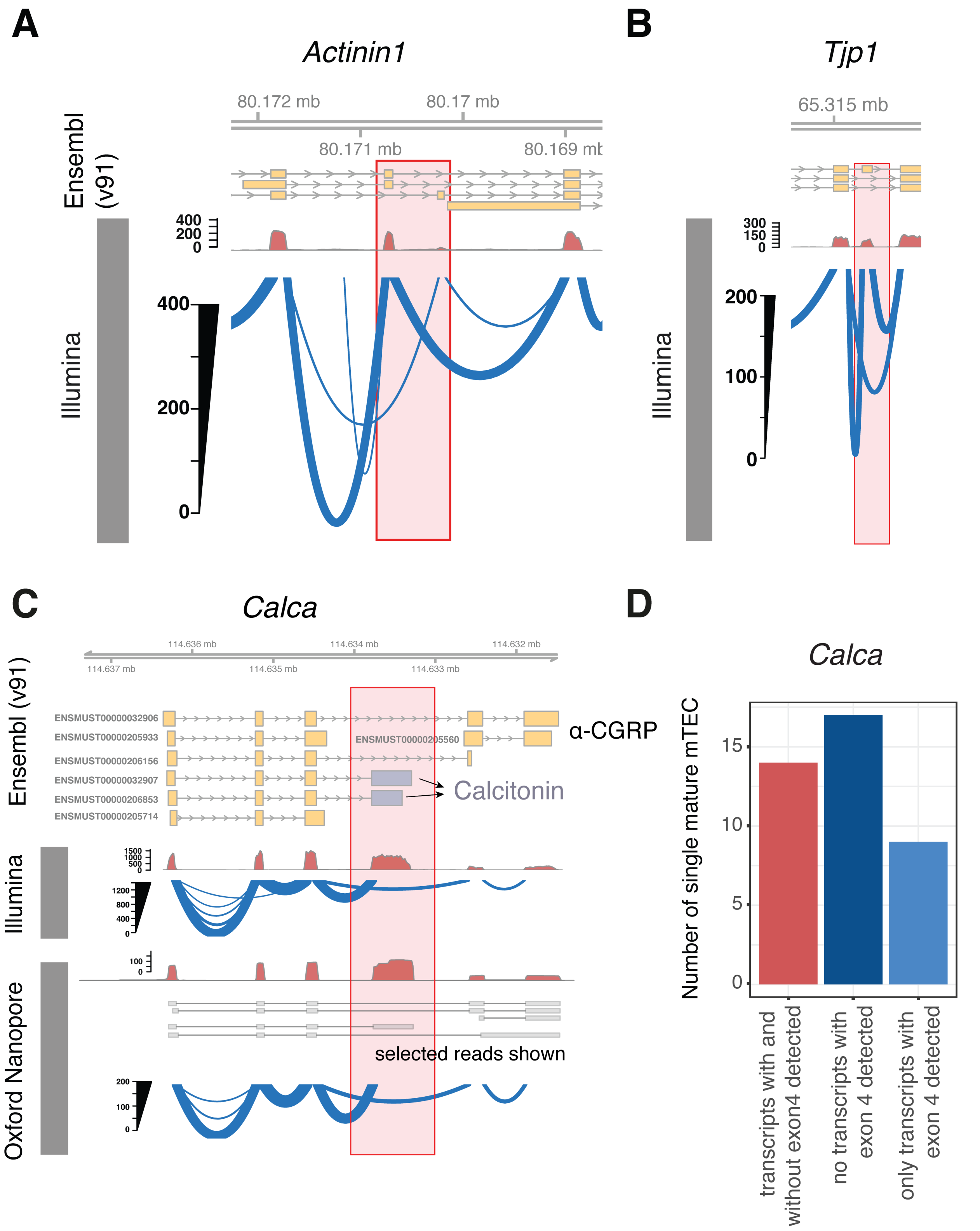

Supplemental Figure 6: Examples of known tissue-specific alternative splicing events recapitulated in TEC. (A) Transcript models, read coverage and sashimi plots for the gene *Actinin1*. Mature mTEC express both the muscular and non-muscular isoforms of *Actinin1* which are differentiated by expression of the indicated mutually exclusive exon pair (red box) (Gromak et al. 2003). (B) Transcript models, read coverage and sashimi plots for the gene *Tjp1*. Mature mTEC

express isoforms with and without exon 20 (red box). In the periphery inclusion of exon 20 is known to be highly tissue specific (Merkin et al. 2012). (C) Transcript models, read coverage and sashimi plots for the gene *Calca*. The shorter isoform, in which inclusion of exon 4 (red box) introduces a premature stop codon, is specific to the thyroid and produces the Calcitonin (CT) peptide (Chew 1997; Chen and Manley 2009). In the nervous system, exon 4 (red box) is skipped to form the longer α-CGRP isoform. Mature mTEC express both of these isoforms. For the Illumina sequencing, the sashimi plots in A-C were generated from the single high-depth sample of mature mTEC. Only splice junctions with coverage of ≥ 5 reads are shown. (D) Detection of exon 4 inclusion and exclusion in *Calca* transcripts in single mature mTEC. 35 % of the cells where *Calca* is detected produced *Calca* transcripts both with and without this exon.

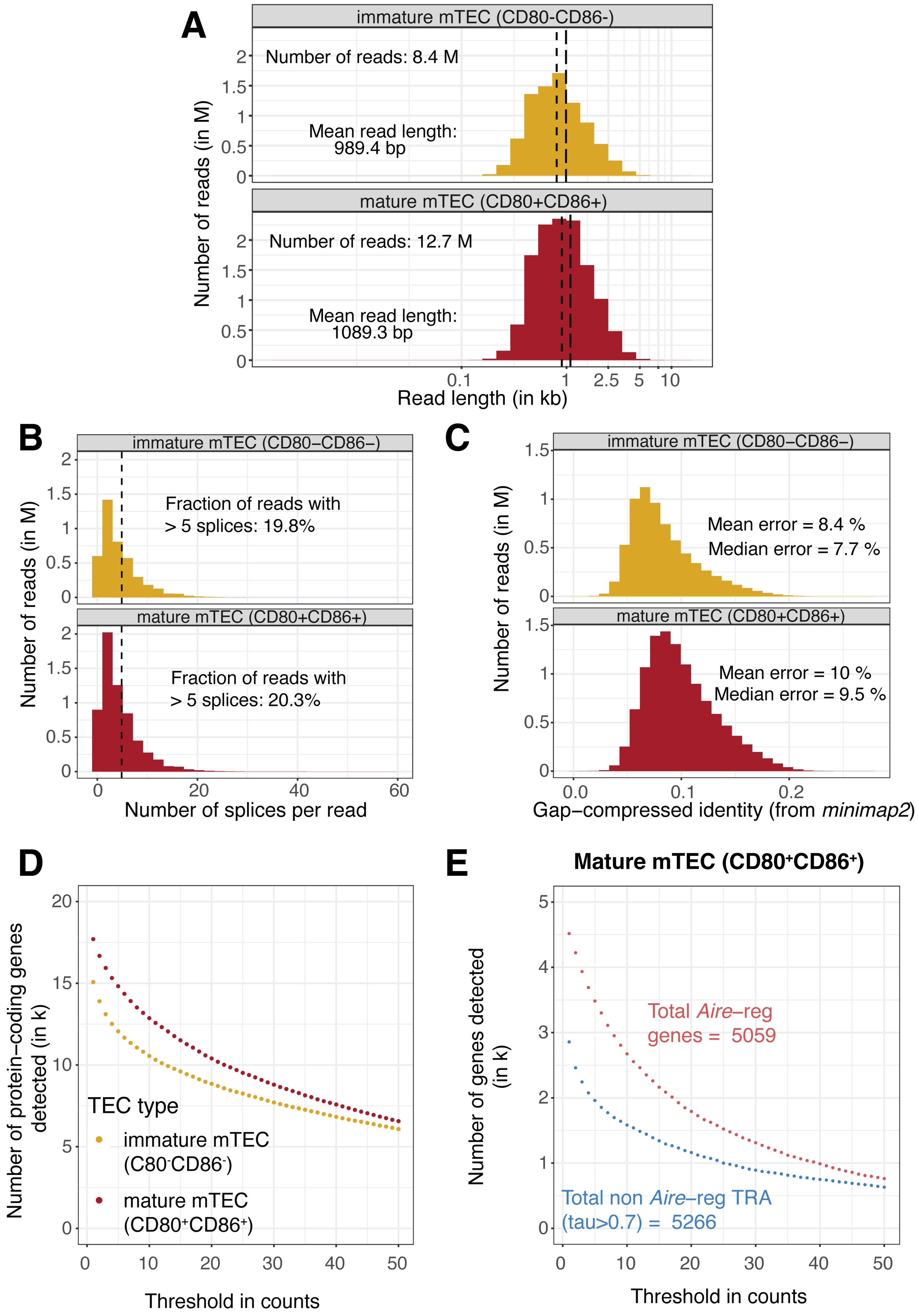

A
immature mTEC (CD80-CD86-)
Number of reads: 8.4 M
Mean read length: 989.4 bp
mature mTEC (CD80+CD86+)
Number of reads: 12.7 M
Mean read length: 1089.3 bp
Number of reads (in M)
Read length (in kb)
B
immature mTEC (CD80−CD86−)
Fraction of reads with > 5 splices: 19.8%
mature mTEC (CD80+CD86+)
Fraction of reads with > 5 splices: 20.3%
Number of reads (in M)
Number of splices per read
C
immature mTEC (CD80−CD86−)
Mean error = 8.4 %
Median error = 7.7 %
mature mTEC (CD80+CD86+)
Mean error = 10 %
Median error = 9.5 %
Number of reads (in M)
Gap−compressed identity (from minimap2)
D
Number of protein−coding genes detected (in k)
TEC type
immature mTEC (C80-CD86-)
mature mTEC (CD80+CD86+)
Threshold in counts
E
Mature mTEC (CD80+CD86+)
Number of genes detected (in k)
Total Aire−reg genes = 5059
Total non Aire−reg TRA (tau>0.7) = 5266
Threshold in counts

Supplemental Figure 7: Long-read sequencing of mTEC using Oxford Nanopore Technology. (A) Histograms of read length for Oxford Nanopore Technology (ONT) experiments. Immature and mature mTEC samples shown represent merged reads from three biological replicates. (B) Histograms of number of splices detected per read for the samples of merged ONT reads from immature and mature mTEC. (C) The mapping error for the merged ONT reads is estimated as 10 % or less. The Gap-compressed divergence is used as a measure of error (0 representing no errors in alignment, calculated using Minimap2). (D) The numbers of protein-coding genes detected in merged ONT read samples from mature and immature mTEC. Read were down-sampled to a common number for the two mTEC populations. (E) A large number of *Aire*-regulated genes was detected in the ONT data from mature mTEC. The number of detected *Aire*-regulated and tissue-specific genes (*tau* > 0.7) is assessed across a range of count thresholds using the merged ONT reads from mature mTEC.

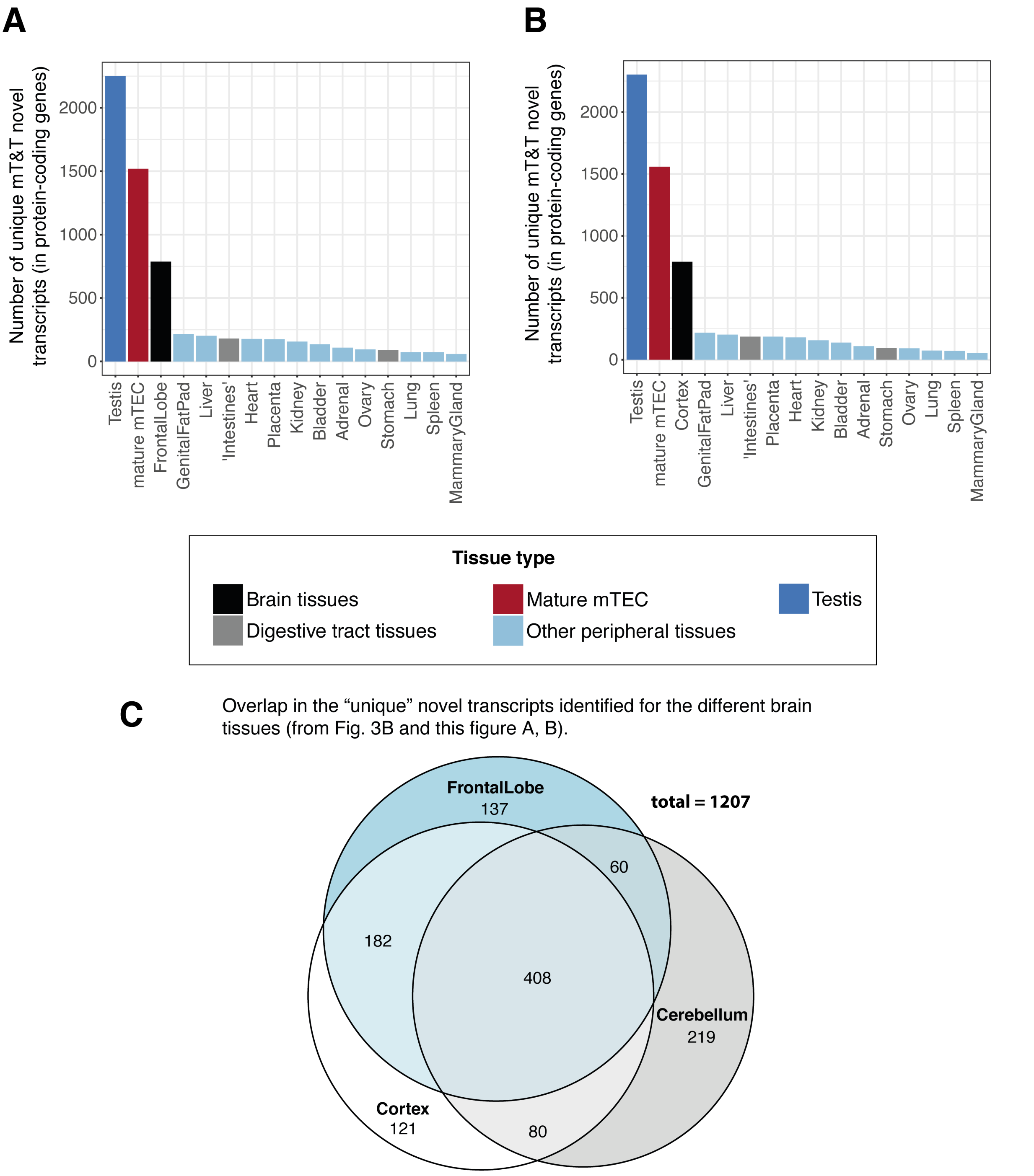

Supplemental Figure 8: Confirmation that similar numbers of unique novel transcripts are found in the different brain tissues. Analysis for Fig. 3B repeated with (A) the Frontal Lobe and (B) the Cortex sample used as the representative tissue for the group of brain tissues (see Supplemental Fig. 3A). (C) Venn diagram showing the overlap between the unique novel transcripts identified by using either Frontal Lobe, Cortex or Cerebellum as the representative tissue for the group of brain tissues.

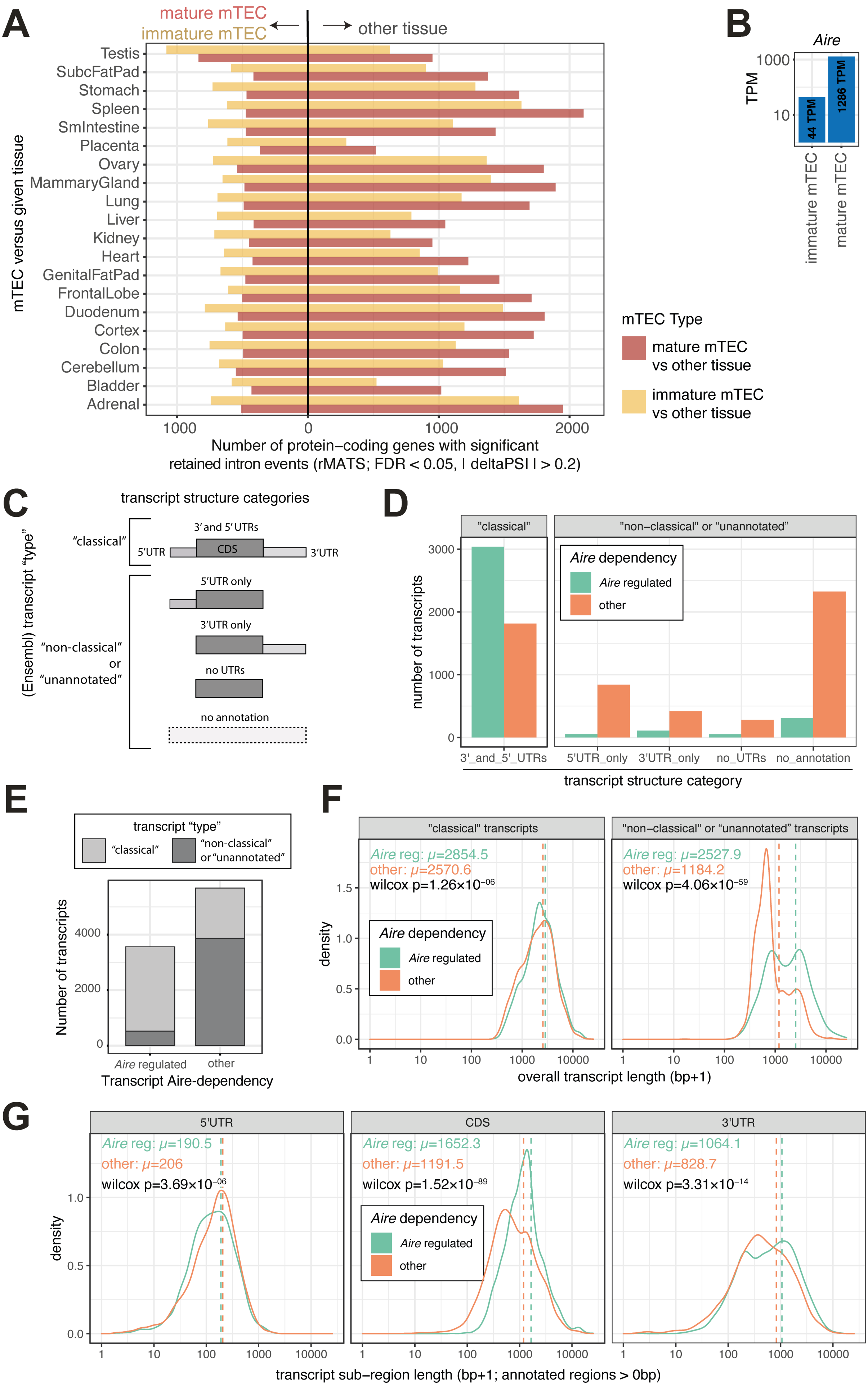
A
mature mTEC
immature mTEC
other tissue
mTEC versus given tissue
Testis
SubcFatPad
Stomach
Spleen
SmIntestine
Placenta
Ovary
MammaryGland
Lung
Liver
Kidney
Heart
GenitalFatPad
FrontalLobe
Duodenum
Cortex
Colon
Cerebellum
Bladder
Adrenal
1000
0
1000
2000
Number of protein−coding genes with significant
retained intron events (rMATS; FDR < 0.05, | deltaPSI | > 0.2)
mTEC Type
mature mTEC vs other tissue
immature mTEC vs other tissue
B
Aire
TPM
1000
10
44 TPM
1286 TPM
immature mTEC
mature mTEC
C
transcript structure categories
(Ensembl) transcript "type"
"classical"
3' and 5' UTRs
5'UTR
CDS
3'UTR
5'UTR only
3'UTR only
no UTRs
no annotation
"non-classical"
or
"unannotated"
D
"classical"
"non−classical" or "unannotated"
number of transcripts
3000
2000
1000
0
Aire dependency
Aire regulated
other
3'_and_5'_UTRs
5'UTR_only
3'UTR_only
no_UTRs
no_annotation
transcript structure category
E
transcript "type"
"classical"
"non-classical" or "unannotated"
Number of transcripts
4000
2000
0
Aire regulated
other
Transcript Aire-dependency
F
"classical" transcripts
"non−classical" or "unannotated" transcripts
Aire reg: μ=2854.5
other: μ=2570.6
wilcox p=1.26×10−06
Aire reg: μ=2527.9
other: μ=1184.2
wilcox p=4.06×10−59
density
1.5
1.0
0.5
0.0
Aire dependency
Aire regulated
other
1
10
100
1000
10000
overall transcript length (bp+1)
G
5'UTR
CDS
3'UTR
Aire reg: μ=190.5
other: μ=206
wilcox p=3.69×10−06
Aire reg: μ=1652.3
other: μ=1191.5
wilcox p=1.52×10−89
Aire reg: μ=1064.1
other: μ=828.7
wilcox p=3.31×10−14
density
1.0
0.5
0.0
Aire dependency
Aire regulated
other
1
10
100
1000
10000
transcript sub−region length (bp+1; annotated regions > 0bp)

Supplemental Figure 9: Additional retained intron and *Aire*-regulated transcript structure analyses (A) The numbers of protein-coding genes (x axis) in which significant differential intron retention events were detected. The numbers are given for the comparisons of immature or mature mTEC versus each peripheral tissue (n=2 replicates per sample, subsampled to same depth). (B) The expression level of *Aire* in immature and mature mTEC. (C-G) Analysis of the transcript structures produced by *Aire*-regulated genes in mature mTEC. The analysis was performed using Ensembl v91 annotations. *Aire*-regulated transcripts were identified as shown in Fig 4C. (C) The cartoon shows the structures of the different types of transcripts that were detected. These were broadly divided into (i) "classical" transcripts for which all of the 5' UTR, CDS and 3'UTR sub-regions had been annotated and (ii) "non-classical" or "unannotated" transcripts for which not all sub-regions were not present or had not been annotated. (D) The barplots show the numbers of the different types of transcript structures detected for the *Aire*-regulated and other transcripts. (E) The barplot shows that *Aire*-regulated transcripts were largely comprised of "classical" transcripts while the "other" (i.e. non-*Aire*-regulated) transcripts produced by *Aire*-regulated genes had "non-classical" or "unannotated" structures. (F) The density plots show the length distributions of "classical" vs "non-classical" or "unannotated" transcripts. Relative to their non-*Aire*-regulated counterparts the mean length of *Aire*-regulated transcripts was slightly increased for the "classical" transcripts (283.9bp; 11%) and greatly increased for the "non-classical" or "annotated" transcripts (1343.7bp; 213%). (G) Comparison of the lengths of annotated 5'UTR, CDS and 3'UTR transcript regions from the *Aire*-regulated and non-*Aire*-regulated transcripts produced by *Aire*-regulated genes. Relative to their non-*Aire*-regulated counterparts the mean length of the 5'UTRs from *Aire*-regulated transcripts was decreased by 15.5bp (7.6%). Relative to their non-*Aire*-regulated counterparts the mean length of the CDSs from *Aire*-regulated transcripts was increased by 460.8bp (38.6%). Relative to their non-*Aire*-regulated counterparts the mean length of the 3'UTRs from *Aire*-regulated transcripts was increased by 235.4bp (28.4%).

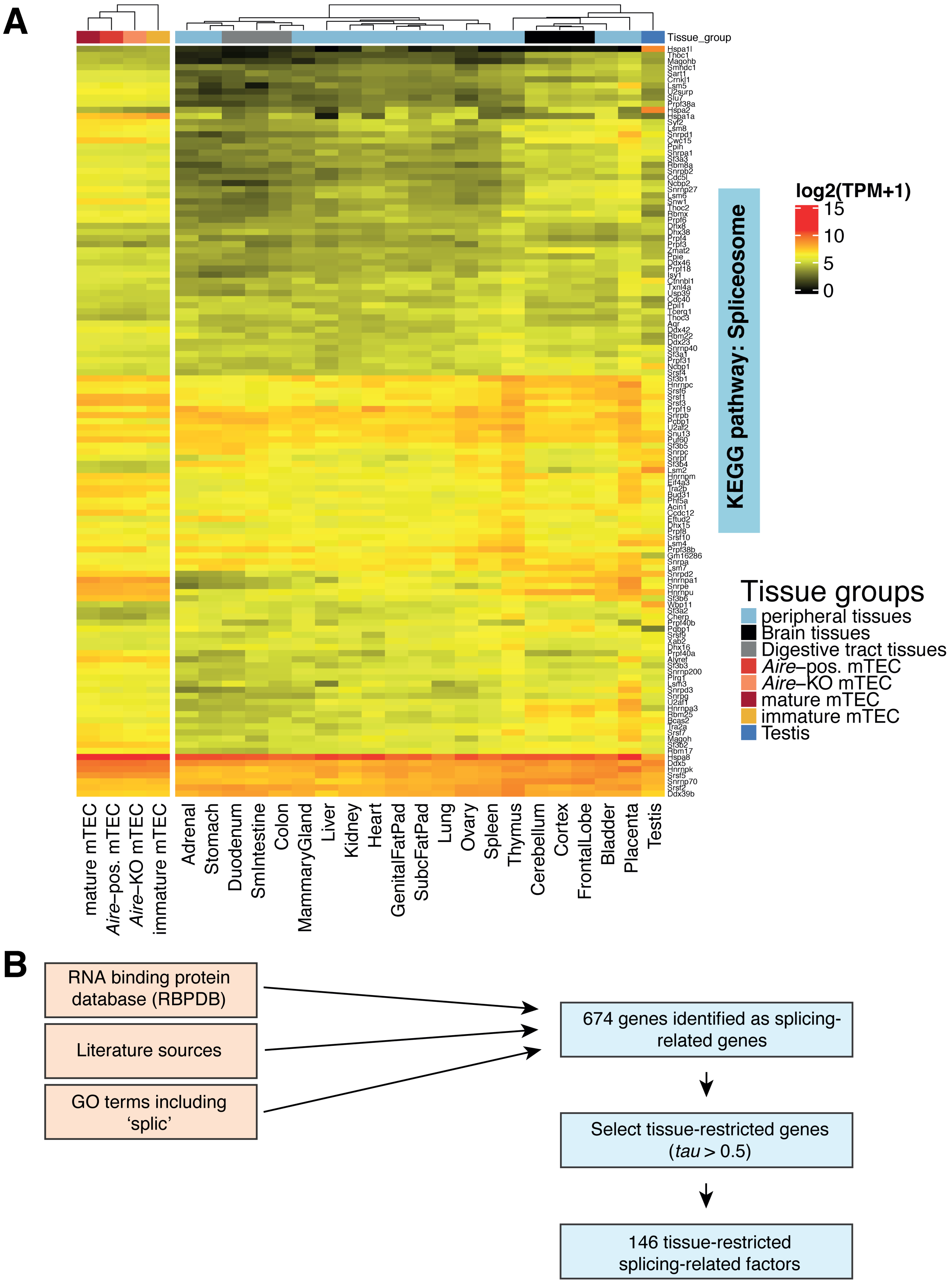

Supplemental Figure 10: Expression of spliceosome genes and identification of a set of tissue-restricted splicing factors. (A) Expression of all genes in the KEGG spliceosome pathway (ID: 03040) (taken to represent constitutive splicing factors). TEC populations did not show a

reduced expression of these genes compared to the peripheral tissues (mouse ENCODE Project). (B) Compilation of a list of tissue-restricted splicing-related factor genes (see Supplemental Methods).

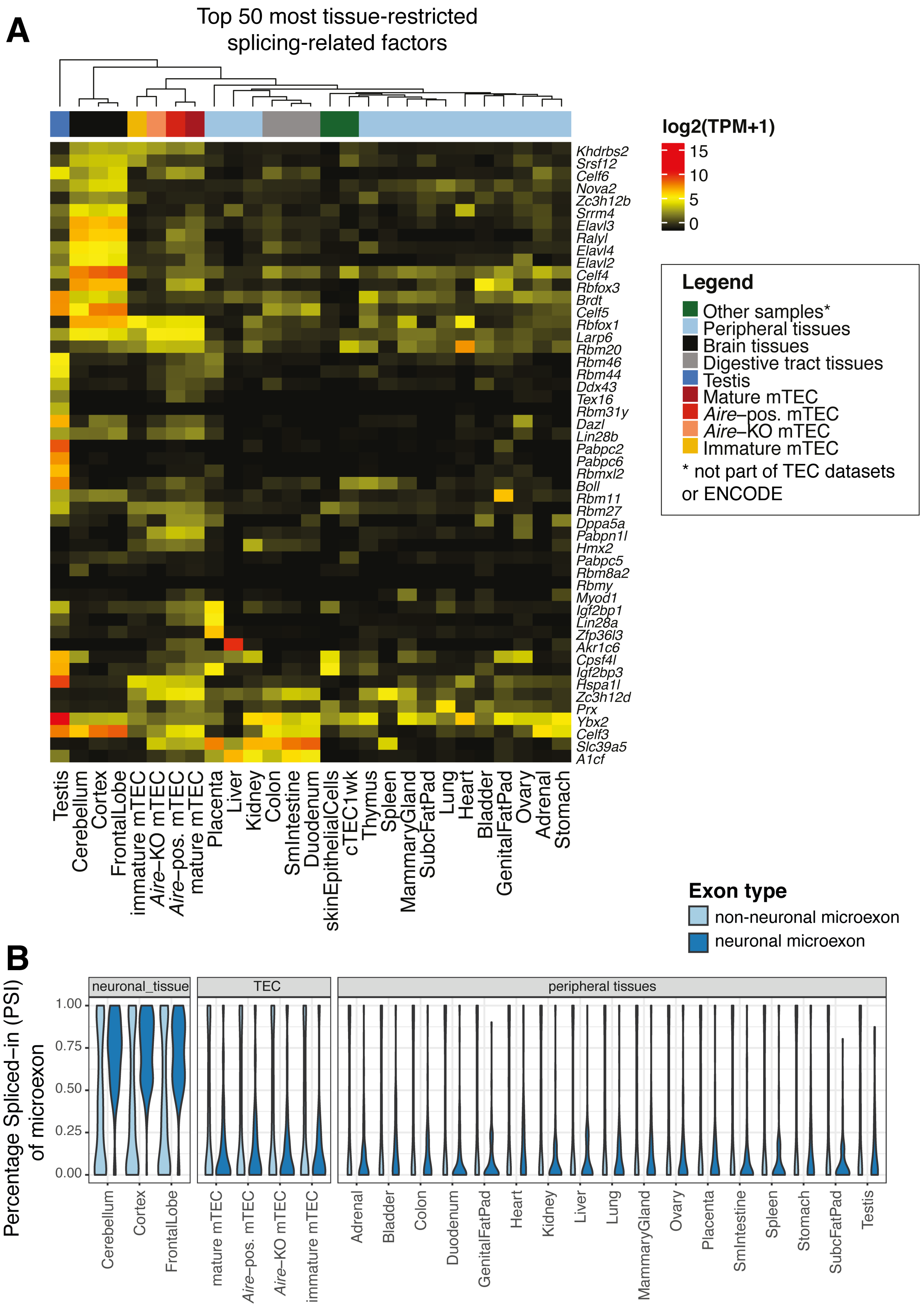

Supplemental Figure 11: Tissue-restricted splicing factor expression and micro-exon inclusion rates in TEC and peripheral tissues (A) Heatmap of expression level of the 50 tissue-

restricted splicing-related factors with the highest *tau* values across the included peripheral tissues and TEC subpopulations. Predicted genes (those beginning with ‘Gm’ and RIKEN clones) were excluded. (B) Violin plots showing the distribution of Percentage spliced-in (PSI) values of sets of neuronal (dark blue) and non-neuronal (light blue) microexons in neuronal tissues, TEC and other peripheral tissues. Neuronal microexons (dark blue) were defined as those having a mean PSI in neuronal tissues > 0.5 and a mean PSI in other tissues < 0.3.

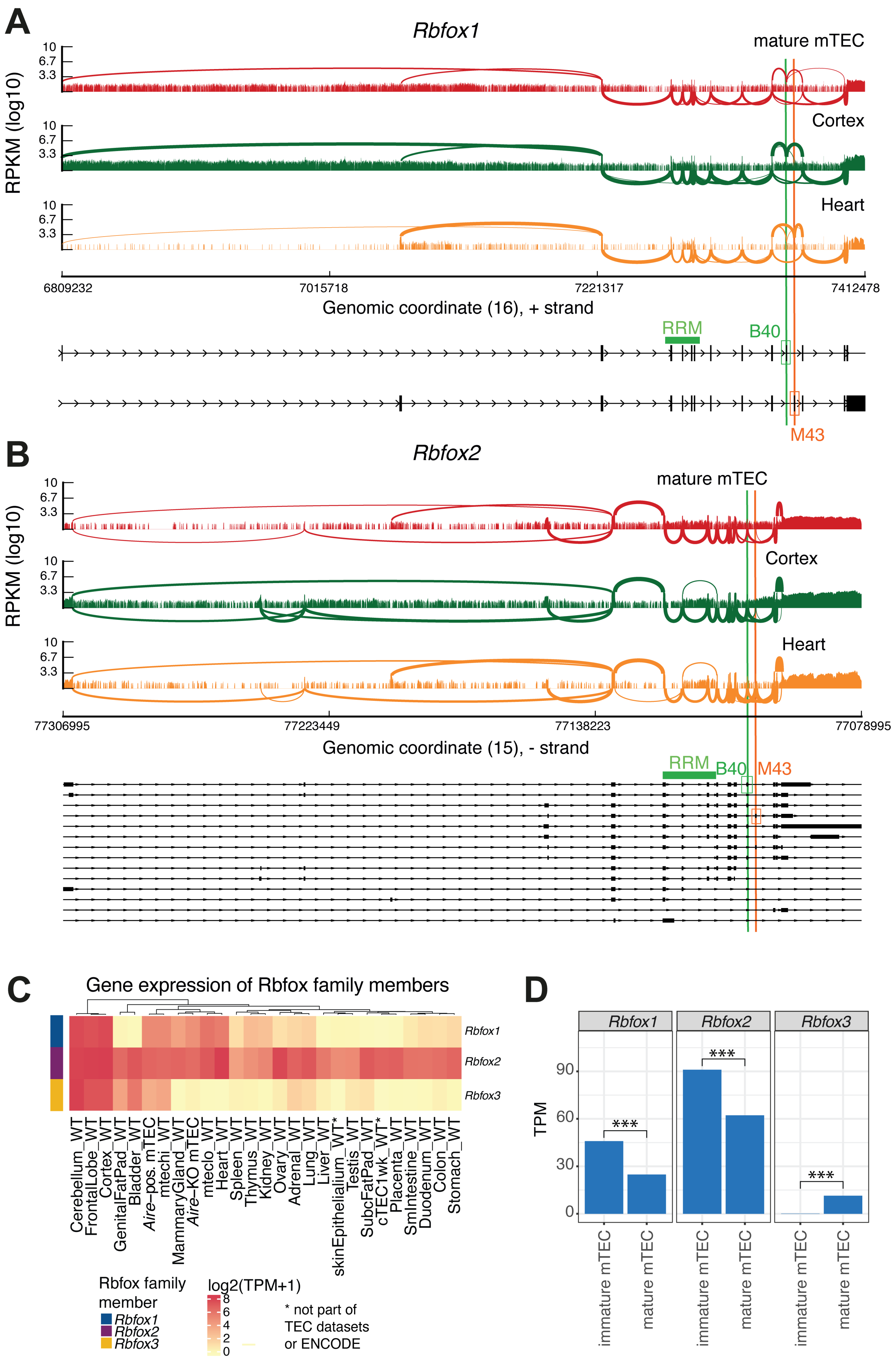
A
Rbfox1
mature mTEC
Cortex
Heart
RPKM (log10)
10
6.7
3.3
6809232
7015718
7221317
7412478
Genomic coordinate (16), + strand
RRM
B40
M43
B
Rbfox2
mature mTEC
Cortex
Heart
RPKM (log10)
77306995
77223449
77138223
77078995
Genomic coordinate (15), - strand
RRM
B40
M43
C
Gene expression of Rbfox family members
Rbfox1
Rbfox2
Rbfox3
Cerebellum_WT
FrontalLobe_WT
Cortex_WT
GenitalFatPad_WT
Bladder_WT
Aire–pos. mTEC
mtechi_WT
MammaryGland_WT
Aire–KO mTEC
mteclo_WT
Heart_WT
Spleen_WT
Thymus_WT
Kidney_WT
Ovary_WT
Adrenal_WT
Lung_WT
Liver_WT
skinEpithelialium_WT*
Testis_WT
SubcFatPad_WT
cTEC1wk_WT*
Placenta_WT
SmIntestine_WT
Duodenum_WT
Colon_WT
Stomach_WT
Rbfox family member
Rbfox1
Rbfox2
Rbfox3
log2(TPM+1)
8
6
4
2
0
* not part of TEC datasets or ENCODE
D
Rbfox1
Rbfox2
Rbfox3
TPM
90
60
30
0
***
immature mTEC
mature mTEC

Supplemental Figure 12: Splicing and expression of the Rbfox family in TEC and peripheral tissues. Assessment of the transcript structures of *Rbfox1* (A) and *Rbfox2* (B) present in the samples from mature mTEC, cortex and heart. Panels show sashimi plots (and read coverage) above the annotated transcript models. The positions of the brain (B40) and muscle (M43) specific exons are indicated. The plots in C and D were generated using MISO (Katz et al. 2010) from a single high-depth, deduplicated sample per tissue. (C) Heatmap of the expression of members of the Rbfox family across mouse peripheral tissue and TEC sub-populations. Columns are hierarchically clustered. (D) The barplots show the expression of *Rbfox1*, *Rbfox2* and *Rbfox3* in wildtype immature and mature mTEC. Stars indicate significant differential expression (DESeq2 comparison of immature versus mature mTEC with high depth, n=2 replicates).

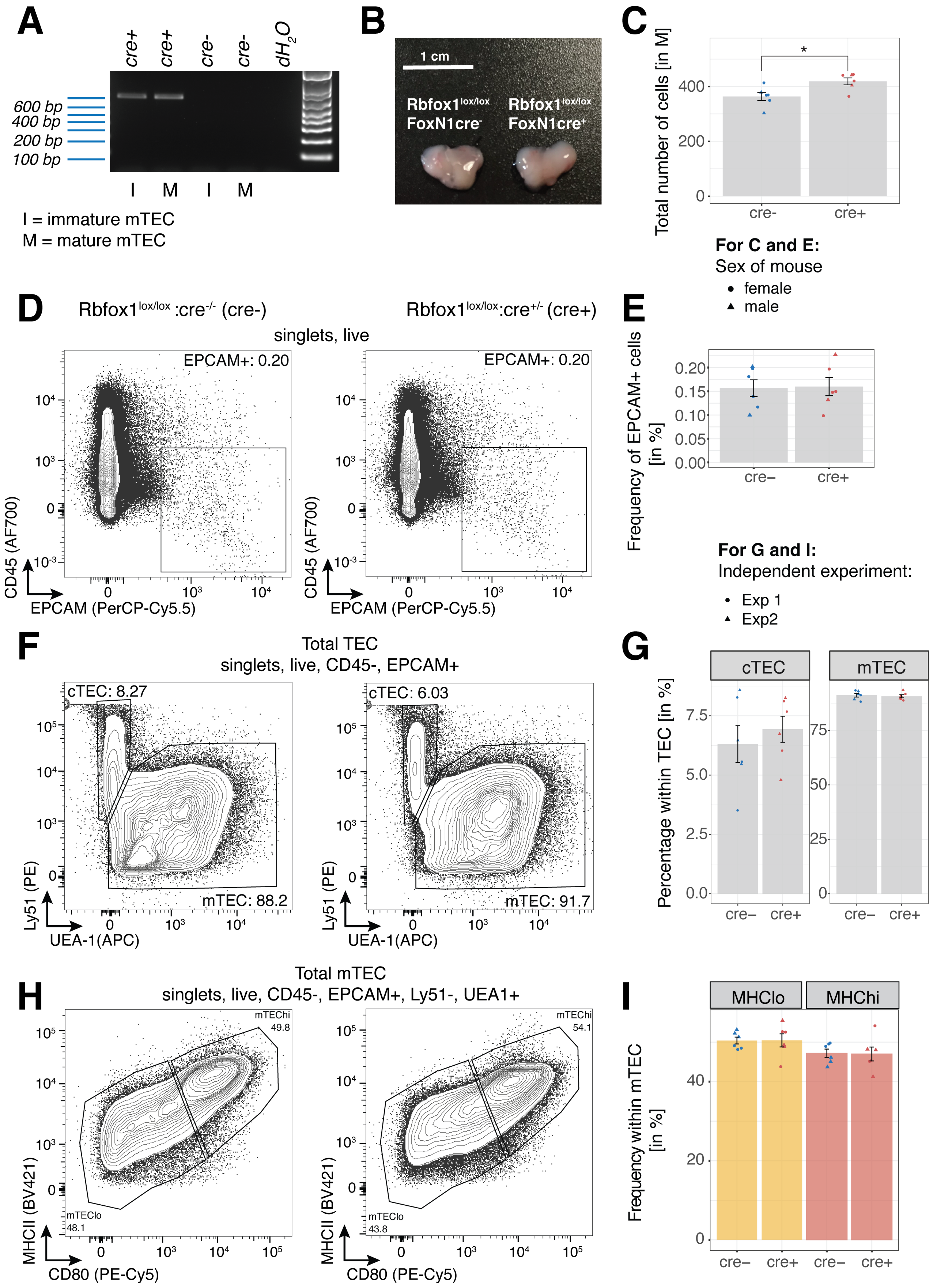

Supplemental Figure 13: Assessment of the phenotype of *Rbfox1* deficient thymi and thymic epithelial cells. *Rbfox1*$^{lox/lox}$:cre-/- (designated cre- and serving as controls) and

Rbfox1$^{lox/lox}$:cre+/- (designated cre+) mice were compared. (A) PCR analysis of genomic exons 11 and 12 of *Rbfox1*. (B) Representative image of gross anatomy of the thymus isolated from *Rbfox1* tKO and control mice. (C) Total thymus cellularity. The individual symbols indicate the sex of each mouse. (D) Gating strategy for the identification of TEC. Representative graphs demonstrate the gating for TEC (EPCAM+, CD45-) among non-enriched thymic cells isolated from the indicated mouse strains. (E) TEC frequencies. (F) Gating strategy for the identification of TEC subpopulations. Representative graphs demonstrate the gating for TEC subpopulations from enriched cells (EPCAM+, CD45-). (G) Frequencies of TEC subpopulations. (H) Gating strategy to identify mTEC subpopulations as defined by MHCII and CD80 expression. (I) Frequencies of mTEC subpopulations. In panels G and I, the two independent experiments are indicated by different symbols in the bar graphs. Data presented in bar graphs represents the combined results from n=2 independent experiments with n=6 mice in each experiment (4-6 weeks old). Significant differences of p-value < 0.05 between Rbfox1$^{lox/lox}$:cre-/- and Rbfox1$^{lox/lox}$:cre+/- using a two-sided Welch Two Sample *t*-test are indicated with * (mean ± SE shown in bar graphs).

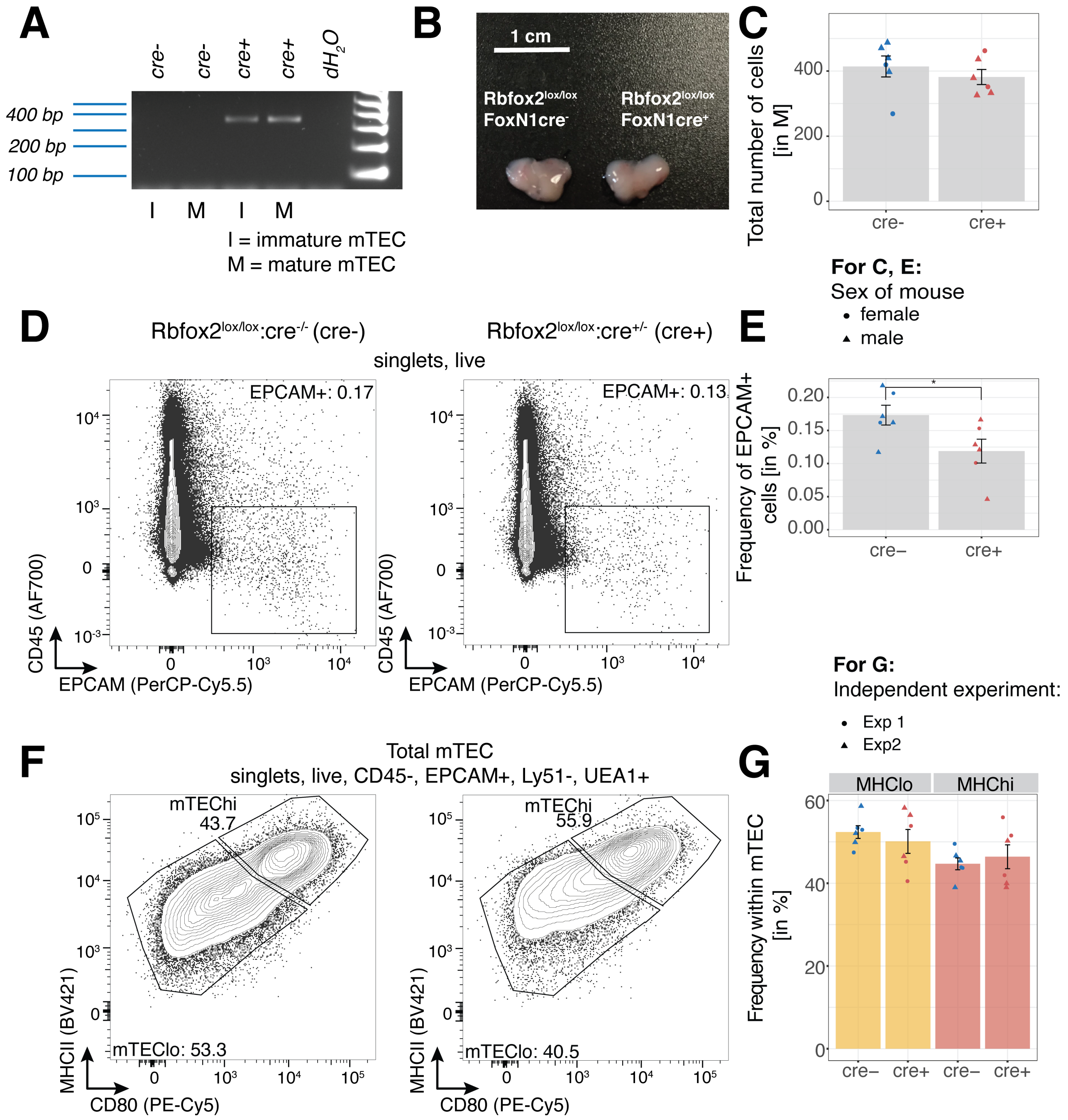

Supplemental Figure 14: Assessment of the phenotype of *Rbfox2* deficient thymi and thymic epithelial cells. *Rbfox2*$^{lox/lox}$:cre-/- (designated cre- and serving as controls) and *Rbfox2*$^{lox/lox}$:cre+/- (designated cre+) mice were compared. (A) PCR analysis of genomic exons 6 and 7 of *Rbfox2*. (B) Representative image of gross anatomy of the thymus isolated from *Rbfox2* tKO and control mice. (C) Total thymus cellularity. The individual symbols indicate the sex of each mouse. (D) Gating strategy for the identification of TEC. Representative graphs demonstrate the gating for TEC (EPCAM+, CD45-) among non-enriched thymic cells isolated from the indicated mouse strains. (E) TEC frequencies. The symbols indicate the sex of each mouse. (F) Gating

strategy for the identification of TEC subpopulations. Representative graphs demonstrate the gating for the mTEC subpopulations as defined by MHCII and CD80 expression (EPCAM+, CD45-, UEA-1+, Ly51-). (G) Frequencies of mTEC subpopulations. Representative graphs for gating of mTEC versus cTEC based on UEA-1 and Ly51 is shown in Fig. 6B. Data presented in bar graphs represents the combined results from a total of two independent experiments. A total of six 4-6 weeks old mice were used per experimental group. Significant differences of p-value < 0.05 between Rbfox2$^{lox/lox}$:cre-/- and Rbfox2$^{lox/lox}$:cre+/- using a two-sided Welch Two Sample *t*-test are indicated with * (mean ± SE shown in bar graphs).

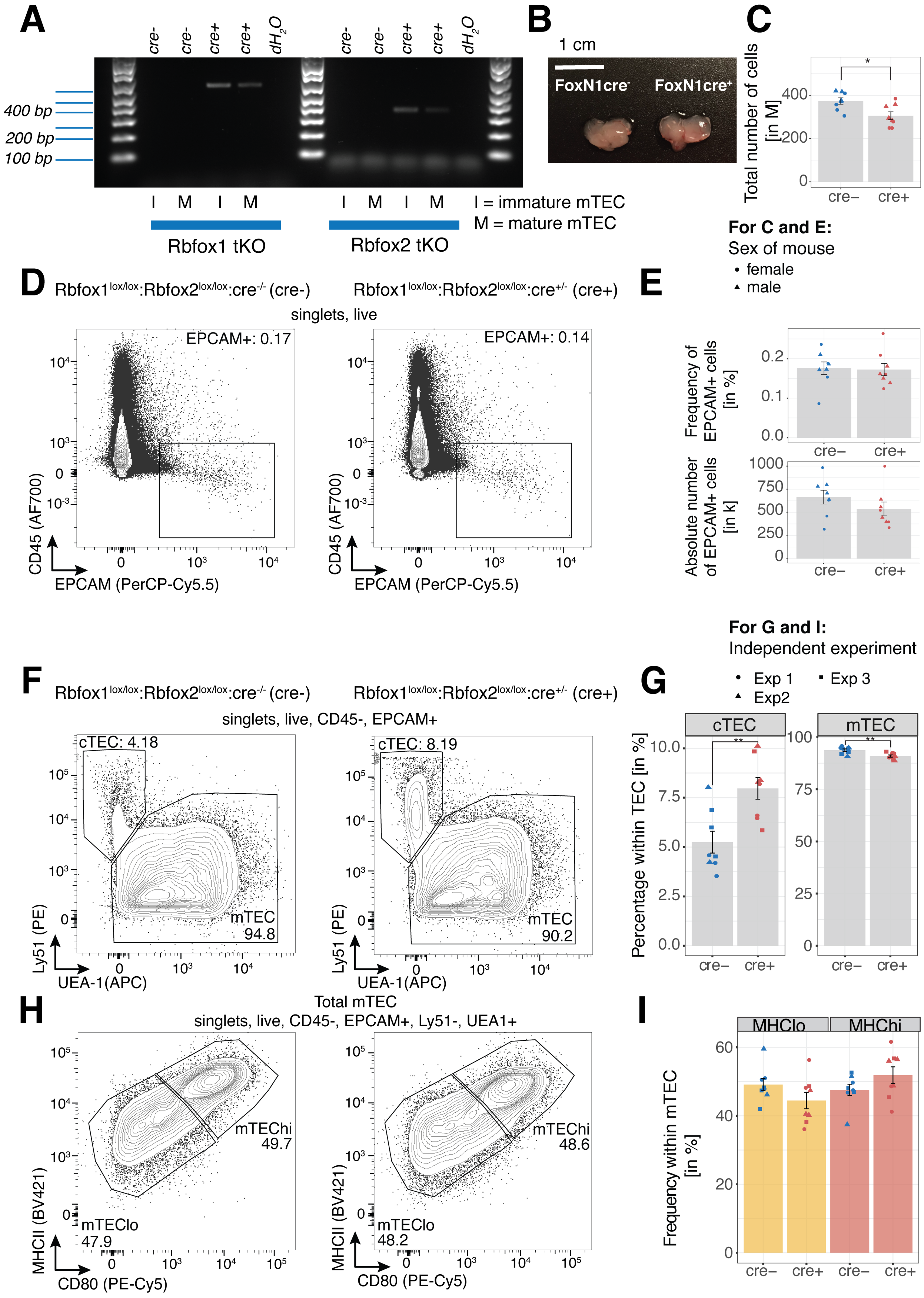

Supplemental Figure 15: Assessment of the phenotype of *Rbfox1* and *Rbfox2* double-knockout thymi and thymic epithelial cells. *Rbfox1*$^{lox/lox}$:*Rbfox2*$^{lox/lox}$:cre-/- (designated cre- and

serving as controls) and *Rbfox1*$^{lox/lox}$: *Rbfox2*$^{lox/lox}$:cre+/- (designated cre+) mice were compared. (A) PCR analysis of genomic exons 11 and 12 of *Rbfox1* (left) and exons 6 and 7 of *Rbfox2* (right). (B) Representative image of gross anatomy of the thymus isolated from *Rbfox1*:*Rbfox2* tKO and control mice. (C) Total thymus cellularity. Symbols indicate the sex of each mouse. (D) Gating strategy for the identification of TEC. Representative graphs demonstrate the gating for TEC (EPCAM+, CD45-) among non-enriched thymic cells isolated from the indicated mouse strains. (E) TEC frequencies. The symbols indicate the sex of each mouse. (F) Gating strategy for the identification of TEC subpopulations. Representative graphs demonstrate the gating for the TEC subpopulations from enriched cells (EPCAM+, CD45-). (G) Frequencies of TEC subpopulations. (H) Gating strategy for mTEC subpopulations as defined by MHCII and CD80 expression. (I) Frequencies of mTEC subpopulations. In panels G and I, the three independent experiments are indicated by different symbols. Data presented in bar graphs represents the combined results from a total of three independent experiments. A total of eight 4-6 weeks old mice were used per experimental group (sex-matched). Significant differences of p-value < 0.05 or < 0.01 between Rbfox1$^{lox/lox}$:Rbfox2$^{lox/lox}$:cre-/- and Rbfox1$^{lox/lox}$:Rbfox2$^{lox/lox}$:cre+/- using a two-sided Welch Two Sample *t*-test are indicated with * or **, respectively (mean ± SE shown in bar graphs).

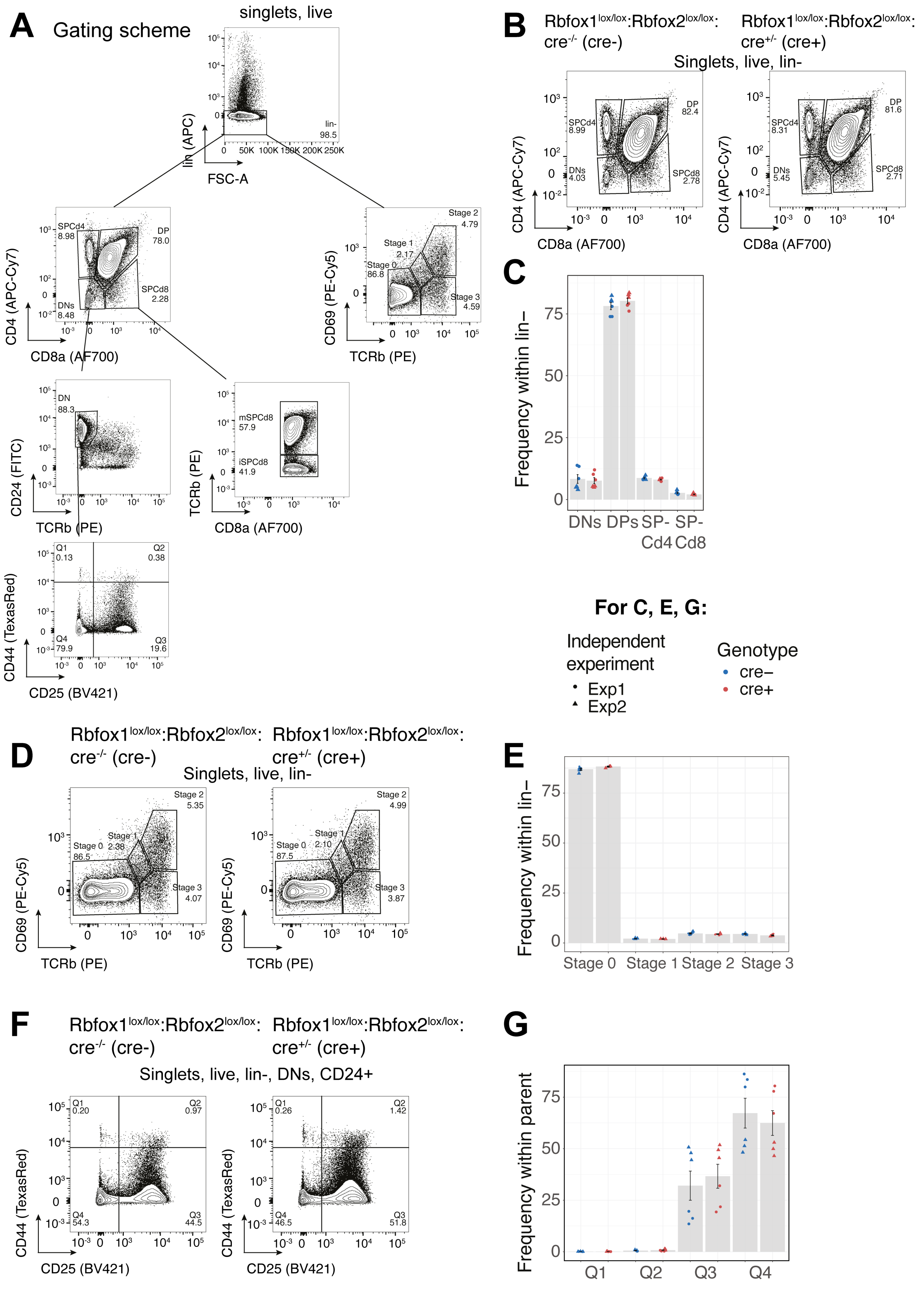

Supplemental Figure 16: Assessment of the phenotype of developing thymocytes from *Rbfox1* and *Rbfox2* double knockout thymi. *Rbfox1*$^{lox/lox}$:*Rbfox2*$^{lox/lox}$:cre-/- (designated cre- and

serving as controls) and *Rbfox1*$^{lox/lox}$: *Rbfox2*$^{lox/lox}$:cre+/- (designated cre+) mice were compared. (A) Gating strategy to define developmental stages and selection of thymocytes within the thymus. Representative graphs demonstrate the gating for different thymocyte subpopulations. DN = double-negatives, SP = single-positive, DP = double-positive. (B) Representative graphs demonstrate the gatings for CD4 and CD8 amongst total, live thymocytes. The gates define CD4 single-positive (SPCd4), CD8 single-positive (SPCd8), double-negative (DNs) and double-positive (DP) thymocytes. (C) Frequencies of thymocyte subpopulations as defined by gating in (B) from two independent experiments. (D) Representative graphs to demonstrate the gating for thymocyte subpopulations defined by CD69 and TCR-β expression. The total, live thymocytes from the two genotypes were included in the gating. The stages represent positive selection of thymocytes, which is characterised by upregulation of CD69 indicating an interaction between the TCR and MHC molecules. (E) Frequencies of thymocyte subpopulations as defined by gating in panel D from two independent experiments. (F) Representative graph demonstrating gating for CD44 and CD25 in CD24+ double-negative thymocytes. The four quadrants represent the double-negative stages, where Q1 corresponds to DN1 (CD44+CD25-), Q2 to DN2 (CD44+CD25+), Q3 to DN3 (CD44-CD25+) and Q4 to DN4 (CD44-CD25-). (G) Frequencies of cells in DN stages as defined by gating in (F) from two independent experiments.

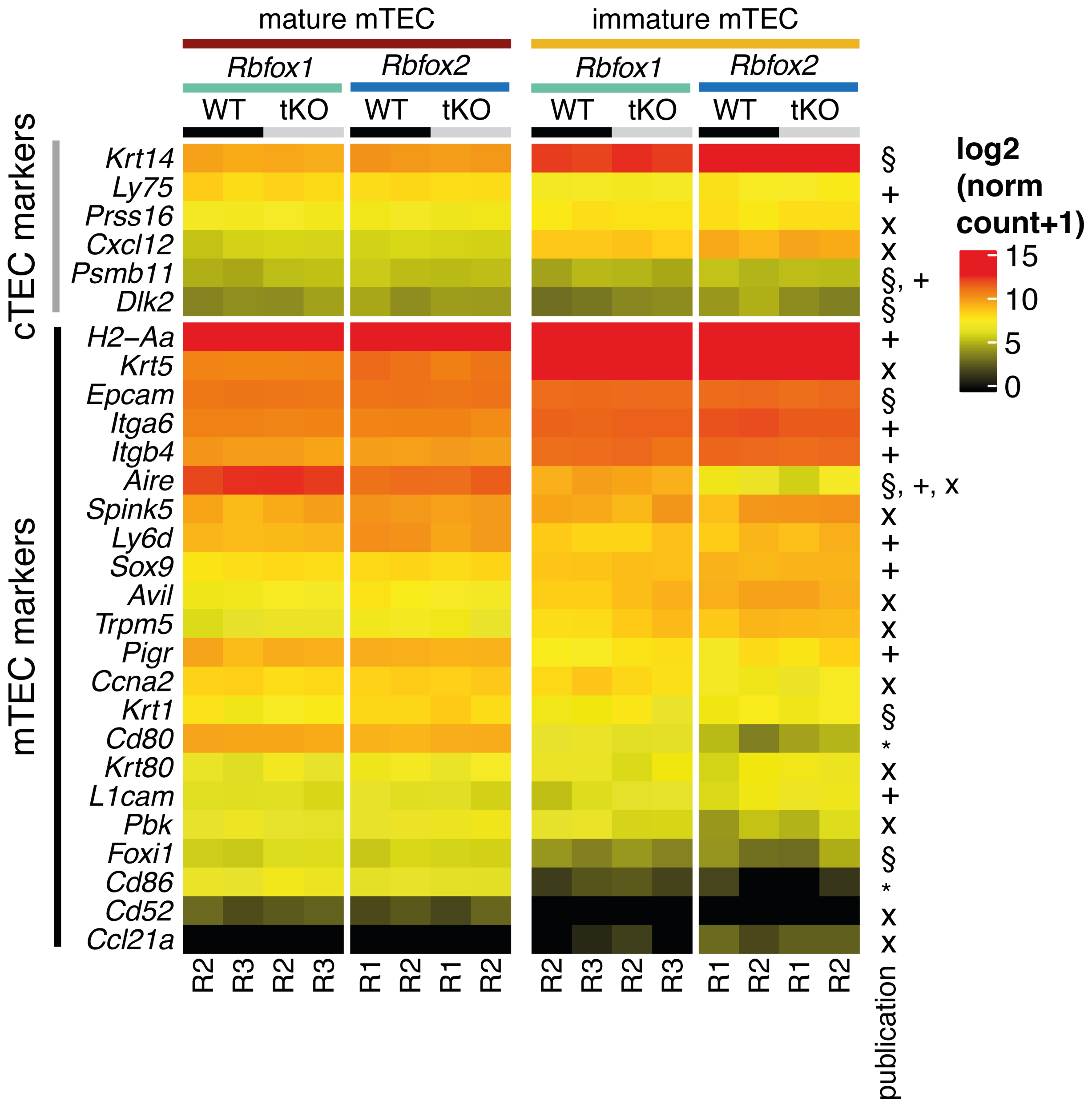

Supplemental Figure 17: **The expression of mTEC maturation and sub-population markers is not changed in *Rbfox1* or *Rbfox2* tKO mice**. The heatmap shows the expression of known TEC maturation and sub-population markers in immature and mature mTEC from *Rbfox1* and *Rbfox2* tKO mice and wildtype littermates. The set of markers was compiled from: [x] (Baran-Gale et al. 2020), [§] (Park et al. 2020) , [+] (Bornstein et al. 2018), and [*] other commonly used TEC phenotype markers (Kadouri et al. 2020).

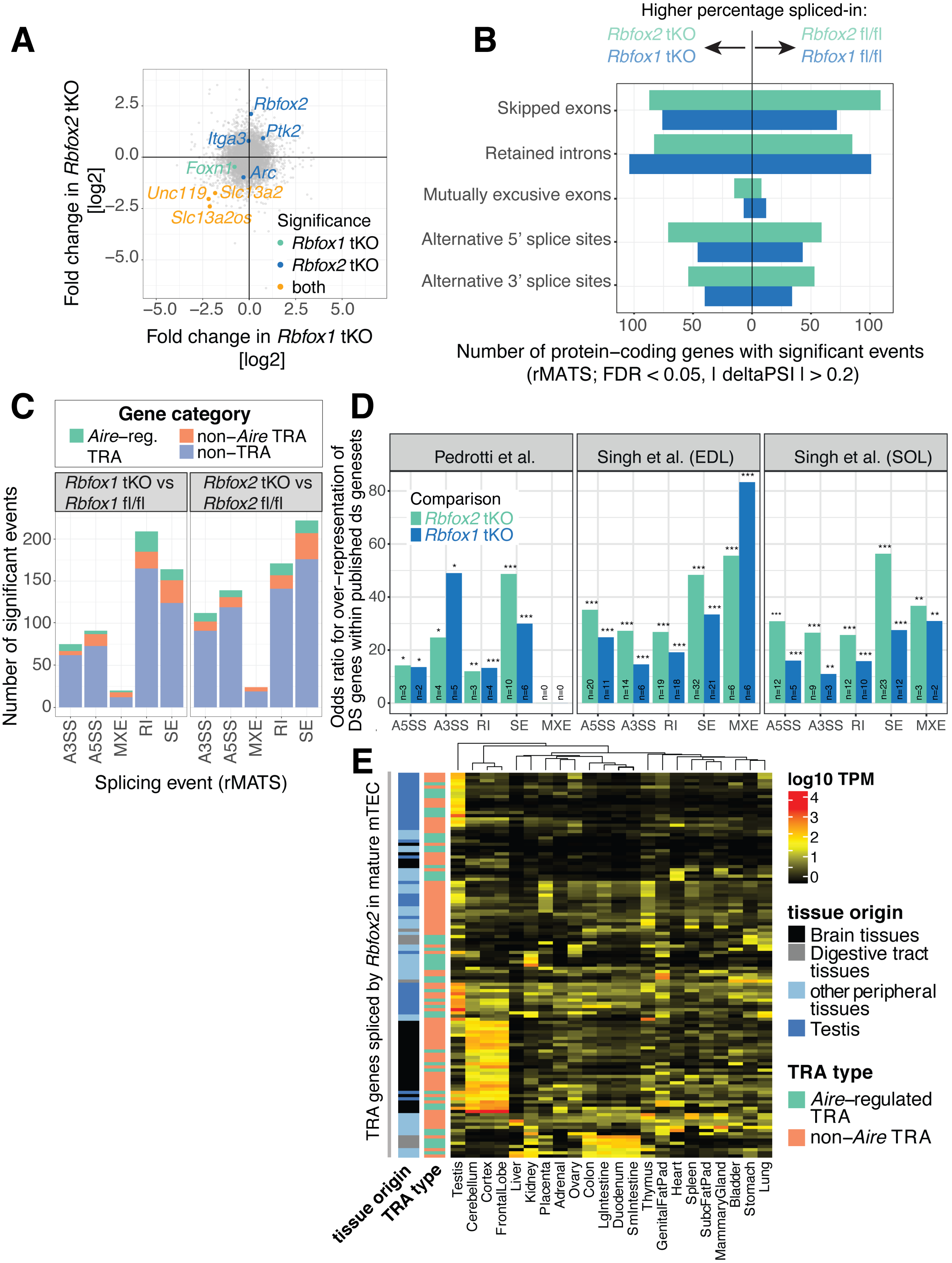

Supplemental Figure 18: Differential gene expression and differential splicing in mature mTEC from *Rbfox1* tKO and *Rbfox2* tKO. (A) A small number of genes show differential expression in mature mTEC from *Rbfox1* tKO and *Rbfox2* tKO animals relative to littermate

controls. The scatterplot shows changes in gene expression between the *Rbfox1* tKO vs *Rbfox1*$^{fl/fl}$ mature mTEC (x axis) and *Rbfox2* tKO vs *Rbfox2*$^{fl/fl}$ mature mTEC (y axis). Color codes: Genes significantly differentially expressed in both knockouts are shown in orange, only in the *Rbfox1* tKO mice in green and exclusively in the *Rbfox2* tKO in blue. n=2 biological replicates, DESeq2, BH adjusted $p < 0.05$, $|fc| > 1.5$. (B). The numbers of protein-coding genes containing differential splicing events identified in mature mTEC extracted from *Rbfox1* tKO and *Rbfox2* tKO mice vs cre- littermate controls (n=2 biological replicates, rMATS, FDR < 0.05, |delta PSI| > 0.2). (C) Breakdown of the identified splicing events panel B by event type and gene category. The gene categories are defined in Supplemental Fig. 3. (D) The barplots show the enrichments (ORs) of sets of known Rbfox target genes (Pedrotti et al. 2015; Singh et al. 2018) in the sets of genes differentially spliced in the *Rbfox1* tKO or *Rbfox2* tKO mature mTEC (Two-sided FETs, BH adjusted p-values). * p<0.05, ** p<0.01, *** p<0.001; SE=skipped exon, RI=retained intron, MXE=mutually exclusive exon, A3SS/A5SS=alternative 3'/5' splice site. (E) Expression of TRA genes with events spliced by *Rbfox2* in mature mTEC*.* The heatmap shows the expression of all TRA genes with differential splicing events in *Rbfox2* tKO vs littermate controls across the peripheral tissues. The colored side bar indicates the type of peripheral tissue with the highest expression and *Aire* regulation status.

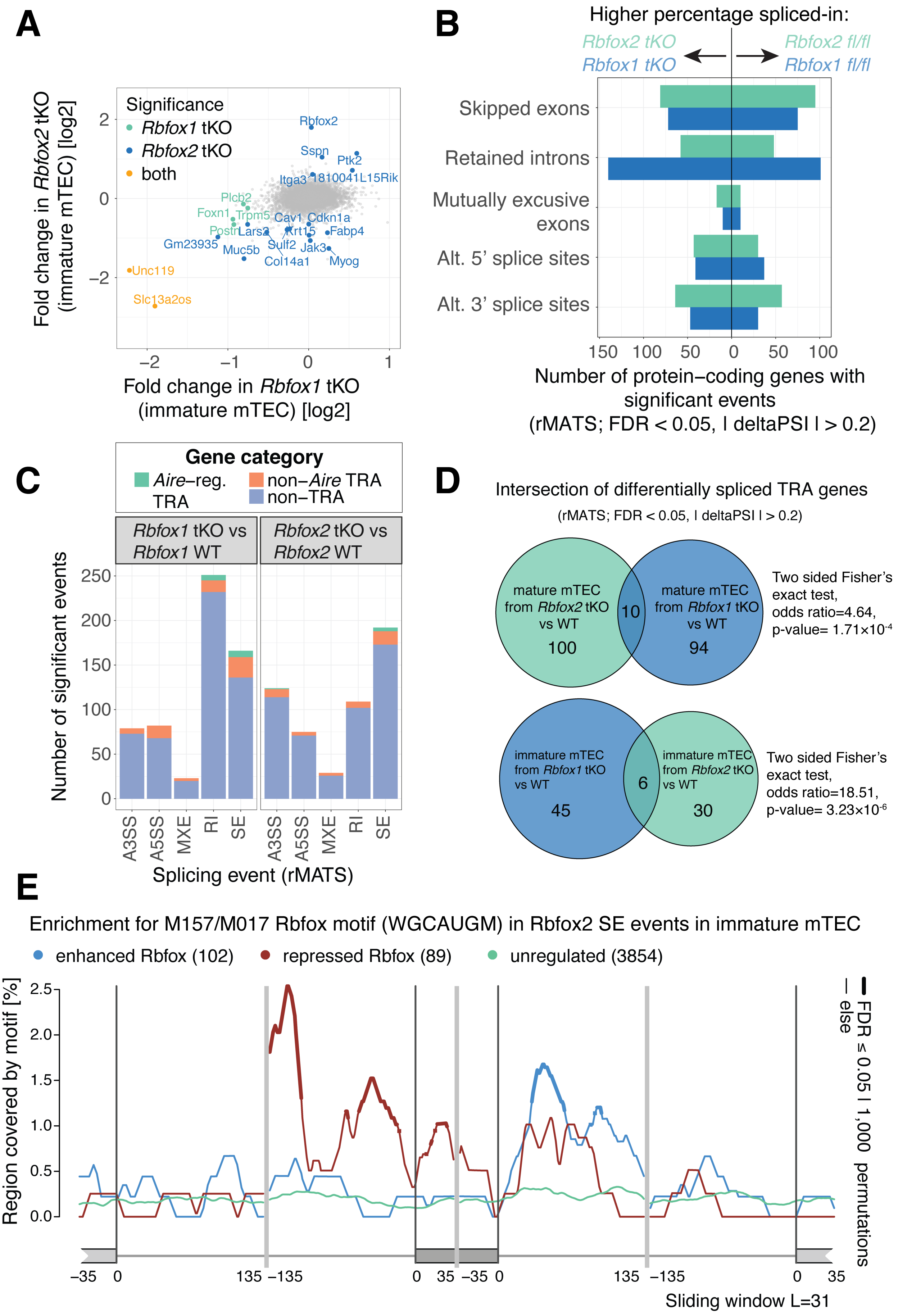
A
Significance
Rbfox1 tKO
Rbfox2 tKO
both
Fold change in Rbfox2 tKO (immature mTEC) [log2]
Fold change in Rbfox1 tKO (immature mTEC) [log2]
Rbfox2
Sspn
Ptk2
Itga3
1810041L15Rik
Plcb2
Foxn1
Trpm5
Cav1
Cdkn1a
Postn
Lars2
Krt15
Fabp4
Gm23935
Muc5b
Sulf2
Jak3
Col14a1
Myog
Unc119
Slc13a2os
B
Higher percentage spliced-in:
Rbfox2 tKO
Rbfox1 tKO
Rbfox2 fl/fl
Rbfox1 fl/fl
Skipped exons
Retained introns
Mutually excusive exons
Alt. 5' splice sites
Alt. 3' splice sites
Number of protein−coding genes with significant events (rMATS; FDR < 0.05, I deltaPSI I > 0.2)
C
Gene category
Aire−reg. TRA
non−Aire TRA
non−TRA
Rbfox1 tKO vs Rbfox1 WT
Rbfox2 tKO vs Rbfox2 WT
Number of significant events
A3SS
A5SS
MXE
RI
SE
Splicing event (rMATS)
D
Intersection of differentially spliced TRA genes
(rMATS; FDR < 0.05, I deltaPSI I > 0.2)
mature mTEC from Rbfox2 tKO vs WT
100
10
mature mTEC from Rbfox1 tKO vs WT
94
Two sided Fisher's exact test, odds ratio=4.64, p-value= 1.71x10-4
immature mTEC from Rbfox1 tKO vs WT
45
6
immature mTEC from Rbfox2 tKO vs WT
30
Two sided Fisher's exact test, odds ratio=18.51, p-value= 3.23x10-6
E
Enrichment for M157/M017 Rbfox motif (WGCAUGM) in Rbfox2 SE events in immature mTEC
enhanced Rbfox (102)
repressed Rbfox (89)
unregulated (3854)
Region covered by motif [%]
FDR ≤ 0.05 I 1,000 permutations
else
Sliding window L=31

Supplemental Figure 19: Differential gene expression and splicing in immature mTEC from *Rbfox1* tKO and *Rbfox2* tKO. (A) The scatterplot shows changes in gene expression between the immature mTEC from *Rbfox1* tKO vs cre- littermates (x axis) and immature mTEC from Rbfox2 tKO vs cre- littermates (y axis). Color codes: Genes significantly differentially expressed in both knockouts are shown in orange, only in the *Rbfox1* tKO mice in green and exclusively in the *Rbfox2* tKO in blue. Two biological replicates, DESeq2, BH adjusted $p < 0.05$, $|fc| > 1.5$. (B) The numbers of protein-coding genes containing significantly differentially spliced events identified in immature mTEC extracted from *Rbfox1* tKO and *Rbfox2* tKO mice vs cre- littermate controls (n=2 biological replicates, rMATS, FDR < 0.05, |delta (d)PSI| > 0.2). (C) Breakdown of the identified splicing events in panel B by event type and gene category. The gene categories are defined in Supplemental Fig. 3. (D) Venn diagram showing the overlap between TRA genes with significant differential splicing events in mature mTEC (top) and immature mTEC (bottom). (E) Enrichment of the M159/M017 Rbfox RRM recognition motif in the sequence around exons found to be significantly regulated by Rbfox2 in immature mTEC. The lines show enrichments for the sets of exons that were found to be enhanced (blue) or repressed (red) or not significantly regulated (green) by *Rbfox2*. The thresholds used were |dPSI| > 0.2 and FDR < 0.05. Thicker lines indicate regions of statistically significant enrichment (FDR ≤ 0.05, n=1,000 permutations).

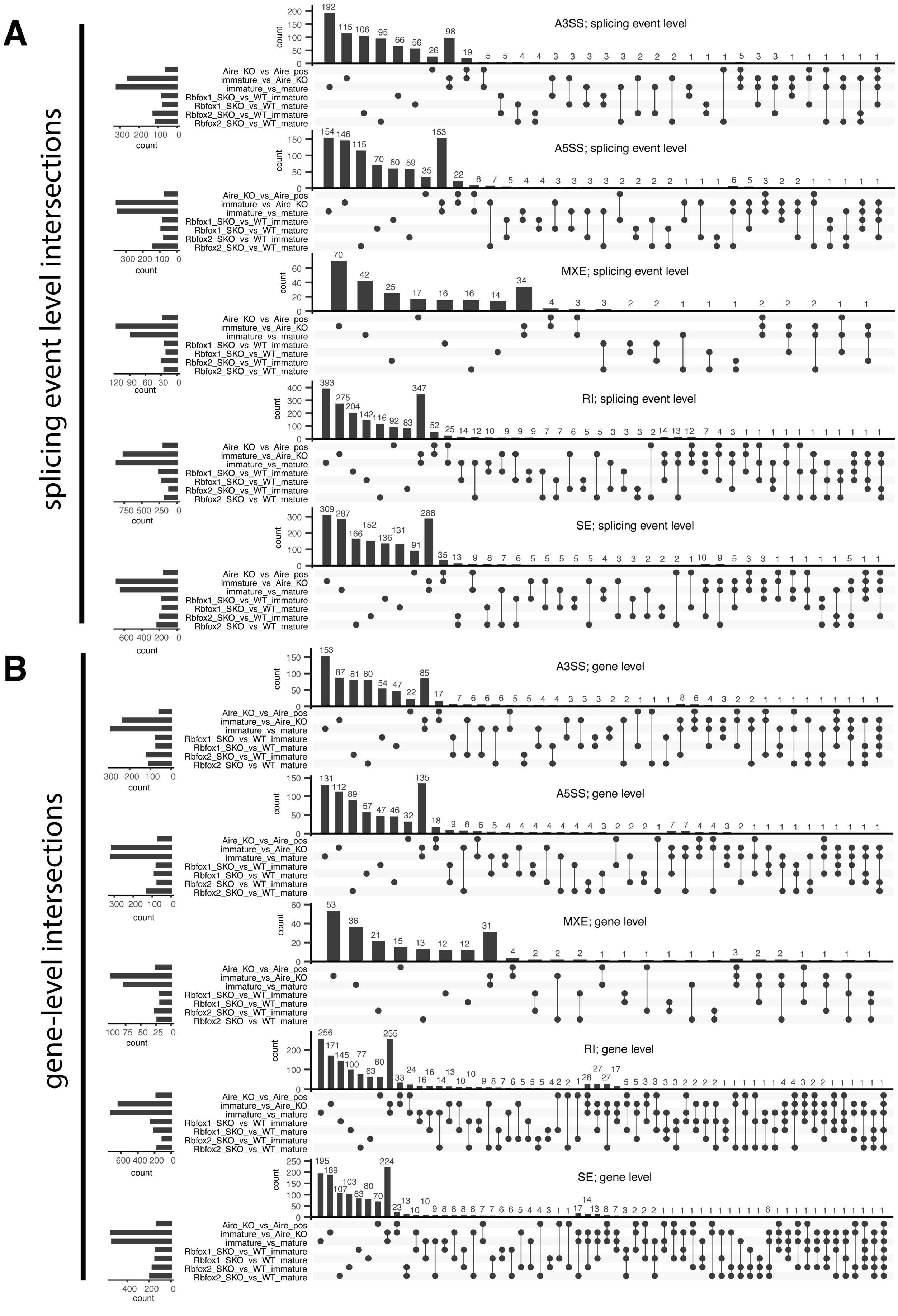
A
splicing event level intersections
A3SS; splicing event level
A5SS; splicing event level
MXE; splicing event level
RI; splicing event level
SE; splicing event level
B
gene-level intersections
A3SS; gene level
A5SS; gene level
MXE; gene level
RI; gene level
SE; gene level
Aire_KO_vs_Aire_pos
immature_vs_Aire_KO
immature_vs_mature
Rbfox1_SKO_vs_WT_immature
Rbfox1_SKO_vs_WT_mature
Rbfox2_SKO_vs_WT_immature
Rbfox2_SKO_vs_WT_mature
count

Supplemental Figure 20: Intersection of Rbfox1, Rbfox2, Aire and maturity associated splicing events in mTEC. The upset plots show the overlaps between significant differences in splicing identified in rMATS analyses performed for this study. The intersections are shown at the event location (A) and gene (B) levels. The different splicing event types are shown in the separate sub-panels. SE=skipped exon, MXE=mutually exclusive exon, RI=retained intron, A3SS/A5SS=alternative 3'/5' splice site.

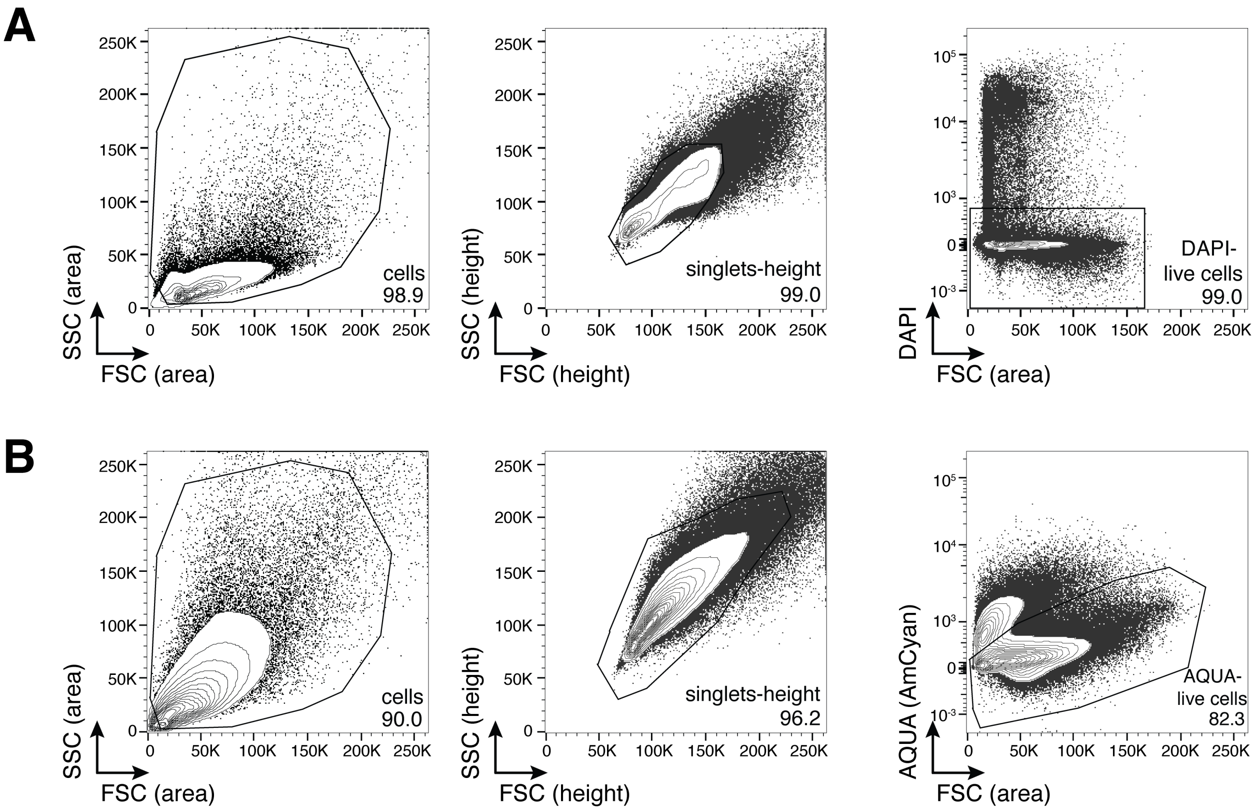

Supplemental Figure 21: The gating strategy for isolation of live cells. (A) Representative gating to define live cells using the absence of DAPI staining. The gating strategy includes first the gating for cells based on FSC (area) and SSC (area), then the gating for single cells based on FSC (height) and SSC (height) and finally the selection of live cells based on the absence of DAPI staining (from left to right). (B) Representative gating to define live cells using the absence of AQUA staining. The gating strategy first includes the gating for cells based on FSC (area) and SSC (area), then the gating of single cells based on FSC (height) and SSC (height) and finally the selection of live cells based on the absence of AQUA staining.

Supplemental Tables

Tissue	Data source	Genotype	No. bio-replicates	High-depth sample	Replicate pool 1	Replicate pool 2
Adrenal	mouse ENCODE Project	wildtype	6	all replicates	R1; R5	R2; R4
Colon		wildtype	6	all replicates	R1; R4	R2; R5
Duodenum		wildtype	7	all replicates	R1; R5	R2; R3
Genital Fat Pad		wildtype	4	all replicates	R3	R4
Heart		wildtype	3	all replicates	R1	R3
Kidney		wildtype	6	all replicates	R1	R4
Liver		wildtype	6	all replicates	R1; R6	R3; R4
Lung		wildtype	4	all replicates	R1	R2; R3
Mammary Gland		wildtype	6	all replicates	R3	R2; R6
Ovary		wildtype	9	all replicates	R1; R10; R2	R4; R5; R6; R7
Small Intestine		wildtype	9	all replicates	R1; R2	R5; R6; R7
Spleen		wildtype	6	all replicates	R1; R2	R3; R4
Stomach		wildtype	6	all replicates	R4; R5	R1; R2
Subc Fat Pad		wildtype	4	all replicates	R1	R4
Testis		wildtype	4	all replicates	R1	R3
Thymus		wildtype	6	all replicates	R4	R2; R3
Frontal Lobe		wildtype	2	all replicates	R1	R2
Bladder		wildtype	2	all replicates	R1	R2
Cortex		wildtype	2	all replicates	R1	R2
Placenta		wildtype	2	all replicates	R1	R2
Cerebellum		wildtype	2	all replicates	R1	R2
Immature mTEC	this study	wildtype	2	all replicates	R1	R2
Mature mTEC		wildtype	2	all replicates	R1	R2
Aire-positive mTEC		*Aire*$^{wildtype/GFP}$ (GFP+ve)	2	all replicates	R1	R2
Aire-knockout mTEC		*Aire*$^{GFP/GFP}$ (GFP+ve)	2	all replicates	R1	R2

Supplemental Table 1: Summary of samples used for the mT&T assembly. The “High-depth sample”, “Replicate pool 1” and “Replicate pool 2” columns give the identifiers of the bio-replicates that were merged to generate the 200M read high-depth samples and the two 60M read replicate pools. To maintain biological replication the two 60M replicate pools were constructed from non-overlapping sets.

Supplemental Table 2: Classification of protein-coding genes according to their *Aire*-regulation status and tissue-specificity.

(Supplemental_Tables.xlsx)

Supplemental Table 3: Novel transcripts specific to mature mTEC (Fig. 3B-C).

(Supplemental_Tables.xlsx)

Supplemental Table 4: Gene ontology categories enriched in protein-coding genes with TEC-specific novel transcripts (Fig. 3D).

(Supplemental_Tables.xlsx)

Supplemental Table 5: Splicing events associated with maturation and *Aire* in TEC (Fig. 4A-B).

(Supplemental_Tables.xlsx)

Supplemental Table 6: *Aire* regulated transcripts in mTEC (Fig. 4C-D).

(Supplemental_Tables.xlsx)

Supplemental Table 7: Compilation of splicing-related genes (Supplemental Fig. 10B, 11A).

(Supplemental_Tables.xlsx)

Splicing factor	Tissue described in	Reference (PMID)
Brdt	Testis	15261828
Celf4, Celf6	Neurons	22180311
Cwc22	involved in spliceosome	23236153
Elavl2, Elavl3, Elavl4	Neurons	9096138
Esrp1	Epithelium	26371508
Khdrbs2	Neurons	24469635
Mbnl3	Muscle	25183524
Msi1	NSC, cancer EMT, photoreceptors	25380226, 27541351
Nova1, Nova2	Neurons	17065982
Rbfox1, Rbfox3	Neurons	27748060
Rbm11	Neurons, Testis	21984414
Rbm20, Rbm24	Muscle, Heart	22466703, 25313962
Slu7	Liver	24865429
Snrpn	embryo, neuronal (imprinted region)	25238490
Srpk2	Testis	18653532
Srpk3	Muscle, Heart, Spleen	16140986
Srrm4	Neurons	25525873, 25838543
Syncrip	Neurons	30649277

Supplemental Table 8: Tissue restricted and functionally investigated splicing factors (Fig. 5A).

Cell type	Flow cytometry marker profile
TEC	CD45-EpCAM+
cTEC	CD45-EpCAM+Ly51+ve, UEA-1-ve
mTEC	CD45-EpCAM+Ly51-ve, UEA-1+ve
Immature mTEC	CD45-EpCAM+Ly51-ve, UEA-1+ve CD80-low, MHCII-low
Mature mTEC	CD45-EpCAM+Ly51-ve, UEA-1+ve CD80-high, MHCII-high, either AIRE+ or AIRE-

Supplemental Table 9: The FACS phenotypes of the isolated TEC populations.

Supplemental Table 10: Genes regulated by *Rbfox1* and *Rbfox2* in mTEC (Supplemental Fig. 18A).

(Supplemental_Tables.xlsx)

Supplemental Table 11: Splicing events regulated by *Rbfox1* and *Rbfox2* in immature and mature mTEC (Fig. 7, Supplemental Figures 18, 19).

(Supplemental_Tables.xlsx)

Antigen	Fluorophore	Concentration	Clone	Company
CD8a	AF700	1/200	53-6.7	BioLegend
CD4	APC-Cy7	1/1000	RM4-5	BioLegend
TCRb	PE	1/1000	H57-597	eBioscience
CD24	FITC	1/1000	M1/69	BioLegend
CD25	eFluor450	1/500	PC61.5	eBioscience
CD44	PE TR (PE eFluor 605)	1/1000	IM7	eBioscience
CD69	PE-Cy5	1/1000	H1.2F3	BioLegend
Streptavidin	APC	1/1000		BioLegend
CD11b	Biotin	1/1000	M1/70	BioLegend
CD11c	Biotin	1/1000	N418	BioLegend
Gr1	Biotin	1/1000	RB6-8C5	BioLegend
CD19	Biotin	1/1000	1D3	eBioscience
CD49b	Biotin	1/1000	DX5	BioLegend
F4/80	Biotin	1/1000	BM8	BioLegend
NK1.1	Biotin	1/500	PK136	BioLegend
TCRgd	Biotin	1/1000	eBioGL3 (GL-3, GL3)	eBioscience
Ter119	Biotin	1/1000	TER-119	BioLegend

Supplemental Table 12: List of antibodies used for Supplemental Fig. 16. The columns indicate the target antigen, the conjugated fluorophore, the concentration used in experiments, the antibody clone and the company.

gene_type	tissue_a	tissue_b	wilcox_p	mean_frac_tissue_a	mean_frac_mTEC

Aire-regulated TRA	GenitalFatPad_WT	mature mTEC	0 ***	0.80	0.58
Aire-regulated TRA	Testis_WT	mature mTEC	0 ***	0.78	0.58
Aire-regulated TRA	Liver_WT	mature mTEC	0 ***	0.84	0.58
Aire-regulated TRA	Lung_WT	mature mTEC	0 ***	0.81	0.58
Aire-regulated TRA	Spleen_WT	mature mTEC	0 ***	0.76	0.58
Aire-regulated TRA	FrontalLobe_WT	mature mTEC	0 ***	0.74	0.58
Aire-regulated TRA	Cerebellum_WT	mature mTEC	0 ***	0.76	0.58
Aire-regulated TRA	Colon_WT	mature mTEC	0 ***	0.77	0.58
Aire-regulated TRA	Adrenal_WT	mature mTEC	0 ***	0.82	0.58
Aire-regulated TRA	Duodenum_WT	mature mTEC	0 ***	0.82	0.58
Aire-regulated TRA	Cortex_WT	mature mTEC	0 ***	0.77	0.58
Aire-regulated TRA	Kidney_WT	mature mTEC	0 ***	0.82	0.58
Aire-regulated TRA	Thymus_WT	mature mTEC	0.941842	0.58	0.58
Aire-regulated TRA	MammaryGland_WT	mature mTEC	2.7e-05 ***	0.72	0.58
Aire-regulated TRA	SmIntestine_WT	mature mTEC	0 ***	0.79	0.58
Aire-regulated TRA	Bladder_WT	mature mTEC	0 ***	0.79	0.58
Aire-regulated TRA	Stomach_WT	mature mTEC	0 ***	0.80	0.58
Aire-regulated TRA	Ovary_WT	mature mTEC	0.28974	0.60	0.58
Aire-regulated TRA	Heart_WT	mature mTEC	0 ***	0.85	0.58
Aire-regulated TRA	Placenta_WT	mature mTEC	0 ***	0.76	0.58
Aire-regulated TRA	SubcFatPad_WT	mature mTEC	0.002925 **	0.72	0.58
non-*Aire* TRA	GenitalFatPad_WT	mature mTEC	0 ***	0.76	0.58
non-*Aire* TRA	Testis_WT	mature mTEC	0 ***	0.75	0.58
non-*Aire* TRA	Liver_WT	mature mTEC	0 ***	0.78	0.58
non-*Aire* TRA	Lung_WT	mature mTEC	5.2e-05 ***	0.71	0.58
non-*Aire* TRA	Spleen_WT	mature mTEC	0 ***	0.71	0.58
non-*Aire* TRA	FrontalLobe_WT	mature mTEC	0.002428 **	0.64	0.58
non-*Aire* TRA	Cerebellum_WT	mature mTEC	0 ***	0.67	0.58
non-*Aire* TRA	Colon_WT	mature mTEC	0.000149 ***	0.72	0.58
non-*Aire* TRA	Adrenal_WT	mature mTEC	0.750801	0.60	0.58
non-*Aire* TRA	Duodenum_WT	mature mTEC	0.001944 **	0.70	0.58
non-*Aire* TRA	Cortex_WT	mature mTEC	0 ***	0.68	0.58
non-*Aire* TRA	Kidney_WT	mature mTEC	0 ***	0.82	0.58
non-*Aire* TRA	Thymus_WT	mature mTEC	0 ***	0.68	0.58
non-*Aire* TRA	MammaryGland_WT	mature mTEC	0.000605 ***	0.70	0.58
non-*Aire* TRA	SmIntestine_WT	mature mTEC	6.9e-05 ***	0.69	0.58
non-*Aire* TRA	Bladder_WT	mature mTEC	1e-06 ***	0.72	0.58
non-*Aire* TRA	Stomach_WT	mature mTEC	0.000128 ***	0.71	0.58
non-*Aire* TRA	Ovary_WT	mature mTEC	0.007985 **	0.65	0.58
non-*Aire* TRA	Heart_WT	mature mTEC	0 ***	0.78	0.58
non-*Aire* TRA	Placenta_WT	mature mTEC	0 ***	0.73	0.58
non-*Aire* TRA	SubcFatPad_WT	mature mTEC	0.064713 .	0.69	0.58
non-TRA	GenitalFatPad_WT	mature mTEC	0 ***	0.70	0.68
non-TRA	Testis_WT	mature mTEC	0 ***	0.65	0.68

non-TRA	Liver_WT	mature mTEC	0 ***	0.60	0.68
non-TRA	Lung_WT	mature mTEC	0 ***	0.70	0.68
non-TRA	Spleen_WT	mature mTEC	0 ***	0.65	0.68
non-TRA	FrontalLobe_WT	mature mTEC	0 ***	0.66	0.68
non-TRA	Cerebellum_WT	mature mTEC	0.022574 *	0.67	0.68
non-TRA	Colon_WT	mature mTEC	0 ***	0.65	0.68
non-TRA	Adrenal_WT	mature mTEC	0 ***	0.62	0.68
non-TRA	Duodenum_WT	mature mTEC	0 ***	0.59	0.68
non-TRA	Cortex_WT	mature mTEC	0 ***	0.66	0.68
non-TRA	Kidney_WT	mature mTEC	0 ***	0.66	0.68
non-TRA	Thymus_WT	mature mTEC	0.37276	0.68	0.68
non-TRA	MammaryGland_WT	mature mTEC	0.210702	0.68	0.68
non-TRA	SmIntestine_WT	mature mTEC	0 ***	0.60	0.68
non-TRA	Bladder_WT	mature mTEC	4e-06 ***	0.69	0.68
non-TRA	Stomach_WT	mature mTEC	0 ***	0.59	0.68
non-TRA	Ovary_WT	mature mTEC	0.000262 ***	0.67	0.68
non-TRA	Heart_WT	mature mTEC	0 ***	0.63	0.68
non-TRA	Placenta_WT	mature mTEC	1e-05 ***	0.69	0.68
non-TRA	SubcFatPad_WT	mature mTEC	0 ***	0.70	0.68

Supplemental Table 13: The results of two-sided Wilcox tests (Mann Whitney) testing for differences in the fraction of isoforms detected in peripheral tissues vs mature mTEC (Fig. 2A). The mean fraction of isoforms for each peripheral tissue and mature mTEC is reported in columns 5 and 6.

**Analysis**	**Bio-replicates per sample**	**Animals per replicate**	**Sex of mice**	**Age of mice**	**Read depth** (after deduplication and removing unmapped reads)	**RNA seq details**
rMATS and kallisto comparisons of wildtype immature, wildtype mature, *Aire*-knockout and *Aire*-positive mTEC (Fig. 4)	2	multiple	Female	4-6 weeks	130 M	101 bp, stranded, paired-end (dUTP-based protocol)
rMATS analysis of *Rbfox1* and *Rbfox2* conditional knockouts, (immature and mature) mTEC (Figures 6,7)	2	1	Sex-matched littermates, (1 pair of Females, 1 pair of Males for each comparison)	4-6 weeks	14.5 M	150bp, stranded, paired-end protocol including polyA-enrichment (NEB)

Supplemental Table 14: Summary of the samples and RNA-sequencing data used for rMATS and kallisto alternative splicing/differential transcript usage analyses shown in Figures 4, 6 and 7.

Supplemental References

Alamancos GP, Pages A, Trincado JL, Bellora N, Eyras E. 2015. Leveraging transcript quantification for fast computation of alternative splicing profiles. *RNA* **21**: 1521-1531.

Baran-Gale J, Morgan MD, Maio S, Dhalla F, Calvo-Asensio I, Deadman ME, Handel AE, Maynard A, Chen S, Green F et al. 2020. Ageing compromises mouse thymus function and remodels epithelial cell differentiation. *Elife* **9**.

Barbosa-Morais NL, Carmo-Fonseca M, Aparicio S. 2006. Systematic genome-wide annotation of spliceosomal proteins reveals differential gene family expansion. *Genome Res* **16**: 66-77.

Bornstein C, Nevo S, Giladi A, Kadouri N, Pouzolles M, Gerbe F, David E, Machado A, Chuprin A, Toth B et al. 2018. Single-cell mapping of the thymic stroma identifies IL-25-producing tuft epithelial cells. *Nature* **559**: 622-626.

Bray NL, Pimentel H, Melsted P, Pachter L. 2016. Near-optimal probabilistic RNA-seq quantification. *Nat Biotechnol* **34**: 525-527.

Carbon S, Ireland A, Mungall CJ, Shu S, Marshall B, Lewis S, Ami GOH, Web Presence Working G. 2009. AmiGO: online access to ontology and annotation data. *Bioinformatics* **25**: 288-289.

Chen M, Manley JL. 2009. Mechanisms of alternative splicing regulation: insights from molecular and genomics approaches. *Nat Rev Mol Cell Biol* **10**: 741-754.

Chew SL. 1997. Alternative splicing of mRNA as a mode of endocrine regulation. *Trends Endocrinol Metab* **8**: 405-413.

Chuprin A, Avin A, Goldfarb Y, Herzig Y, Levi B, Jacob A, Sela A, Katz S, Grossman M, Guyon C et al. 2015. The deacetylase Sirt1 is an essential regulator of Aire-mediated induction of central immunological tolerance. *Nat Immunol* **16**: 737-745.

Cook KB, Kazan H, Zuberi K, Morris Q, Hughes TR. 2011. RBPDB: a database of RNA-binding specificities. *Nucleic Acids Res* **39**: D301-308.

Danan-Gotthold M, Guyon C, Giraud M, Levanon EY, Abramson J. 2016. Extensive RNA editing and splicing increase immune self-representation diversity in medullary thymic epithelial cells. *Genome Biol* **17**: 219.

de Jonge HJ, Fehrmann RS, de Bont ES, Hofstra RM, Gerbens F, Kamps WA, de Vries EG, van der Zee AG, te Meerman GJ, ter Elst A. 2007. Evidence based selection of housekeeping genes. *PLoS One* **2**: e898.

Dhalla F, Baran-Gale J, Maio S, Chappell L, Hollander GA, Ponting CP. 2020. Biologically indeterminate yet ordered promiscuous gene expression in single medullary thymic epithelial cells. *EMBO J* **39**: e101828.

Dobin A, Davis CA, Schlesinger F, Drenkow J, Zaleski C, Jha S, Batut P, Chaisson M, Gingeras TR. 2013. STAR: ultrafast universal RNA-seq aligner. *Bioinformatics* **29**: 15-21.

Gohr A, Irimia M. 2019. Matt: Unix tools for alternative splicing analysis. *Bioinformatics* **35**: 130-132.

Gromak N, Matlin AJ, Cooper TA, Smith CW. 2003. Antagonistic regulation of alpha-actinin alternative splicing by CELF proteins and polypyrimidine tract binding protein. *RNA* **9**: 443-456.

Grosso AR, Gomes AQ, Barbosa-Morais NL, Caldeira S, Thorne NP, Grech G, von Lindern M, Carmo-Fonseca M. 2008. Tissue-specific splicing factor gene expression signatures. *Nucleic Acids Res* **36**: 4823-4832.

Han H, Irimia M, Ross PJ, Sung HK, Alipanahi B, David L, Golipour A, Gabut M, Michael IP, Nachman EN et al. 2013. MBNL proteins repress ES-cell-specific alternative splicing and reprogramming. *Nature* **498**: 241-245.

Handel AE, Shikama-Dorn N, Zhanybekova S, Maio S, Graedel AN, Zuklys S, Ponting CP, Hollander GA. 2018. Comprehensively Profiling the Chromatin Architecture of Tissue Restricted Antigen Expression in Thymic Epithelial Cells Over Development. *Front Immunol* **9**: 2120.

Jangi M, Sharp PA. 2014. Building robust transcriptomes with master splicing factors. *Cell* **159**: 487-498.

Kadouri N, Nevo S, Goldfarb Y, Abramson J. 2020. Thymic epithelial cell heterogeneity: TEC by TEC. *Nat Rev Immunol* **20**: 239-253.

Katz Y, Wang ET, Airoldi EM, Burge CB. 2010. Analysis and design of RNA sequencing experiments for identifying isoform regulation. *Nat Methods* **7**: 1009-1015.

Keane P, Ceredig R, Seoighe C. 2015. Promiscuous mRNA splicing under the control of AIRE in medullary thymic epithelial cells. *Bioinformatics* **31**: 986-990.

Kim D, Paggi JM, Park C, Bennett C, Salzberg SL. 2019. Graph-based genome alignment and genotyping with HISAT2 and HISAT-genotype. *Nat Biotechnol* **37**: 907-915.

Kryuchkova-Mostacci N, Robinson-Rechavi M. 2017. A benchmark of gene expression tissue-specificity metrics. *Brief Bioinform* **18**: 205-214.

Li H. 2018. Minimap2: pairwise alignment for nucleotide sequences. *Bioinformatics* **34**: 3094-3100.

Li H, Handsaker B, Wysoker A, Fennell T, Ruan J, Homer N, Marth G, Abecasis G, Durbin R, Genome Project Data Processing S. 2009. The Sequence Alignment/Map format and SAMtools. *Bioinformatics* **25**: 2078-2079.

Liao Y, Smyth GK, Shi W. 2014. featureCounts: an efficient general purpose program for assigning sequence reads to genomic features. *Bioinformatics* **30**: 923-930.

Love MI, Huber W, Anders S. 2014. Moderated estimation of fold change and dispersion for RNA-seq data with DESeq2. *Genome Biol* **15**: 550.

Merkin J, Russell C, Chen P, Burge CB. 2012. Evolutionary dynamics of gene and isoform regulation in Mammalian tissues. *Science* **338**: 1593-1599.

Park JE, Botting RA, Dominguez Conde C, Popescu DM, Lavaert M, Kunz DJ, Goh I, Stephenson E, Ragazzini R, Tuck E et al. 2020. A cell atlas of human thymic development defines T cell repertoire formation. *Science* **367**.

Patro R, Duggal G, Love MI, Irizarry RA, Kingsford C. 2017. Salmon provides fast and bias-aware quantification of transcript expression. *Nat Methods* **14**: 417-419.

Pedrotti S, Giudice J, Dagnino-Acosta A, Knoblauch M, Singh RK, Hanna A, Mo Q, Hicks J, Hamilton S, Cooper TA. 2015. The RNA-binding protein Rbfox1 regulates splicing required for skeletal muscle structure and function. *Hum Mol Genet* **24**: 2360-2374.

Pertea M, Pertea GM, Antonescu CM, Chang TC, Mendell JT, Salzberg SL. 2015. StringTie enables improved reconstruction of a transcriptome from RNA-seq reads. *Nat Biotechnol* **33**: 290-295.

Pervouchine DD, Djebali S, Breschi A, Davis CA, Barja PP, Dobin A, Tanzer A, Lagarde J, Zaleski C, See LH et al. 2015. Enhanced transcriptome maps from multiple mouse tissues reveal evolutionary constraint in gene expression. *Nat Commun* **6**: 5903.

Pervouchine DD, Knowles DG, Guigo R. 2013. Intron-centric estimation of alternative splicing from RNA-seq data. *Bioinformatics* **29**: 273-274.

Pimentel H, Bray NL, Puente S, Melsted P, Pachter L. 2017. Differential analysis of RNA-seq incorporating quantification uncertainty. *Nat Methods* **14**: 687-690.

Sansom SN, Shikama-Dorn N, Zhanybekova S, Nusspaumer G, Macaulay IC, Deadman ME, Heger A, Ponting CP, Hollander GA. 2014. Population and single-cell genomics reveal the Aire dependency, relief from Polycomb silencing, and distribution of self-antigen expression in thymic epithelia. *Genome Res* **24**: 1918-1931.

Shen S, Park JW, Lu ZX, Lin L, Henry MD, Wu YN, Zhou Q, Xing Y. 2014. rMATS: robust and flexible detection of differential alternative splicing from replicate RNA-Seq data. *Proc Natl Acad Sci U S A* **111**: E5593-5601.

Singh RK, Kolonin AM, Fiorotto ML, Cooper TA. 2018. Rbfox-Splicing Factors Maintain Skeletal Muscle Mass by Regulating Calpain3 and Proteostasis. *Cell Rep* **24**: 197-208.

St-Pierre C, Brochu S, Vanegas JR, Dumont-Lagace M, Lemieux S, Perreault C. 2013. Transcriptome sequencing of neonatal thymic epithelial cells. *Sci Rep* **3**: 1860.
St-Pierre C, Trofimov A, Brochu S, Lemieux S, Perreault C. 2015. Differential Features of AIRE-Induced and AIRE-Independent Promiscuous Gene Expression in Thymic Epithelial Cells. *J Immunol* **195**: 498-506.
